# 3-Amino-Substituted Analogues of Fusidic Acid as Membrane-Active Antibacterial Compounds

**DOI:** 10.3390/membranes13030309

**Published:** 2023-03-07

**Authors:** Elena V. Salimova, Oleg S. Mozgovoj, Svetlana S. Efimova, Olga S. Ostroumova, Lyudmila V. Parfenova

**Affiliations:** 1 Institute of Petrochemistry and Catalysis, Ufa Federal Research Center, Russian Academy of Sciences, 141 Prospect Oktyabrya, 450075 Ufa, Russia; 2 Institute of Cytology of Russian Academy of Sciences, 4 Tikhoretsky Prospect, 194064 Saint Petersburg, Russia

**Keywords:** fusidic acid, fusidane amines, antimicrobial activity, membrane activity, molecular docking, elongation factor EF-G

## Abstract

Fusidic acid (FA) is an antibiotic with high activity against *Staphylococcus aureus*; it has been used in clinical practice since the 1960s. However, the narrow antimicrobial spectrum of FA limits its application in the treatment of bacterial infections. In this regard, this work aims both at the study of the antimicrobial effect of a number of FA amines and at the identification of their potential biological targets. In this way, FA analogues containing aliphatic and aromatic amino groups and biogenic polyamine, spermine and spermidine, moieties at the C-3 atom, were synthesized (20 examples). Pyrazinecarboxamide-substituted analogues exhibit a high antibacterial activity against *S. aureus* (MRSA) with MIC ≤ 0.25 μg/mL. Spermine and spermidine derivatives, along with activity against *S. aureus*, also inhibit the growth and reproduction of Gram-negative bacteria *Escherichia coli*, *Acinetobacter baumannii*, and *Pseudomonas aeruginosa*, and have a high fungicidal effect against *Candida albicans* and *Cryptococcus neoformans*. The study of the membrane activity demonstrated that the spermidine- and spermine-containing compounds are able to immerse into membranes and disorder the lipidsleading to a detergent effect. Moreover, spermine-based compounds are also able to form ion-permeable pores in the lipid bilayers mimicking the bacterial membranes. Using molecular docking, inhibition of the protein synthesis elongation factor EF-G was proposed, and polyamine substituents were shown to make the greatest contribution to the stability of the complexes of fusidic acid derivatives with biological targets. This suggests that the antibacterial effect of the obtained compounds may be associated with both membrane activity and inhibition of the elongation factor EF-G.

## 1. Introduction

The treatment of infectious diseases, which are among the main causes of death worldwide, is complicated by the appearance of multidrug-resistant bacteria, some strains of which appear to be resistant to all currently available antibacterial drugs. Antimicrobial drug resistance leads to increased morbidity, mortality, disability, and prolonged stay in hospital; consequently, this increases health-care costs. The World Health Organization (WHO) describes the current situation as critical and recognizes that antibiotics and other antimicrobials are becoming increasingly ineffective due to the emergence of complex forms of disease, even untreatable in some cases [1]. Since the 1980s, new antibiotics that appeared on the market have been either modifications or improvements to known molecules [2].

Over the past two decades, no new class of antibiotics has been introduced into clinical practice [3]; therefore, the search for promising antimicrobial drugs and methods for their application remains a pressing issue in chemistry and medicine [4,5,6,7]. One of the most popular strategies comprises chemical modification of drugs that have previously been approved for the treatment of bacterial infections. This approach makes it possible to obtain new biologically active compounds that have a wider scope of therapeutic action and, therefore, they are expected to overcome the drug resistance problem. For example, the chemical transformation of the lactone ring of the macrolide antibiotic erythromycin by introducing a nitrogen atom into the molecule provided the antibacterial drug azithromycin with improved pharmacokinetic properties and expanded spectrum of antimicrobial activity [8]. Another strategy is based on the application of adjuvants in order to restore obsolete antibiotics by suppressing the activity of efflux pumps and impairing membrane permeability, which promotes increased accumulation of antibiotics inside bacteria [9].

Fusidic acid (FA), a triterpenoid introduced into practice in 1962 for systemic and local therapy of staphylococcal infections, is a well-known natural antibiotic [10]. The clinical significance of FA is due to its effective distribution in various tissues, low toxicity and allergic reactions, and the absence of cross-resistance with other antimicrobial drugs [11]. However, since this compound has a rather limited spectrum of antibiotic activity, the synthesis of new FA analogues with a wider spectrum of antimicrobial activity and a long period of resistance development is of particular interest.

As shown elsewhere, the understanding of the biochemical mechanisms of the interaction of natural compounds with biological targets makes a great contribution to drug development. For instance, FA is a specific ligand to the elongation factor G (EF-G), a translational GTPase that acts on several stages of protein synthesis [12,13]. Fusidic acid interacts with EF-G-GDP, associated with a ribosome and inhibits the translocation of the growing polypeptide; it also recycles the ribosomal subunits upon reaching the stop codon in mRNA. Despite the similarity to the mammalian elongation factor (EF-2), bacterial EF-G and protein synthesis are less complex, which makes EF-G a selective target for antibiotics. Moreover, a significant place in the inventory of biologically active molecules, including those exhibiting an antibacterial effect, is occupied by nitrogen-containing derivatives [14]. Previously, it was shown that the synthesis of N-substituted analogues is a promising trend in the chemical modifications of fusidic acid [15,16,17,18].

In continuation of our previous research on the synthesis and biological activity of novel fusidane triterpenoid analogues [19,20,21,22], the present work is aimed both at the study of the antimicrobial effect of a number of FA amines and at the identification of their potential biological targets, including cell membranes. For this purpose, FA derivatives containing linear, aromatic, and heterocyclic amino groups or polyamine and pyrazinecarboxamide moieties at the C-3 atom of the molecule were synthesized. The antibacterial effect of the compounds against the ESKAPE (*Escherichia coli* (*E. coli*), *Staphylococcus aureus (MRSA)* (*S. aureus*), *Klebsiella pneumoniae* (*K. pneumoniae*), *Acinetobacter baumannii* (*A. baumannii*) *and Pseudomonas aeruginosa* (*P. aeruginosa*)) pathogens and fungi (*Candida albicans* (*C. albicans*), *Cryptococcus neoformans* (*C. neoformans*)) was evaluated, and the membrane activity of the most active methyl fusidate derivatives with polyamine and pyrazinamide substituents were studied. Using molecular docking, the interaction of the synthesized compounds with potential biological target, the prokaryotic elongation factor EF-G, was simulated to assess the prospects of FA amine analogues as inhibitors of bacterial protein synthesis.

## 2. Materials and Methods

### 2.1. General Information 

One-dimensional (^1^H and ^13^C) and two-dimensional (COSY, NOESY, HSQC, and HMBC) NMR spectra were recorded on a Bruker Avance II 500 HD Ascend spectrometer (500.17 MHz for ^1^H and 125.78 MHz for ^13^C) (Bruker, Rheinstetten, Germany). All the experiments were set up with standard Bruker pulse sequences. Chemical shifts are given in ppm relative to TMS as the internal standard. Mass spectra were measured by the MALDI TOF/TOF method on a Bruker Autoflex III spectrometer in positive ion mode. 3-(4-Hydroxy-3,5-dimethoxyphenyl)prop-2-enoic acid was used as a matrix. The elemental analyses were carried out on a Carlo Erba 1106 analyzer. The melting points were determined on PHMK 80/2617 apparatus. The reaction progress was monitored by TLC using Sorbfil plates (PTSHAF-V, Sorbopolymer, Russia), which were visualized by 10% sulfuric acid and subsequent heating at 100–120 °C for 2–3 min. Column chromatography was performed on KSK silica gel (100–200 µm, Sorbopolymer, Russia). Fusidic acid of 99.3% purity was purchased from Hangzhou Hyper Chemicals Limited. 3,11-Dioxo derivatives of fusidic acid **1** and **2** were synthesized according to the known procedure [19].

### 2.2. Synthesis of Amino Derivatives (**3**, **4**, **11**–**22**)

A mixture of a diketone (**1** or **2**) (0.5 mmol), titanium(IV) isopropoxide (0.17 mmol, 0.06 g), and primary or secondary amine (pyrazinecarboxamide, ethylenediamine, n-butylamine, pyrrolidine, benzylamine, spermidine, or spermine) (1.5 mmol) in absolute methanol (5 mL) was stirred under argon at room temperature for 3 h. Sodium borohydride (1.0 mmol, 0.04 g) was then added at −78 °C and the resulting mixture was stirred for an additional 2 h, while the temperature was increased to 22 °C. The reaction was then quenched by adding water (5 mL). Stirring was continued at room temperature for 20 min. After the filtration and washing with methanol, the organic phase was separated and concentrated in vacuo to afford the expected crude amine, which was purified by column chromatography on silica gel using the following eluents: chloroform–methanol (40:1) for compound (**3**); chloroform for compound (**4**); chloroform–methanol (2:1) for compound (**11**); (10:1) for compound (**12**); (4:1) for compounds (**13**) and (**15**); (20:1) for compounds (**14**), (**16**), and (**17**); and (40:1) for compound (**18**); and methanol for compounds (**19**–**22**).

(2Z)-2-{(3β,4α,8α,11α,14β,16β)-16-(acetyloxy)-11-hydroxy-4,8,10,14-tetramethyl-3-[(pyrazin-2-ylcarbonyl)amino]gonan-17-ylidene}-6-methylhept-5-enoic acid (**3**). White powder; 73% yield; mp 162–164 °C; [α] ^22^_D_ + 8.9° (*c* 0.805, CHCl_3_); ^1^H NMR (CDCl_3_, 500.17 MHz), *δ*: 9.42 (1H, s, H-5′), 8.80 (1H, d, *J* = 2.5 Hz, H-3′), 8.58 (1H, t, *J* = 2.5 Hz, H-4′), 5.91 (1H, t, *J* = 8.5 Hz, H-16), 5.10 (1H, d, *J* = 7.2 Hz, H-24), 4.34–4.39 (1H, m, H-11), 3.09–3.19 (1H, m, H-3), 3.04 (1H, d, *J* = 10.5 Hz, H-13), 2.22–2.42 (2H, m, H-22), 2.00–2.36 (1H, m, H-12b), 1.96–2.17 (2H, m, H-23), 1.94–2.16 (1H, m, H-15b), 1.93 (3H, s, O-C(O)CH_3_), 1.76–1.88 (1H, m, H-12a), 1.75–1.87 (1H, m, H-2b), 1.66–1.87 (1H, m, H-7b), 1.64–2.02 (2H, m, H-1), 1.61 (3H, s, H-26), 1.54 (3H, s, H-27), 1.53–1.67 (1H, m, H-6b), 1.42–1.65 (1H, m, H-2a), 1.41–1.54 (1H, m, H-5), 1.37–1.54 (1H, m, H-9), 1.28–1.39 (1H, m, H-4), 1.27 (3H, s, H-30), 1.16–1.25 (1H, m, H-15a), 1.02–1.18 (1H, m, H-7a), 0.98–1.13 (1H, m, H-6a), 0.95 (3H, s, H-19), 0.91 (3H, d, *J* = 5.0 Hz, H-28), 0.88 (3H, s, H-18); ^13^C NMR (CDCl_3_, 125.78 MHz,), *δ:* 170.5 (O-C(O)CH_3_), 168.0 (C-21), 165.9 (C-1′), 155.5 (C-17), 147.5 (C-3′), 144.4 (C-5′), 144.2 (C-2′), 142.9 (C-4′), 132.5 (C-25), 132.1 (C-20), 123.1 (C-24), 76.3 (C-3), 73.7 (C-16), 68.3 (C-11), 48.9 (C-9), 48.6 (C-14), 44.2 (C-13), 42.8 (C-5), 39.5 (C-4), 39.3 (C-8), 38.9 (C-15), 36.6 (C-10), 35.5 (C-12), 34.2 (C-1), 32.7 (C-7), 31.7 (C-2), 29.6 (C-22), 28.3 (C-23), 25.7 (C-26), 24.6 (C-30), 23.7 (C-19), 20.9 (C-6), 20.5 (O-C(O)CH_3_), 18.2 (C-18), 17.8 (C-27), 15.4 (C-28); MALDI TOF/TOF: *m/z* (I_rel_, %): 622.544 [M+H] (100), 660.473 [M+K] (75); Anal. calcd. for C_36_H_51_N_3_O_6_: C, 69.54; H, 8.27; N, 6.76 %. Found: C, 69.40; H, 8.31; N, 6.73%.

Methyl (2Z)-2-{(3β,4α,8α,11α,14β,16β)-16-(acetyloxy)-11-hydroxy-4,8,10,14- tetramethyl-3-[(pyrazin-2-ylcarbonyl)amino]gonan-17-ylidene}-6-methylhept-5-enoate (**4**). White powder; 78% yield; mp 151–153 °C; [α] ^22^_D_ +1.5° (*c* 1.057, CHCl_3_); ^1^H NMR (CDCl_3_, 500.17 MHz,), *δ:* 9.31 (1H, d, *J* = 1.5 Hz, H-5′), 8.70 (1H, d, *J* = 2.3 Hz, H-3′), 8.53 (1H, t, *J* = 2.3 Hz, H-4′), 5.77 (1H, d, *J* = 8.5 Hz, H-16), 5.01 (1H, t, *J* = 7.0 Hz, H-24), 4.23–4.31 (1H, m, H-11), 3.57 (3H, s, C(O)OCH_3_), 2.98–3.08 (1H, m, H-3), 2.96 (1H, d, *J* = 11.8 Hz, H-13), 2.26–2.48 (2H, m, H-22), 2.17–2.27 (1H, m, H-12b), 2.02–2.14 (1H, m, H-15b), 1.91–2.14 (2H, m, H-23), 1.91 (3H, s, O-C(O)CH_3_), 1.84–1.94 (1H, m, H-1b), 1.79 (1H, t, *J* = 11.5 Hz, H-12a), 1.73–1.84 (1H, m, H-2b), 1.64–1.74 (1H, m, H-7b), 1.62–1.71 (1H, m, H-1a), 1.59 (3H, s, H-26), 1.52 (3H, s, H-27), 1.51–1.64 (1H, m, H-6b), 1.48–1.61 (1H, m, H-2a), 1.42–1.52 (2H, m, H-5, H-9), 1.25 (3H, s, H-30), 1.24–1.36 (1H, m, H-4), 1.20 (1H, d, *J* = 14.0 Hz, H-15a), 1.00–1.10 (1H, m, H-7a), 0.98–1.09 (1H, m, H-6a), 0.93 (3H, s, H-19), 0.88 (3H, d, *J* = 6.5 Hz, H-28), 0.84 (3H, s, H-18); ^13^C NMR (CDCl_3_, 125.78 MHz,), *δ*: 170.9 (O-C(O)CH_3_), 170.7 (C-21), 165.7 (C-1′), 148.3 (C-17), 147.3 (C-3′), 144.3 (C-5′), 144.2 (C-2′), 142.9 (C-4′), 132.6 (C-25), 130.3 (C-20), 122.9 (C-24), 76.2 (C-3), 74.4 (C-16), 67.8 (C-11), 51.4 (C(O)OCH_3_), 48.9 (C-9), 48.6 (C-14), 43.9 (C-13), 42.7 (C-5), 39.5 (C-4), 39.3 (C-8), 38.9 (C-15), 36.5 (C-10), 35.8 (C-12), 34.1 (C-1), 32.5 (C-7), 31.3 (C-2), 28.8 (C-22), 28.2 (C-23), 25.6 (C-26), 23.9 (C-30), 23.7 (C-19), 20.9 (C-6), 20.8 (O-C(O)CH_3_), 17.6 (C-18, C-27), 15.2 (C-28); MALDI TOF/TOF: *m/z* (I_rel_, %): 636.458 [M+H] (100), 658.327 [M+Na] (40); Anal. calcd. for C_37_H_53_N_3_O_6_: C, 69.89; H, 8.40; N, 6.61 %. Found: C, 69.70; H, 8.35; N, 6.58%.

The spectral characteristics of amines (**11**, **12**) are described in Ref. [23]. The spectral characteristics of amines (**13**–**22**) are described in Ref. [20].

### 2.3. General Procedures for the Preparation of Amino Derivatives (**5**–**10**)

Diketone (**1** or **2**) (0.5 mmol), primary or secondary amine (n-butylamine, pyrrolidine, or benzylamine) (1.5 mmol), and AcOH (1.0 mmol, 0.06 g) were mixed in dry chloroform (5 mL). The mixture was stirred under argon at room temperature for 12 h and then treated with sodium triacetoxyborohydride (1.0 mmol, 0.21 g). The reaction mixture was quenched by adding 10% NaHCO_3_, and the product was extracted with chloroform. The organic extract was washed with brine and dried over MgSO_4_. The solvent was evaporated to give the crude amine, which was purified by column chromatography on silica gel using the following eluents: chloroform–methanol (4:1) for compounds (**5**), (**7**); (20:1) for compound (**6**); and (40:1) for compounds (**8**), (**9**); and chloroform for compound (**10**). 

The spectral characteristics of amines (**5**–**7**) are described in Ref. [23]. The spectral characteristics of amines (**8**–**10**) are described in Ref. [20].

### 2.4. Antimicrobial Study 

Antimicrobial, cytotoxicity, and hemolytic activity screening of the synthesized derivatives was carried out in the CO-ADD (The Community for Antimicrobial Drug Discovery) (http://www.co-add.org, accessed on 24 November 2020) and included analysis for antibacterial properties, cytotoxicity, and hemolytic activity.

Hit confirmation of active compounds by whole-cell growth inhibition assays was conducted as an 8-point dose response to determine the minimum inhibitory concentration (MIC) in duplicate (*n* = 2). The growth inhibition was assessed against those microorganisms that showed susceptibility to the compounds tested in the primary screen. The hit confirmation test included five bacteria, *Escherichia coli* (ATCC 25922), *Klebsiella pneumoniae* (ATCC 700603), *Acinetobacter baumannii* (ATCC 19606), *Pseudomonas aeruginosa* (ATCC 27853), and *Staphylococcus aureus* (ATCC 43300), and two fungi, *Candida albicans* (ATCC 90028) and *Cryptococcus neoformans* (ATCC 208821). In addition to determining MIC, active compounds were counter screened for cytotoxicity against a human embryonic kidney cell line, HEK293 (ATCC CRL-1573), by determining their CC_50_ value. The compounds were also screened for hemolysis of human red blood cells (human whole blood, strain ARCBS 5400 00150).

Samples were prepared in DMSO and water to a final testing concentration of 32 µg/mL or 20 µM (unless otherwise indicated in the data sheet) and serially diluted twofold up to 8 times. Each sample concentration was prepared in 384-well plates: non-binding surface plate (NBS; Corning 3640) for each bacterial/fungal strain, tissue-culture treated (TC-treated; Corning 3712/3764) black for mammalian cell types and polypropylene 384-well plates (PP; Corning 3657) for hemolysis assays. The final DMSO concentration was maintained to a maximum of 0.5%. All sample preparation procedures were performed using liquid-handling robots. All screening was performed as two replica (*n* = 2), with both replicas on different assay plates, but from single plating and performed in a single screening experiment (microbial incubation). In addition, two values are used as quality controls for individual plates: Z′-Factor [1 − (3* (sd(NegCtrl) + sd(PosCtrl))/(average(PosCtrl)-average(NegCtrl)))] and standard antibiotic controls at different concentrations (>MIC and <MIC). The plate passes the quality control if Z′-Factor >0.4 and standards are active and inactive at highest and lowest concentrations, respectively.

#### 2.4.1. Antibacterial Assay

Antibacterial screening was carried out by the serial dilution method. All bacteria were cultured in the cation-adjusted Mueller Hinton broth (CAMHB) at 37 °C overnight. A sample of each culture was then diluted 40-fold in fresh broth and incubated at 37 °C for 1.5–3 h. The resultant mid-log phase cultures were diluted (CFU/mL measured by OD_600_) and then added into each well containing a test compound, giving a cell density of 5 × 10^5^ CFU/mL and a total volume of 50 µL. All the plates were covered and incubated at 37 °C for 18 h without shaking. 

Inhibition of bacterial growth was determined by measuring the absorbance at 600 nm (OD_600_) with a Tecan M1000 Pro monochromator plate reader. The percentage of growth inhibition was calculated for each well, using the negative control (media only) and positive control (bacteria without inhibitors) in the same plate as references. The MIC was determined in accordance with the recommendations of the Clinical and Laboratory Standards Institute (CLSI, https://clsi.org, accessed on 24 November 2020) as the lowest concentration at which the growth was fully inhibited, which was defined as inhibition of ≥80%. In addition, the maximal percentage of growth inhibition is reported as D_Max_, indicating any compounds with partial activity. Hits were classified by MIC ≤ 16 µg/mL or MIC ≤ 10 µM in either replicate (*n* = 2 in different plates).

#### 2.4.2. Antifungal Assay

Fungi strains were cultured for 3 days on yeast extract-peptone dextrose (YPD) agar at 30 °C. A yeast suspension of 1 × 10^6^ to 5 × 10^6^ CFU/mL (as determined by OD_530_) was prepared from five colonies. The suspension was subsequently diluted and added to each well containing a test compound, giving a final cell density of fungi suspension of 2.5 × 10^3^ CFU/mL and a total volume of 50 µL. All plates were covered and incubated at 35 °C for 36 h without shaking.

Growth inhibition of *C. albicans* was determined by measuring the absorbance at 630 nm (OD_630_), while the growth inhibition of *C. neoformans* was determined by measuring the difference between the absorbances at 600 and 570 nm (OD_600–570_) after the addition of resazurin (0.001% final concentration) and incubation at 35 °C for 2 h. The absorbance was measured using a Biotek Multiflo Synergy HTX plate reader. In both cases, the percentage of growth inhibition was calculated for each well, using the negative control (media only) and positive control (fungi without inhibitors) in the same plate. MIC was found as the lowest concentration at which the growth was fully inhibited, which was defined as inhibition of ≥80% for *C. albicans* and ≥70% for *C. neoformans*. Due to a higher variance in growth and inhibition, a lower threshold was applied to the data for *C. neoformans*. In addition, the maximal percentage of growth inhibition is reported as DMax, indicating any compounds with marginal activity. Hits were classified by MIC ≤ 16 µg/mL or MIC ≤ 10 µM in either replicate (*n* = 2 in different plates).

### 2.5. Cytotoxicity Assay

HEK293 cells were counted manually in a Neubauer hemocytometer and then plated in 384-well plates containing the test compounds to give a density of 5000 cells per well in a final volume of 50 µL. DMEM supplemented with 10% FBS was used as the growth medium, and the cells were incubated together with the compounds for 20 h at 37 °C in 5% CO_2_.

The cytotoxicity (or cell viability) was measured by fluorescence, ex: 560/10 nm, em: 590/10 nm (F_560/590_), after addition of 5 µL of 25 µg/mL resazurin (2.3 µg/mL final concentration) and after incubation for further 3 h at 37 °C in 5% CO_2_. The fluorescence intensity was measured using a Tecan M1000 Pro monochromator plate reader with automatic gain control. The CC_50_ (50% cytotoxicity concentration) values were calculated by curve fitting of inhibition vs. log(concentration) using a sigmoidal dose–response function, with variable fitting values for bottom, top, and slope. In addition, the maximal percentage of cytotoxicity is reported as D_Max_, indicating any compounds with partial cytotoxicity. The curve fitting was implemented using Pipeline Pilot’s dose–response component. The results were similar to those obtained by other curve-fitting tools, such as GraphPad’s Prism and IDBS’s XlFit. Any value with > indicated samples with no activity (low D_Max_ value) or samples with CC_50_ values above the maximum tested concentration (higher D_Max_ value).

The cytotoxic samples were classified by CC_50_ ≤ 32 µg/mL or CC_50_ ≤ 10 µM in either replicate (*n* = 2 in different plates). In addition, samples were flagged as partially cytotoxic if D_Max_ ≥ 50%, even for CC_50_ > the maximum tested concentration.

### 2.6. Hemolysis Assay

Human whole blood was washed three times with 3 volumes of 0.9% NaCl and then resuspended in the same medium to a concentration of 0.5 × 10^8^ cells/mL, as determined by manual cell count in a Neubauer hemocytometer. The washed cells were then added to 384-well plates containing the test compounds to a final volume of 50 µL. After shaking on a plate shaker for 10 min, the plates were incubated for 1 h at 37 °C. After incubation, the plates were centrifuged at 1000× *g* for 10 min to pellet the cells and debris; 25 µL of the supernatant was then transferred to a polystyrene 384-well assay plate.

Hemolysis was monitored by measuring the supernatant absorbance at 405 mm (OD_405_). The absorbance was measured using a Tecan M1000 Pro monochromator plate reader. HC_10_ and HC_50_ (10% and 50% hemolysis concentrations, respectively) were calculated by curve fitting of inhibition vs. log(concentration) using a sigmoidal dose–response function with variable fitting values for top, bottom, and slope. In addition, the maximal percentage of hemolysis is reported as D_Max_, indicating any compounds with partial hemolysis. The curve fitting was implemented using Pipeline Pilot’s dose–response component. The results were similar to those obtained by other curve-fitting tools, such as GraphPad’s Prism and IDBS’s XlFit. Any value with > indicated sample with no activity (low D_Max_ value) or samples with HC_10_ values above the maximum tested concentration (higher D_Max_ value).

Hemolysis samples were classified by HC_10_ ≤ 32 µg/mL or HC_10_ ≤ 10 µM in either replicate (*n* = 2 in different plates). In addition, samples were flagged as partially hemolytic if D_Max_ ≥ 50%, even for HC_10_ > the maximum tested concentration.

### 2.7. Preparation and Quality Control of Antibiotic, Cytotoxic, and Hemolytic Standards

Colistin and vancomycin were used as positive bacterial inhibitor standards for Gram-negative and Gram-positive bacteria, respectively. Fluconazole was used as a positive fungal inhibitor standard for *C. albicans* and *C. neoformans*. Tamoxifen served as a positive cytotoxicity standard. Melittin was used as a positive hemolytic standard. Each antibiotic standard was provided in 4 concentrations, with 2 above and 2 below its MIC or CC_50_ value, and it was plated in the first 8 wells of column 23 of the 384-well NBS plates. Tamoxifen and melittin were used in 8 concentrations in 2-fold serial dilutions with 50 µg/mL highest concentration. The quality control (QC) of the assays was performed using Z′-factors calculated from the negative (media only) and positive controls (bacterial, fungal, or cell culture without inhibitor) and the standards. Plates with a Z′-factor of ≥0.4 and standards active at the highest and inactive at the lowest concentration were accepted for further data analysis [24].

### 2.8. Membrane Assay

Synthetic 1,2-diphytanoyl-*sn*-glycero-3-phosphocholine (DPhPC) and 1,2-diphytanoyl-*sn*-glycero-3-phospho-(1′-rac-glycerol) (DPhPG) were purchased from Avanti^®^ Polar Lipids (Avanti Polar Lipids, Inc., Alabaster, AL, USA). A KCl solution (0.1 M) was buffered with 5 mM HEPES, pH 7.4. Bilayer lipid membranes were prepared from DPhPC or DPhPG using a monolayer-opposition technique [25] on a 50 µm-diameter aperture in a 10 µm-thick Teflon film separating the *cis*- and *trans*-compartments of the Teflon chamber. Experiments were performed in chambers containing 0.1 M KCl, 5 mM Hepes-KOH, pH 7.4.

The test compounds (**4**, **20**, **22**) were added to the *cis*-chamber as 10 mg/mL solutions in ethanol (to mimic the physiologically relevant conditions) up to the final concentrations and the effects of the compounds on the ion permeability of the model lipid membranes were studied. Ag/AgCl electrodes with 1.5% agarose/2 M KCl bridges were used to apply the transmembrane voltage (*V*) and measure the transmembrane current (*I*). ‘Positive voltage’ refers to the case in which the *cis*-side compartment is positive with respect to the *trans*-side. All experiments were performed at room temperature.

The current was measured using an Axopatch 200B amplifier (Molecular Devices, LLC, Orlean, CA, USA) in voltage clamp mode. Data were digitized using a Digidata 1440A and analyzed using pClamp 10.0 (Molecular Devices, LLC, Orlean, CA, USA) and Origin 8.0 (OriginLab Corporation, Northampton, MA, USA). The data were acquired at a sampling frequency of 5 kHz using low-pass filtering at 200 Hz.

The threshold concentrations (*C_br_*) of the derivatives required to rupture the bilayers composed of DPhPC or DPhPG at a transmembrane voltage of 100 mV were determined. *C_br_*-values were averaged from 3 to 4 bilayers and presented as mean ± standard error (*p* ≤ 0.05).

Pore-forming ability of compound **22** was studied by applying transmembrane voltages in a range of 50–200 mV with 50 mV step and by increasing the derivative concentration in the bilayer bathing solution from 10 to 150 µg/mL.

Measurement of the membrane boundary potential. Virtually solvent-free planar lipid bilayers were made of DPhPC or DPhPG as described above. After the membrane was completely formed and stabilized and its stability was assessed by applying voltages in a range from −200 to 200 mV with a 50 mV step for 5–10 min, stock solutions of nonactin A (7 μg/mL in ethanol) were added to the bathing solution (0.1 M KCl, 5 mM HEPES, pH 7.4) in both chamber compartments to obtain a final concentration ranging from 0.1 to 1 μM.

Test derivatives (**4**, **20**, **22**) as 10 mg/mL solutions in ethanol were added to both compartments in a concentration range from 5 µg/mL to to *C_br_* in increments of 25 µg/mL.

The conductance of the bilayers was determined by measuring the membrane conductance (*G*) at a constant transmembrane voltage of 50 mV. In the subsequent calculations, the membrane conductance was assumed to be related to the membrane boundary potential (*φ_b_*), the potential drop between the aqueous solution and the membrane hydrophobic core, by the Boltzmann distribution [26]

(1) $$\frac{G_{m}}{G_{m}^{0}}=\exp(\frac{e\Delta \varphi_{b}}{kT})$$

where *G_m_* and *G_m_*^0^ are the steady-state membrane conductances induced by K^+^-nonactin in the presence and absence of test compounds, respectively; *e*, *k*, and *T* have their usual meanings.

The changes in the membrane boundary potential for the defined experimental conditions were averaged from 4 bilayers and presented as mean ± standard deviation (*p* ≤ 0.05).

Calcein release from liposomes. Small unilamellar vesicles (Ø100 nm) composed of 1,2-dioleoyl-*sn*-glycero-3-phosphocholine (DOPC) or 1,2-dioleoyl-*sn*-glycero-3-phospho-(1′-rac-glycerol) (DOPG) and loaded with the fluorescent dye calcein (35 mM) were obtained using a mini-extruder (Avanti Polar Lipids, Alabaster, AL, USA). At this concentration, calcein fluorescence inside the liposomes is self-quenched. The calcein that was not entrapped in the vesicles was removed by gel filtration with calcein-free solution (150 mM KCl, 10 mM HEPES, pH 7.4). An increase in the free calcein fluorescence in a surrounding medium is a measure of the disturbance of membrane integrity, in the absence and presence of test compounds (**4**, **20**, **22**). The control samples were not modified. Experimental samples were treated by 5 µg/mL of the compounds (**4**, **20**, **22**). Fluorescence of released calcein was determined on a Fluorat-02-Panorama spectrofluorimeter (Lumex, Saint-Petersburg, Russia) (excitation wavelength of 490 nm, emission wavelength of 520 nm). The detergent Triton X-100 (at a final concentration of 1%) was added at the end of experiments for complete disruption of liposomes (complete release of calcein from the vesicles).

The relative intensity of calcein fluorescence (*IF*, %) was calculated as:

(2) $$IF=\frac{I-I_{0}}{I_{\max}/0.9-I_{0}}\cdot 100\%$$

where *I* and *I*_0_ are the calcein fluorescence intensities in the presence and absence of test compounds, respectively, and *I_max_* is the maximal fluorescence after treatment of liposomes with Triton X-100 (a factor of 0.9 was introduced to account for sample dilution by Triton X-100).

### 2.9. Molecular Docking Studies

Molecular docking was carried out for 24 compounds (**1**–**22**, FA, FA methyl ester). The geometric parameters of the compounds were optimized using the GAUSSIAN 09 program [27] and the semi-empirical PM6 method. As a biological target, we considered the crystal structure of the elongation factor (4v5f) [28] with a resolution of 3.60 Å deposited in the Cambridge Structural Database (www.rcsb.org, accessed on 6 June 2022).

Molecular docking was performed using the Gold Suite software [29]. The docking procedure involved retrieval of structure of the native ligand from initial complex, after which the ligand binding cavity of the target protein was analyzed. The active site used for the docking was defined as all protein residues within 12 Å of the location of the native ligand (FA) in the ligand binding domain of the elongation factor (4v5f). Further determination of the ligand binding pocket was performed using the Ligsite algorithm [30,31] that is implemented in Gold Suite software.

The genetic algorithm (GA) was used as a conformational search algorithm. Genetic algorithm parameters were selected by default: number of docking runs for each ligand—10, maximum number of iterations—2000, population size—100, maximum number of operations—100,000, number of islands—5, niche size—2, mutation frequency—95, crossover frequency—95, migration frequency—10. 

All solutions were scored according to Piecewise Linear Potential (CHEMPLP) fitness function [29,32].

In the course of molecular docking, the crystal structure of target protein and the complete conformational mobility of the studied ligands was taken into account. The analysis and visualization of the receptor–ligand complex were carried out using the Hermes program (Gold Suite) [29].

## 3. Results

### 3.1. Chemistry

**Scheme 1:**
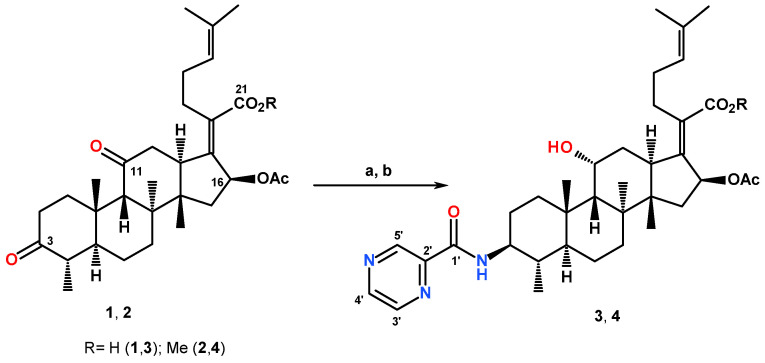
Reagents and conditions: **a**. pyrazinecarboxamide, MeOH, Ti(i-PrO)_4_, 22 °C, 3 h; **b**. NaBH_4_, −78 °C→22 °C, 2 h (73% for **3**, 78% for **4**).

Fusidic acid derivatives were synthesized by reductive amination [33] of the corresponding 3,11-dioxo analogues (**1**, **2**). The reaction of **1** and **2** with pyrazinecarboxamide in the presence of Ti(Oi-Pr)_4_ as a catalyst followed by reduction in the reaction mixture with NaBH_4_ provided amines **3** and **4** in 73 and 78% yields, respectively (Scheme 1). The reaction proceeded with high chemo- and stereoselectivity at the C-3 atom, while the keto group at C-11 was not involved in amination and was reduced to a hydroxyl group. As followed from the NOESY spectra, the reductive amination was accompanied by the inversion of the asymmetric C-3 atom configuration from α to β, while the C-11 carbon retained the α-configuration of the native molecule [23].

**Figure 1:**
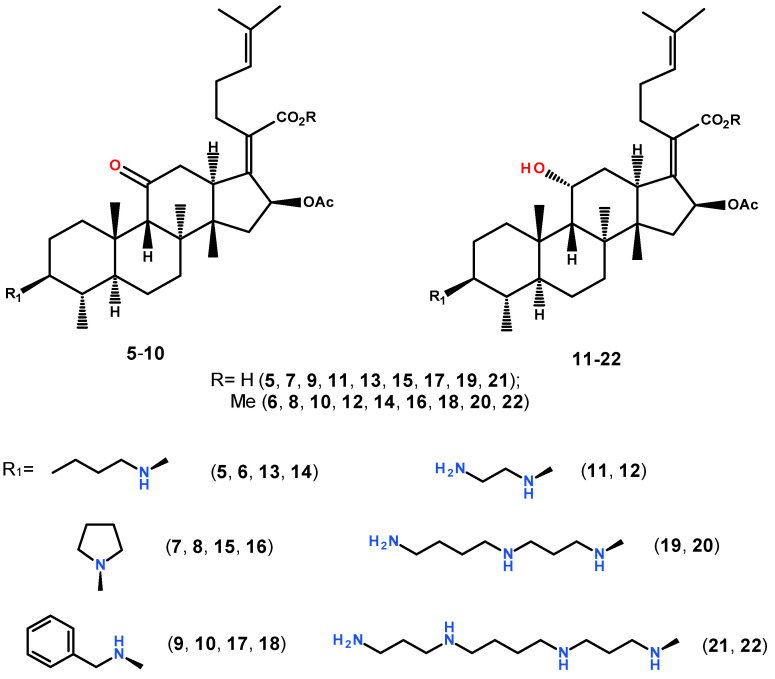
Structures of 11-oxo-3β amino and 11α-hydroxy-3β amino derivatives of fusidane triterpenoids.

Compounds **5**–**22** were obtained in our previous works [20,23] via the reaction of 3,11-dioxo analogues **1** and **2** with n-butylamine, pyrrolidine, and benzylamine or with polyamines—1,2-diaminoethane, spermine, and spermidine (Figure 1). NaBH(OAc)_3_ (compounds **5**–**10**) and NaBH_4_ (compounds **11**–**22**) were used as reducing agents.

### 3.2. Biological Evaluation 

#### 3.2.1. In Vitro Antibacterial and Antifungal Activity

Twenty-two amino derivatives of fusidic acid were studied for their in vitro antibacterial activity against five bacterial strains, *E. coli*, *S. aureus, K. pneumoniae*, *A. baumannii*, and *P. aeruginosa*, and two fungal strains, *C. albicans* and *C. neoformans*. 

In the course of primary screening, compounds **1**, **3**, **4**, **7**, **9**, **12**, **14**, and **18** showed antimicrobial activity against *S. aureus* (MRSA). Among them, compounds **12** and **18** additionally inhibited the growth of fungi *C. neoformans*. FA analogues **19**–**22** with spermidine and spermine substituents exhibited a wide spectrum of antibacterial activity against strains of *S. aureus*, *E. coli*, *P. aeruginosa*, *A. baumannii*, *C. albicans*, and *C. neoformans* (Table S1, Supplementary Material). For active compounds, the minimum inhibitory concentrations (MICs) were determined, and cytotoxic and hemolytic activities were studied as well.

Pyrazinecarboxamide-substituted FA and its C-21 methyl ester (**3** and **4**) possessed the highest antimicrobial activities, inhibiting the growth and reproduction of Gram-positive bacteria *S. aureus* (MRSA) at a concentration of 0.25 µg/mL, which is comparable to the activity of the native fusidic acid. The FA analogues with pyrrolidine (**7**) and benzylamine (**9**) moieties showed a lower antibacterial effect against this pathogen (MIC = 8.0 µg/mL), while the methyl fusidate with butylamine substituent **14** inhibited the growth of *S. aureus* with minimal activity at a concentration of 32.0 µg/mL. Moreover, the hemolytic activity was significantly lower for compounds **3**, **4**, **7**, **9**, and **14** than for FA; however, the toxicity of derivatives **1**, **4**, and **14** was two-times higher than the toxicity of the natural antibiotic (Table 1).

Methyl fusidate analogues with ethylenediamine (**12**) and benzylamine (**18**) moieties exhibited moderate antibacterial activity against *S. aureus* (MIC 32.0 and 16.0 µg/mL, respectively) and fungicidal effect, inhibiting the growth and reproduction of *C. neoformans* (MIC 16.0 and 32 µg/mL, respectively). Furthermore, they were characterized by a two-fold increase in toxicity compared with FA (Table 2).

The introduction of polyamine moieties into the fusidane triterpenoid molecule (compounds **19–22**) led to an expansion of the spectrum of antimicrobial activity, namely, the appearance of a positive effect on Gram-negative pathogens (Table 3), which may be due not only to the interaction of the compounds with the bacterial elongation factor EF-G, similarly to FA [34], but also to changes in the membrane activity. As follows from Table 3, compounds **19–22** exhibited the greatest antimicrobial effect against the bacterial strain *S. aureus* (MIC 2.0–8.0 µg/mL) and the fungal culture *C. neoformans* (MIC 1.0–2.0 µg/mL). The methyl fusidate derivative with a spermine moiety (**22**) had the widest range of antimicrobial activity. This compound influences the viability of *S. aureus* (MIC 2.0 µg/mL), *E. coli* (MIC 32.0 µg/mL), *P. aeruginosa* (MIC 32.0 µg/mL), *A. baumannii* (MIC 32.0 µg/mL), *C. albicans* (MIC 32.0 µg/mL), and *C. neoformans* (MIC 1.0 µg/mL). The FA derivative with spermidine substituent **19** inhibited the growth of *S. aureus* (MIC 32 µg/mL) and *C. neoformans* (MIC 8.0 µg/mL) and proved to be the least toxic and hemolytically active compared to other polyamine analogues. The transformation of this molecule to methyl ester to give compound **20** elevates the antimicrobial and antifungal activity; however, this is also accompanied by an increase in the toxicity and hemolytic effect (Table 3).

#### 3.2.2. Membrane Activity Assay

To support the hypothesis that the bacterial and fungal membranes are the primary targets of antimicrobial action of synthesized compounds, we tested the effects of methyl fusidate derivatives with pyrazinamide (**4**), spermidine (**20**), and spermine (**22**) moieties on the model lipid membranes of various compositions. The main criterion for selecting compounds for testing on lipid bilayers mimicking the target membranes was the highest efficacy against the selected pathogens. In particular, C-21 methyl ester of pyrazinecarboxamide-substituted FA, compound **4**, was the most effective against *S. aureus* compared to the parent molecule (**1**), FA analogs with pyrrolidine (**7**) and benzylamine (**9**) fragments, and the methylfusidate with butylamine substituent (**14**) (Table 1). Compound **20** was more effective against *S. aureus* and *C. neoformans* than the synthesized spermidine derivative (**19**) (Table 3). Similarly, compound **22** was characterized by lower MICs against *S. aureus* and *C. neoformans* than the other synthesized spermine derivative (**21**) (Table 3). The membranes composed of electrically neutral phosphatidylcholine (PC) and negatively charged phosphatidylglycerol (PG) species were used. Table S2 in the Supplementary Material demonstrates the chemical structures of the lipids. It should be noted that the PC is an abundant lipid of mammalian and cryptococcal cell membranes, while PG is the major phospholipid of bacteria [35,36,37,38,39,40]. 

Table 4 summarizes the parameters referring to the effects of compounds **4**, **20**, and **22** on the properties of planar lipid bilayers and permeability of small unilamellar vesicles. The threshold concentrations of the test compounds required to rupture the bilayers (*C_br_*) are presented to characterize the action of the compounds on the model membranes of different compositions. The addition of derivatives **4**, **20**, and **22** up to the concentrations of 225, 275, and 250 μg/mL, respectively, to uncharged membranes composed of diphytanoylphosphatidylcholine (DPhPC) did not lead to an increase in the ion permeability of the lipid bilayers. The subsequent increase in the concentration of the compounds caused a violation of the electrical stability of lipid bilayers and their subsequent destruction. When neutral DPhPC was replaced by negatively charged diphytanoylphosphatidylglycerol (DPhPG), the threshold concentration *C_br_* corresponding to membrane rupture decreased 1.4–1.9-fold (Table 4). The decrease in the threshold concentrations of the tested compounds in DPhPG membranes compared to DPhPC bilayers may be attributable to the cationic nature of the tested polyamines [41,42,43].

**Figure 2:**
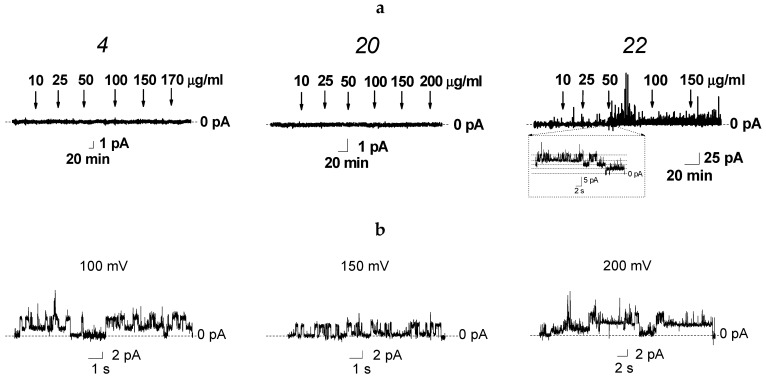
(**a**) The examples of time tracks of the transmembrane current (*I*) induced by compounds **4**, **20**, and **22** in DPhPG bilayers. The bilayers were bathed in 0.1 M KCl, pH 7.4. The transmembrane voltage was equal to 100 mV. The moments of addition of compounds **4**, **20**, and **22** are indicated by arrows. Above the arrows are the concentrations of the agents in the membrane bathing solution. *Inset:* Stepwise current fluctuations induced by compound **22**. (**b**) The examples of current fluctuations corresponding to openings and closures of single transient pores induced by compound **22** in DPhPG bilayers at different transmembrane voltages (100, 150, and 200 mV). Membranes were bathed in 0.1 M KCl, 5 mM HEPES, pH 7.4. The derivative concentration in bilayer bathing solution was equal to 50 µg/mL.

The left and middle panels in Figure 2a show that an introduction of compounds **4** and **20** into membrane bathing solution up to 170 and 200 μg/mL, respectively, does not affect ionic permeability of lipid bilayers composed of DPhPG. These data demonstrate that **4** and **20** are not able to form transient ion-permeable pores in the DPhPG membranes at this concentration range. A further increase in the concentration of the compounds leads to the disintegration of the lipid bilayers without pore formation, wherein compound **22** in a concentration range of 35–40 μg/mL to *C_br_* was able to produce stepwise current fluctuations in DPhPG bilayers (Figure 2a, right panel). This fact might indicate the induction of ion-permeable transmembrane pores in negatively charged bilayers by **22**. The probability of ion-permeable pore formation by derivative **22** and the characteristics of the pores do not significantly depend on the value of the applied transmembrane voltage (Figure 2b); the amplitude of the observed stepwise current fluctuations varies in a range of 20–160 pS. 

**Figure 3:**
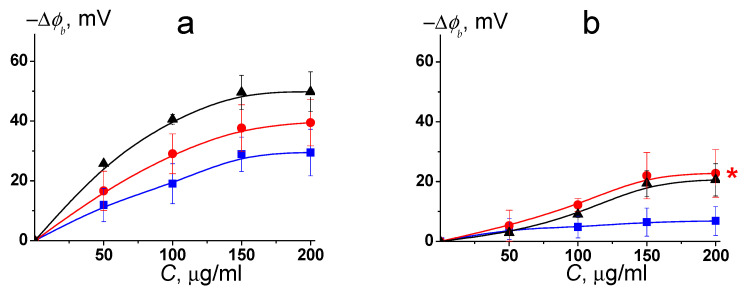
Decrease in the boundary potential of the membrane (–Δφ*_b_*) vs. the concentration (*C*) of compounds **4** (*black curves*), **20** (*blue curves*), and **22** (*red curves*) in the bilayer bathing solution. The membranes were made of DPhPC (**a**) and DPhPG (**b**), bathed in 0.1 M KCl (5 mM HEPES, pH 7.4), and modified by K^+^-ionofore, nonactin. The transmembrane voltage was 50 mV. * The absolute Δφ*_b_*-values in the presence of compound **22** might be overestimated due to the increase in transmembrane current due to the pore-forming ability of the derivative.

We also studied the transmembrane distribution of the electrical potential in the presence of compounds **4**, **20**, and **22**. Figure 3 illustrates the dependence of the decrease in the membrane boundary potential (–Δφ*_b_*) of DPhPC and DPhPG bilayers on the concentration of the compounds. Table 4 shows the maximum changes in the φ*_b_* at an infinitely high concentration of the compound (–Δ*φ_b_*(max)). It was found that under physiological conditions (0.1 M KCl, pH 7.4), compounds **4**, **20**, and **22** cause a decrease in the boundary potential of DPhPC membranes by approximately 50, 30, and 40 mV, respectively (Figure 3a, Table 4). Replacement of DPhPC by DPhPG leads to a 2–2.5-fold reduction in the potential-modifying efficiency of the tested compounds (Figure 3b, Table 4). The membrane boundary potential, a voltage drop across membrane/water interface, consists of the diffuse part of the electric double layer (surface potential, φ*_s_*) and the internal dipole component, φ_d_ [44,45]. A negative change in the boundary potential indicates that the absolute value of the decrease in the dipole component exceeds the increase in the surface potential of neutral membranes upon the adsorption of polyamine cations. The preferential sorption of polyamine cations on negatively charged membranes should lead to the partial neutralization of the surface charge of the bilayers, but the total change in the boundary potential remains negative, also indicating the dominance of the dipole component over the surface one. This might be attributed to relatively high dipole moments of the tested molecules, whose values are equal to 3.84, 4.24, and 3.94 D for **4**, **20**, and **22,** respectively (the μ-values were predicted by the PM6 method), and their substantial immersion into the lipid bilayers due to hydrophobic fusidane triterpenoid fragment.

**Figure 4:**
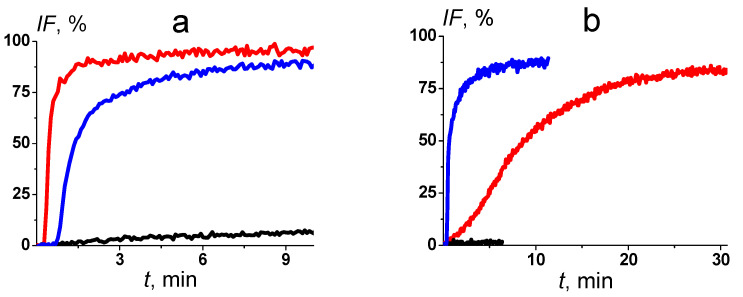
Time dependence of the relative fluorescence of calcein (*IF*, %) leaked from DOPC (**a**) and DOPG (**b**) vesicles induced by addition of compounds **4** (black curves), **20** (blue curves), and **22** (red curves) up to 5 μg/mL.

To assess the damage induced by immersion of the test agents into the lipid bilayers, we examined the release of the fluorescent dye, calcein, from small unilamellar liposomes of different compositions. Figure 4 shows the time dependences of calcein leakage (*IF*) from vesicles composed of neutral dioleoylphosphatidylcholine (DOPC) (a) and negatively charged dioleoylphosphatidylglycerol (DOPG) (b) upon a successive increase in the concentrations of compounds **4**, **20**, and **22**. The maximal leakages induced by the tested compounds are presented in Table 4. The huge effects (almost 100%) of spermidine and spermine derivatives **20** and **22** on the permeability of both types of liposomes for fluorescent dye indicate that these compounds have pronounced disordering action on the membrane lipids. Probably, their intercalation into the lipid bilayer is accompanied by an increase in the area per lipid molecule, a decrease in the packing density of membrane-forming lipids and, therefore, an increase in the membrane permeability for calcein. These data confirm the ability of polyamine derivatives to strongly interact with lipopolysaccharides, ensuing permeabilization of the outer membrane [9]. Compound **4** demonstrated a low effect on the permeability of DOPC and DOPG liposomes for the dye (*IF* did not exceed 7 and 3%, respectively) (Figure 4); hence, a significant disordering effect cannot be considered for this compound.

The kinetics of the increase in calcein release from DOPC and DOPG liposomes after addition of compounds **20** and **22** are described by a single exponential function with a characteristic time, *τ*. Significant slowdown in leakage kinetics from DOPG liposomes in the presence of compound **22** might be related to the ability of **22** to induce ion-permeable pores in PG membranes (Figure 2).

### 3.3. Docking Studies

The possibility of FA amino derivatives to inhibit the protein synthesis via the EF-G factor was studied. For this purpose, the affinity of the synthesized compounds **1**–**22** for elongation factor EF-G was assessed using molecular docking. According to cryoelectron microscopy and X-ray diffraction data, the binding region of the fusidic acid molecule to the elongation factor is located between domains G, II, and III, and in the corresponding receptor–ligand complex, the acyloxy group of ring D of FA interacts with THR-84 (switch II, domain G), while the cyclic part is located between domains II and III [28].

**Figure 5:**
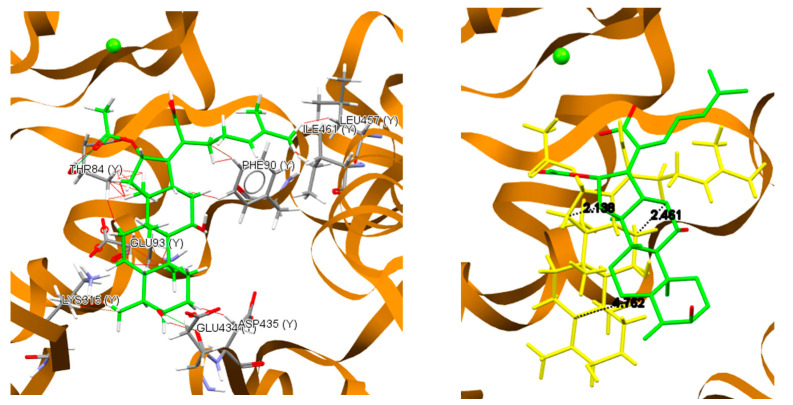
Comparison of X-ray diffraction data (green color) and the result of molecular docking of fusidic acid (yellow color).

Comparison of the XRD results [28] and the molecular docking data shows a certain change in the position of the optimized fusidic acid molecule from the position of the native molecule in the ligand-binding part of the target protein. Rings A and B are displaced towards the domain II region by 4.76 Å and ring C deviates by 2.46 Å (Figure 5). In molecular modeling of the receptor–ligand complex, the fusidic acid molecule is coordinated via two hydrogen bonds that are located between the acyloxy group at the C-20 atom and the hydroxyl group of THR84 and also between the hydroxyl group at C-3 and the carbonyl group of GLU434. The van der Waals interactions of the molecule occur with six amino acid residues (Table S4, Supplementary material). The observed contacts of the fusidic acid molecule with THR84 and PHE90 in the simulated complex correspond to the experimental data [28,46], which allowed us to consider the difference between the docking and XRD results (RMS = 0.116) to be acceptable.

Compounds **1** and **2** with carbonyl groups in C-3 and C-11 positions have lower values for the evaluation function than FA molecule (Table S3, Supplementary Material). In a simulated receptor–ligand complex, these compounds are located at a distance from the G domain, orienting the ring towards domain III and the side chain towards domain II (Figure S1).

**Figure 6:**
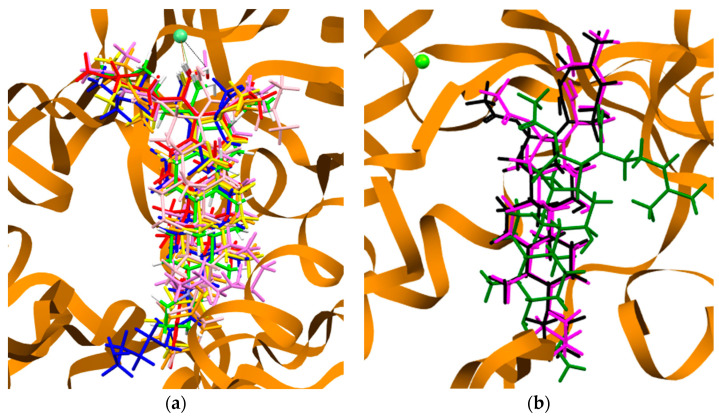
Complex of EF-G with ligands: (**a**) **3** (red), **5** (yellow), **7** (light green), **9** (pink), **17** (orange), **19** (blue), and **21** (violet); (**b**) **11** (green), **13** (magenta), and **15** (black).

The amine-substituted FA analogues **3**–**22** are oriented in the ligand-binding part of the elongation factor similarly to the natural molecule (Figure 6). The presence of amino groups in ring A leads to an increase in the affinity of the parent compound for the target protein in silico due to the predominant increase in the proportion of energy contributions from nonpolar interactions (PLP nonpolar) (Table S3). The carboxyl group of ring A of modified compounds is oriented towards domain G. Depending on the nature of the substituent at C-3, the molecules are displaced towards the magnesium ion to different extents. In particular, the side-chain carboxyl groups of compounds **3**, **5**, **7**, **9**, **17**, **19**, and **21** are coordinated to the metal ion (Figure 6a), as evidenced by the respective weighting factors (plp. part. metal) (Table S3). However, adducts **11**, **13**, and **15** are significantly shifted from G to domain III region (r ˃ 4 Å) (Figure 6b), thus directing the acyloxy and carboxyl groups by analogy with the substrate molecule, which is explained by the absence of the Bayer strain in ring C and a small bulk of substituents in ring A. In this case, the side chain of ligands **3**, **4**, **5**, **7**, **17**, **19**, and **21** is oriented towards domain II.

Compared to amino derivatives of FA with a free carboxyl group **3**, **5**, **7**, **9**, **11**, **13**, **15**, **17**, **19**, and **21**, methyl esters **4**, **6**, **8**, **10**, **12**, **14**, **16**, **18**, **20**, and **22** have lower values of the fitness parameter (Table S3). The methyl group in the carboxyl moiety blocks one of the active sites of the initial ligands; as a result, upon the formation of the virtual receptor–ligand complex, the compounds move away from the magnesium ion and the acyloxy group becomes directed towards the switch II of domain G. The reduction in the carbonyl group at C-11 in molecules **5**–**10** to hydroxyl group relieves the Bayer torsional strain of ring C, which, depending on the nature of the substituent of ring A, affects the fitness parameter of the obtained compounds **13**–**18** in different ways (Table S3).

The results of in vitro studies presented in Table S1 indicate a greater efficacy of 11-keto acids **5**, **7**, **9**, and **15** against *S. aureus* compared to esters **6**, **8**, **10**, and **16**, which is consistent with the results of molecular docking. However, in the series of compounds with a hydroxyl group at C-11, the highest activity towards the biological target is mainly inherent in ester molecules (with the exception of ligands with pyrrolidine (**16** and **15**) and spermine (**22** and **21**) substituents in ring A). It is assumed that the observed phenomena are largely due to the processes of interaction of the ligands with the membrane, the mechanisms of which are currently being studied.

**Figure 7:**
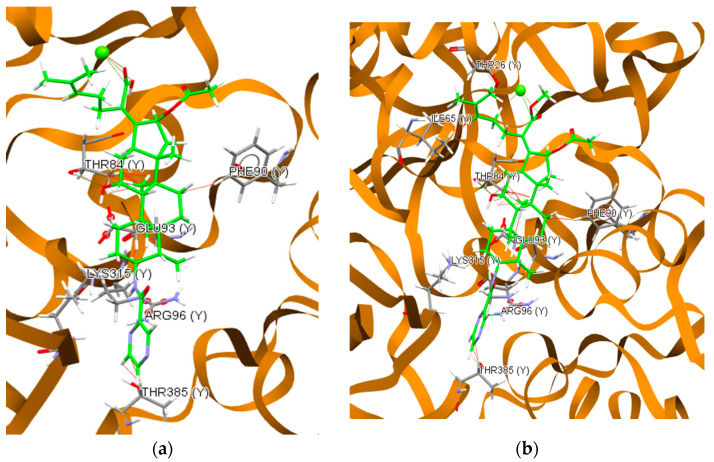
Complex of elongation factor (Ef-G) with ligands **3** (**a**) and **4** (**b**).

Products **3** and **4** with a pyrazinamide substituent in ring A are among the most active in the interaction with *S. aureus* (Table 2). In the simulation of the receptor–ligand complex, derivatives **3** and **4** are shifted from the position of fusidic acid to the G domain region by r = 2.97 Å and 3.12 Å, respectively. As a result, the functional groups at C-20 of these molecules are coordinated to a metal ion present in the receptor (plp part. Metal) (Table S3, Figure 7). The obtained weight coefficients for the formation of hydrogen bonds in each case are close in magnitude to those of fusidic acid (Table S4) and may indirectly indicate the stability of the complexes obtained in silico. It is assumed that the observed phenomena are due to the structure and polar nature of the substituent of ring A. The side chains of the considered molecules **3** and **4** are oriented towards domain II.

Comparison of the results of molecular docking for ligands **5**, **6**, **11**, **19**, **20**, **21**, and **22** demonstrates an increase in the weight coefficients of hydrogen bond formation energy (PLP part.hbond) with increasing number of nitrogen atoms in the substituent in ring A (Table S3).

In the studied series of compounds, ligands with spermine substituents (**21** and **22**), according to the results of in vitro study, exhibit the highest total antimicrobial activity (Table 3 and Table S1). In the course of molecular docking, these fusidic acid derivatives show the highest values of the evaluation function (Table S3). The presence of the spermine moiety, unlike the other substituents under consideration, in the C-3 position of these molecules provides the maximum increase in the weight coefficients for the formation of hydrogen bonds and non-polar interactions in the simulated receptor–ligand complex.

The interactions of the ligands **1**, **3**, **4**, **7**, **9**, **14**, **12**, **19**, **20**, **21**, **22**, and FuA with THR84 or PHE90 amino acid moieties indicate the stability of the resulting receptor–ligand complexes, which is in accordance with the conclusions made earlier [28,46] and with our in vitro data summarized in Table 2.

Thus, in the series of fusidic acid derivatives, ligands with a spermine substituent in position C-3 demonstrate the highest level of steric complementarity to the ligand-binding part of the elongation factor. In the simulated receptor–ligand complexes, the acid molecules were superior to the ester molecules in the affinity level for the considered target protein. This can be attributed to the blocking of the active site, the carboxyl function at the C-21 atom. The study showed the greatest contribution of substituents, with long aliphatic chain and an increased number of amino groups in the substituent of ring A to the energy of hydrogen bond formation and hydrophobic interactions with EF-G. Moreover, it was shown that the absence of torsional tension in ring C promotes the displacement of polycyclic molecules to the hydrophobic region III. However, the level of affinity of all the studied ligands for the receptor is mainly determined by the nature of the substituent in position C-3.

The obtained results provide insight into the possible coordination of the studied compounds in the ligand-binding part of the target protein and can be used in the design of new potentially biologically active fusidic acid derivatives.

## 4. Discussion

Fusidic acid is a natural antibiotic belonging to the class of 29-nor-protostane triterpenes with high biological activity against Gram-positive *S. aureus* bacteria, including strains possessing cross-resistance with other antibiotics [47,48]. However, the narrow spectrum of antibacterial activity of FA, which is active only against staphylococci, limits its use in medical practice [49]. Therefore, the design and synthesis of new FA analogues with an extended spectrum of antimicrobial activity may be one of the tactics to overcome this limitation.

A promising direction of FA synthetic modifications is the introduction of various amine fragments into the molecule, since amines are the most important components of naturally occurring and synthetic antibiotics [50]. Previously, a number of amino derivatives of 3-ketofusidic acid were synthesized by the reaction of the latter with primary aliphatic linear and branched polyamines (for example, *N,N*-bis(3-aminopropyl)-1,3-propanediamine, *N,N*-bis(3-aminopropyl)propane-1,3-diamine) in the presence of acetic acid and NaBH(OAc)_3_, and functionalization of the carboxyl group of tetrahydrofusidic acid with aliphatic long-chain amines to obtain C21 amides was carried out [51,52]. The 3-amino-substituted fusidic acid was found to be inactive against Gram-positive and Gram-negative bacteria and fungi, while some C21 amides with polyamine fragments, including spermine and spermidine, exhibited a broad spectrum of antimicrobial activity. In the present work, we synthesized a number of FA analogues containing aliphatic and aromatic amino groups, as well as spermine and spermidine, at the C-3 atom via reductive amination of ketones catalyzed with (i-PrO)_4_Ti [53]. The use of a titanium catalyst allowed for an increase in the yields of the target compounds, as well as to avoid the formation of products of excessive alkylation [54].

Extended antimicrobial screening, in which the MICs of active compounds were determined, revealed that derivatives with a pyrazinecarboxamide fragment (**3** and **4**) have the highest antibacterial effect against *S. aureus* (MRSA). The activity of these compounds is comparable to the effect of the native molecule, FA, and causes the complete death of the pathogen at a concentration of 0.25 µg/mL. The antibacterial effect of the obtained compounds may be due to the inhibition of bacterial cell protein synthesis by binding to the elongation factor EF-G, similar to the action of the original antibiotic, FA [55,56], which corresponds to the results of molecular docking that we carried out for all the synthesized compounds. In the simulated protein–ligand interaction, a high level of affinity in biologically active molecules **1**, **3**, **4**, **7**, **9**, **14**, **12**, **19**, **20**, **21**, and **22** to the EF-G was found. The assumption of the formation of stable complexes with the elongation factor also corresponds to the results of molecular docking for other fusidic acid derivatives (lactone, halides, 3,4-dehydrogenated analogues, azide, esters, amines, etc.) [34,57,58], which requires further experimental confirmation.

A wide spectrum of antimicrobial activity of FA analogues substituted with polyamines (**19**–**22**) could be due to the ability to effectively interact with both the elongation factor and membranes. Recently, the bacterial membrane has attracted considerable interest as a nonspecific target for antibacterial agents. Progress in the study of membrane-active compounds has shown that those that are capable of disrupting the integrity of the membrane, as a rule, are lipophilic and positively charged molecules [59]. For example, positively charged amino groups are able to electrostatically interact with negatively charged lipids on the surface of bacterial membranes, while the incorporation of bulky hydrophobic substituents associated with terminal amino groups in the lipid layer leads to an increase in membrane permeability, depolarization with a violation of the transmembrane proton gradient, and loss of cell wall rigidity, integrity, and further lysis [59]. These properties give such compounds the ability to act as chemosensitizers to enhance the effectiveness of other antibacterial agents. The literature reports that simple unsubstituted polyamines, spermine and spermidine, can act synergistically with a number of antibiotics [60,61]. Moreover, recent studies of natural or synthetic products containing polyamines have shown that molecules, such as squalamine [62], motuporamine MOTUN44 [63], ianthelliformisamine [64], 6-bromoindolglyoxamide [65], and polyaminoisoprenoid NV716 [66,67], possess both an antimicrobial effect and the ability to enhance the activity of antibiotics by increasing membrane permeability and/or membrane depolarization [68].

Taking into account the literature data, in order to confirm the hypothesis that the bacteria and fungi membranes are the primary targets of the antimicrobial action of the synthesized compounds, we studied the effect of analogues **4**, **20**, and **22** on different model systems of lipid membranes. The studies showed that compounds **20** and **22** with polyamine moieties are able to immerse into PC- and PG-containing bilayers and disorder the membrane lipids. The pronounced observed effects of agents **20** and **22** on the liposome permeability for fluorescent marker may indicate that their antimicrobial action is associated with the damage of staphylococcal and cryptococcal membranes, and can also explain the relatively high toxicity of compound **20** (Table 3). Moreover, the derivative of spermine **22** is also capable of inducing the ion-permeable pores in the bilayers mimicking the bacterial membranes. The pyrazinamide-containing fusidic acid analogue **4** is characterized by a significantly weaker detergent effect that does not allow for linking its biological action to the disruption of bacterial membranes. 

As a result, we identified promising FA analogues that have a pronounced antimicrobial effect, which may be due to the interaction with both the bacterial cell membrane and the elongation factor. Thus, further investigation of biochemistry with experimental identification of targets as well as detailed modeling and in vivo studies are required to confirm the mechanism of the antimicrobial action of the obtained compounds.

## 5. Conclusions

We synthesized 20 amino derivatives of fusidic acid containing butylamine, pyrrolidine, benzylamine, pyrazinamide, and polyamine (ethylenediamine, spermine, or spermidine) substituents. The results of the antimicrobial screening of the products showed that the introduction of a pyrazinecarboxamide molecule into the fusidane structure (compounds **3** and **4**) provides a high antibacterial effect against Gram-positive bacteria *S. aureus* (MRSA); this effect is comparable to the effect of a natural antibiotic. However, it has the advantage of lower hemolytic activity. Functionalization of the triterpenoid molecule by long-chain amines, spermidine (compounds **19**, **20**) and spermine (compounds **21**, **22**), leads to an expansion of the spectrum of antimicrobial action; these analogues inhibit the viability of both Gram-positive (*S. aureus* (MRSA)) and Gram-negative bacterial pathogens (*E. coli*, *A. baumannii*, *P. aeruginosa*), and also exhibit a high fungicidal effect against two fungal strains, *C. albicans* and *C. neoformans*. The study of the cytotoxic and hemolytic activity of the synthesized compounds showed that the transformation of the carboxyl group of amino derivatives into an ester group leads to an increase in the toxicity against the HEK293 human embryonic kidney cell line. The methyl fusidate derivative with a spermidine moiety (**20**) showed the greatest cytotoxic and hemolytic effects.

Using the model lipid membranes, we showed that the fusidic acid methyl esters with spermine and spermidine polyamine radicals are able to deeply immerse into the lipid bilayers and disorder the membrane lipids leading to a significant detergent effect. Moreover, the spermine-substituted compound is also capable of inducing the ion-permeable pores in the bilayers mimicking the bacterial membranes.

The molecular docking studies established the greatest contribution of polyamine substituents to the stability of complexes of fusidic acid derivatives with the elongation factor EF-G. Thus, the antibacterial effect of the compounds with polyamine substituents can be due to both membrane-active properties and the ability to effectively interact with the elongation factor, which allows us to consider these derivatives as a starting point for the development of new antibiotic agents with a wide spectrum of action.

## Figures and Tables

**Table 1:** Antibacterial, cytotoxic, and hemolytic activity of compounds **1**, **3**, **4**, **7**, **9**, **14,** and fusidic acid.

Compound	Antibacterial Activity		Cytotoxic Activity		Hemolytic Activity	
	*Staphylococcus aureus*		HEK293			
	MIC *, µg/mL	*D_max_* **, %	CC_50_ ***, µg/mL	*D_max_*, %	HC_50_ *^4^, µg/mL	*D_max_*, %
**1**	8.0 8.0	98.9 99.0	>32 >32	26.9 38.8	>32 >32	7.3 10.6
**3**	**≤0.25** **≤0.25**	99.0 99.7	>32 >32	42.1 27.9	>32 >32	2.2 1.4
**4**	**≤0.25** ≤**0.25**	100.5 98.6	24 >32	94.8 96.8	>32 >32	3.9 3.9
**7**	8.0 8.0	97.6 105.7	>32 >32	32.9 58.5	>32 >32	2.3 2.6
**9**	8.0 8.0	101.1 99.5	>32 >32	20.9 19.3	>32 >32	3.7 2.3
**14**	32 32	95.4 98.1	26.0 23.8	89.4 88.0	>32 >32	3.4 3.6
**Fusidic acid**	≤0.25 ≤0.25	97.7 99.2	>32 >32	25.1 41.1	>32 >32	10.0 9.7
**Vancomycin**	1	100	-	-	-	-
**Tamoxifen**	-	-	9	50	-	-
**Melittin**	-	-	-	-	8.5	50

* MIC (the minimum inhibitory concentration) is the lowest concentration at which complete inhibition of bacteria is observed. The compounds were categorized as active at MIC ≤16 µg/mL in any replicate (*n* = 2 in different plates). ** *D_max_* is the highest fraction of inhibition. *** CC_50_ is the concentration of compounds at which 50% cell death is observed in the human embryonic kidney cell line HEK293 (ATCC CRL-1573). *^4^ HC_50_ is the concentration of compound resulting in 50% hemolysis of erythrocytes.

**Table 2:** Antibacterial, cytotoxic, and hemolytic activity of compounds **12**, **18**, and fusidic acid.

Compound	Antibacterial Activity		Antifungal Activity		Cytotoxic Activity		Hemolytic Activity	
	*Staphylococcus aureus*		*Cryptococcus neoformans*		HEK293			
	MIC, µg/ml	*D_max_,* %	MIC, µg/ml	*D_max_,* %	CC_50_, µg/ml	*D_max_*, %	HC_50_, µg/ml	*D_max_*, %
**12**	32 32	87.6 92.2	**16** **16**	110.0 113.5	7.77 13.2	98.6 98.2	>32 >32	34.8 27.8
**18**	**16** **16**	100.3 98.1	32 32	73.4 73.6	17.8 17.5	94.6 96.5	>32 >32	17.0 8.1
**Fusidic acid**	≤0.25 ≤0.25	97.7 99.2	>32 >32	−6.3 −12.0	>32 >32	25.1 41.1	>32 >32	10.0 9.7
**Vancomycin**	1	100	-	-	-	-		
**Fluconazole**	-	-	8	100	-	-	-	-
**Tamoxifen**	-	-	-	-	9	50	-	-
**Melittin**	-	-	-	-	-	-	8.5	50

**Table 3:** Antibacterial, cytotoxic, and hemolytic activity of compounds **19–22** and fusidic acid.

Comp.	Antibacterial Activity								Antifungal Activity				Cytotoxic Activity		Hemolytic Activity	
	*S. aureus*		*E. coli*		*P. aeruginosa*		*A. baumannii*		*C. albicans*		*C. neoformans*		HEK293			
	MIC, µg/mL	*D_max_,* %	MIC, µg/mL	*D_max_,* %	MIC, µg/mL	*D_max_,* %	MIC, µg/mL	*D_max_,* %	MIC, µg/mL	*D_max_,* %	MIC, µg/mL	*D_max_,* %	CC_50_, µg/mL	*D_max_,* %	HC_50_, µg/mL	*D_max_,* %
**19**	32 32	104.8 104.5	>32 >32	8.4 14.2	>32 >32	26.7 35.8	>32 >32	26.4 25.6	>32 >32	6.5 11.8	8.0 8.0	110.5 109.3	>32 >32	29.9 16.7	>32 >32	5.5 5.3
**20**	4.0 8.0	102.5 99.2	32 32	95.2 95.3	>32 >32	4.1 8.9	32 32	97.7 88.3	32 32	100.2 100.9	**2.0** **2.0**	84.0 85.7	15.4 14.9	100.4 99.6	27.1 27.8	61.8 60.5
**21**	4.0 4.0	101.7 98.6	32 32	97.2 97.5	32 32	97.0 98.0	32 32	100.2 98.9	32 32	100.6 101.0	**2.0** **2.0**	81.0 84.3	24.2 >32	80.2 95.9	>32 >32	8.7 6.4
**22**	**2.0** **2.0**	102.9 98.6	32 32	95.1 96.1	32 32	97.2 97.3	32 32	100.3 99.6	32 32	100.8 101.5	**1.0** **2.0**	86.8 87.4	18.8 18.1	98.6 99.2	>32 >32	15.1 11.3
**Fusidic acid**	≤0.25 ≤0.25	97.7 99.2	>32 >32	10.71 15.8	>32 >32	24.0 24.6	>32 >32	26.8 35.6	>32 >32	2.5 6.1	>32 >32	−6.3 −12.0	>32 >32	25.1 41.1	>32 >32	10.0 9.7
**Vancomycin**	1	100														
**Colistin**	-	-	0.125	100	0.25	100	0.25	100	-	-	-	-	-	-	-	-
**Fluconazole**	-	-	-	-	-	-	-	-	0.125	100	8	100	-	-	-	-
**Tamoxifen**	-	-	-	-	-	-	-	-	-	-	-	-	9	50	-	-
**Melittin**	-	-	-	-	-	-	-	-	-	-	-	-	-	-	8.5	50

**Table 4:** Parameters characterizing the effects of compounds **4**, **20**, and **22** * on the model lipid membranes of various compositions.

	DPhPC		DPhPG		DOPC		DOPG	
Comp. *	C_br_, μg/mL	−Δφ_b_(max), mV	C_br_, μg/mL	−Δφ_b_(max), mV	IF_max_, %	τ, min	IF_max_, %	τ, min
**4**	225 ± 15	49 ± 7	163 ± 11	21 ± 5	7 ± 3	–	3 ± 2	–
**20**	275 ± 25	29 ± 7	190 ± 13	11 ± 4	88 ± 3	0.5 ± 0.2	87 ± 4	1.0 ± 0.1
**22**	250 ± 11	40 ± 8	135 ± 10	23 ± 8	95 ± 2	0.7 ± 0.2	83 ± 3	9.8 ± 0.4

*C_br_* is the threshold concentration of compounds in the membrane bathing solutions (0.1 M KCl, pH 7.4) required to rupture the bilayer. −Δ*φ_b_*(max) is the maximum decrease in the membrane boundary potential. *IF_max_* is the maximum release of fluorescent marker from lipid vesicles after the addition of 5 μg/mL of a test compound into liposome suspension. *τ* is the characteristic parameter of the time dependence of marker release in the presence of test compounds. * Compound **4**—Methyl (2Z)-2-{(3β,4α,8α,11α,14β,16β)-16-(acetyloxy)-11-hydroxy-4,8,10,14-tetramethyl-3-[(pyrazin-2-ylcarbonyl)amino]gonan-17-ylidene}-6-methylhept-5-enoate; compound **20**—Methyl (2Z)-2-[(3β,4α,8α,11α,14β,16β)-16-(acetyloxy)-3-({3-[(4aminobutyl)amino]propyl} amino)-11-hydroxy-4,8,10,14- tetramethylgonan-17-ylidene]-6-methylhept-5-enoate; compound **22**—Methyl (2Z)-2-[(3β,4α,8α,11α,14β,16β)-16-(acetyloxy)-3-{[3-({4-[(3-aminopropyl)amino]butyl}amino)propyl]amino}-11-hydroxy-4,8,10,14-tetramethylgonan-17-ylidene]-6-methylhept-5-enoate.

## Data Availability

The data used to support the findings of this study are available from the corresponding author upon request.

## Supplementary Materials

The following supporting information can be downloaded at: https://www.mdpi.com/article/10.3390/membranes13030309/s1, Table S1: % Inhibition of the growth of microorganisms by compounds **1**—**22** and fusidic acid at a concentration of 32 μg/mL; Table S2: The chemical structures of the lipids and the total electrical charge at neutral pH; Table S3: The values of the CHEMPLP evaluation function for compounds **1**–**22** and the energy contributions to the formation of the ligand elongation factor (EF-G) complex; Table S4: Hydrogen bonds and nonpolar interactions of the studied ligands with amino acid residues of EF-G (4v5f) receptor; Figure S1: Location of ligands **1** (a) and **2** (b) in the ligand-binding cavity of the elongation factor (EF-G) according to the results of molecular docking; Spectral characteristics of compounds.
