# Supplementary material for: 3-Amino-Substituted Analogues of Fusidic Acid as Membrane-Active Antibacterial Compounds
Source: Membranes (Basel). 2023 Mar 7;13(3):309. doi: 10.3390/membranes13030309 (PMC10056636; doi:10.3390/membranes13030309)

3-Aminosubstituted Analogs of Fusidic Acid as Membrane Active Antibacterial Compounds

Elena V. Salimova[a], Oleg S. Mozgovoj[a], Svetlana S. Efimova[b], Olga S. Ostroumova[b], Lyudmila V. Parfenova[a,]*

[a] *Institute of Petrochemistry and Catalysis, Ufa Federal Research Center, Russian Academy of Sciences, 141 Prospect Oktyabrya, 450075, Ufa, Russian Federation*

[b] *Institute of Cytology of Russian Academy of Sciences, 4 Tikhoretsky prospect, 194064, Saint Petersburg, Russian Federation*

*Corresponding author. E-mail address: luda_parfenova@ipc-ras.ru

Supporting Information

Page Number

Table S1 3

Table S2 4

Table S3 5

Table S4 7

Figure S1 11

^{1}H, ^{13}C NMR of compound **3** 12

^{1}H, ^{13}C NMR of compound **4** 13

^{1}H, ^{13}C NMR of compound **5** 14

^{1}H, ^{13}C NMR of compound **6** 15

^{1}H, ^{13}C NMR of compound **7** 16

^{1}H, ^{13}C NMR of compound **8** 17

^{1}H, ^{13}C NMR of compound **9** 18

^{1}H, ^{13}C NMR of compound **10** 19

^{1}H, ^{13}C NMR of compound **11** 20
^{1}H, ^{13}C NMR of compound **12** 21
^{1}H, ^{13}C NMR of compound **13** 22
^{1}H, ^{13}C NMR of compound **14** 23
^{1}H, ^{13}C NMR of compound **15** 24
^{1}H, ^{13}C NMR of compound **16** 25
^{1}H, ^{13}C NMR of compound **17** 26
^{1}H, ^{13}C NMR of compound **18** 27
^{1}H, ^{13}C NMR of compound **19** 28
^{1}H, ^{13}C NMR of compound **20** 29
^{1}H, ^{13}C NMR of compound **21** 30
^{1}H, ^{13}C NMR of compound **22** 31

Table S1. % Inhibition of the growth of microorganisms by compounds **1** - **22** and fusidic acid at a concentration of 32 µg/ml

Comp.	Gram-positive	Gram-negative				Fungus	
	S. aureus	*E.coli*	*K. pneumoniae*	*A. baumannii*	*P. aeruginosa*	*C. albicans*	*C. neoformans*
**1**	98.00	22.44	10.98	23.79	18.82	7.05	-17.63
**2**	14.56	14.59	14.72	24.64	12.27	4.73	-20.22
**3**	96.23	0.07	6.52	12.73	0.97	1.97	-6.98
**4**	98.66	2.49	10.37	24.36	12.04	2.35	11.64
**5**	14.84	25.02	13.81	2.4	7.51	5.14	15.52
**6**	6.53	21.36	0.72	-11.8	17.72	-5.18	-9.21
**7**	96.8	21.9	8.93	7.73	5.41	1.45	21.77
**8**	-8.97	21.66	2.39	1.31	7.75	2.66	35.41
**9**	101.9	4.99	4.24	10.72	12.42	1.57	8.38
**10**	-2.4	2.49	10.76	8.66	19.82	10.46	11.18
**11**	4.85	9.46	10.76	12.75	14.91	2.42	-6.7
**12**	90.52	25.73	6.89	24.3	5.88	14.3	108.5
**13**	37.57	26.63	11.77	11.14	15.45	1.5	1.74
**14**	100.1	8.01	10.15	12.4	10.52	2.51	6.7
**15**	13.76	28.06	8.37	15.71	6.5	5.17	4.06
**16**	-3.94	10.51	8.16	11.18	0.44	5.58	8.59
**17**	30.88	4.9	-4.39	16.26	2.6	2.11	9.84
**18**	100.1	0.31	7.45	19.3	15.66	5.12	70.79
**19**	97.52	17.08	4.92	25.27	19.89	14.72	111.5
**20**	99.13	102.2	23.03	99.56	7.68	99.4	69.53
**21**	101.8	105.2	52.88	80.34	99.59	100.5	89.21
**22**	99.81	102.5	20.3	100	98.52	98.66	75.6
**FA**	97.86	21.57	14.15	45.16	22.72	1.08	2.11
**FAMe**	27.78	21.26	15.56	16.32	19.11	5.54	-18.50

Table S2. The chemical structures of the lipids and the total electrical charge at neutral pH

DPhPG (charge **-1**)	DPhPC (charge **0**)
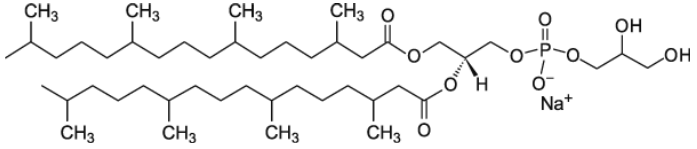	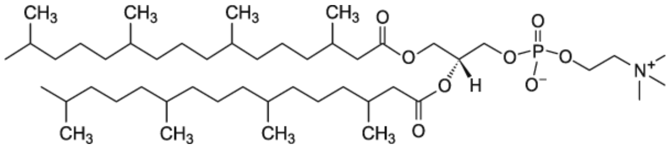
4ME 16:0 PG	4ME 16:0 PC
1,2-diphytanoyl-*sn*-glycero-3-phospho-(1'-rac-glycerol)	1,2-diphytanoyl-*sn*-glycero-3-phosphocholine
DOPG (charge **-1**)	DOPC (charge **0**)
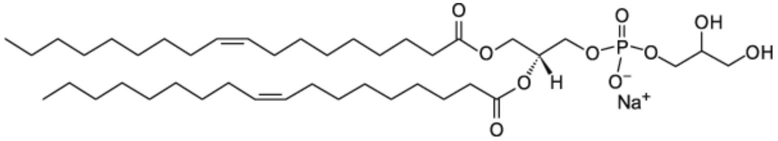	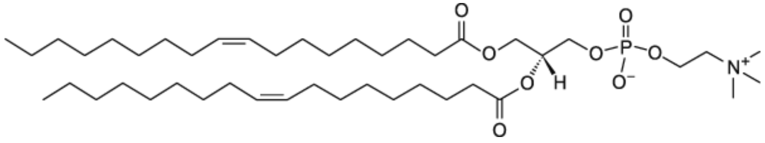
18:1 (Δ9-Cis) PG	18:1 (Δ9-Cis) PC
1,2-dioleoyl-*sn*-glycero-3-phospho-(1'-rac-glycerol)	1,2-dioleoyl-*sn*-glycero-3-phosphocholine

Table S3. The values of the CHEMPLP evaluation function for compounds **1-22** and the energy contributions to the formation of the ligand − elongation factor (EF-G) complex

Comp.	PLP fitness	PLP.Che. Hbond	PLP.Che. Int.Cor.	PLP.Che. Metal	PLP.PLP	PLP.lig clash	PLP.ligand tors	PLP.part. buried	PLP.part hbond	PLP.part metal	PLP.part nonpolar	PLP.part Repulsiv
**1**	44.30	0.0000	0.0147	0.0000	-45.8237	0.0000	0.7692	-1.7764	-0.9474	0.0000	-45.4491	0.4223
**2**	46.67	0.0000	0.0187	0.0000	-47.2540	0.0000	0.3024	1.4264	-0.2156	0.0000	-49.1742	0.2017
**3**	66.57	1.0000	0.2505	1.9865	-50.3395	0.0000	0.9298	0.9516	-2.0000	-2.0000	-49.3774	0.5578
**4**	65.05	0.8287	0.2523	0.9951	-56.4952	0.0000	1.4505	-1.6402	-2.0000	-1.0000	-54.6463	0.5651
**5**	67.12	1.6640	0.4849	1.6971	-53.5551	0.0000	1.0453	-5.5522	-2.0000	-1.3201	-47.2029	0.0201
**6**	63.21	0.0000	0.5631	0.0000	-65.9110	0.0000	1.6331	-6.0615	-1.6794	0.0000	-59.1003	0.1360
**7**	60.47	1.0000	7.4827	0.9399	-57.0175	0.0000	6.3339	-4.8322	-1.0000	-0.9682	-52.4695	0.4134
**8**	58.30	0.0000	7.4784	0.0000	-60.9769	0.0000	5.0755	-6.4675	-1.0000	0.0000	-54.3736	0.2956
**9**	72.56	1.0000	0.1571	1.9644	-59.7504	0.0000	1.0672	-4.1325	-1.0000	-2.0000	-54.2011	0.1639
**10**	69.54	0.0000	0.2903	0.0000	-71.6164	0.0000	1.1854	-6.0928	-2.0000	0.0000	-64.7952	0.0598
**11**	57.88	0.0000	0.1402	0.0000	-58.1469	0.0000	0.2038	-7.1450	-2.6546	0.0000	-49.1018	0.0000
**12**	54.73	0.0000	0.2635	0.0000	-56.0629	0.0000	0.7985	-6.9302	-2.6096	0.0000	-47.2677	0.0204
**13**	61.79	0.0000	0.4095	0.0000	-64.2877	0.0000	1.4558	-6.8917	-1.0000	0.0000	-58.1397	0.0000
**14**	60.91	1.0000	0.3885	0.0000	-60.7114	0.0000	1.5930	-6.6858	-1.6195	0.0000	-54.1481	0.0000
**15**	59.31	0.0000	7.5331	0.0000	-63.6056	0.0000	5.9123	-7.1555	-1.0000	0.0000	-55.8932	0.1020
**16**	58.64	0.0000	7.5478	0.0000	-61.4999	0.0000	5.2037	-5.8149	-1.0000	0.0000	-54.5568	0.0831
**17**	74.74	0.8255	0.6233	1.9984	-69.8309	0.0000	0.5885	2.3512	-3.1813	-2.0000	-59.6783	0.0275
**18**	65.97	0.0000	0.1418	0.0000	-68.9146	0.0000	1.5450	-6.4230	-1.0000	0.0000	-62.7202	0.0000
**19**	73.38	2.3575	0.5060	1.9265	-60.0806	0.0209	2.9093	1.9952	-3.4888	-1.9645	-56.0053	0.0475
**20**	67.19	2.0000	1.1790	0.0000	-63.0644	0.0000	1.5263	-6.6890	-3.7243	0.0000	-53.1229	0.1357
**21**	81.08	1.5839	1.7659	1.9986	-67.4610	0.0000	2.4462	1.5552	-4.0415	-2.0000	-65.2763	0.0833
**22**	80.79	0.9204	1.8137	0.0000	-80.6285	0.0000	2.2026	-7.5170	-4.6310	0.0000	-71.5583	0.0000
**FA**	52.56	2.0000	0.1157	0.0000	-47.5557	0.0000	0.5549	-.4.5715	-2.0000	0.0000	-39.9640	0.1447
**FAMe**	51.13	2.0000	0.0434	0.0000	-46.2303	0.0000	0.5731	-5.0103	-2.0000	0.0000	-38.8090	0.1416

PLP – Piecewise leaner potential, responsible for modeling steric complementarity between protein and ligand (weights);

PLP Chem. Hbond - weight coefficient of the energy of hydrogen interactions, calculated by the empirical function ChemScore ;
PLP Chem int. cor. (PLP Chem intenal correction) – weight coefficients of internal ligand correction energy (ChemScore parameter);
PLP Chem Prot. En. (PLP Chem Protein Energy) – protein energy term to penalize clashes when using flexible sidechains;
PLP S.Bar – penalty coefficient of activation of a water molecule in the process of conformational search algorithm;
PLP lig. Clash (PLP ligand clash) – penalty coefficient of intramolecular interaction of the ligand, and intermolecular interaction of two or more low molecular weight ligands;
PLP ligand torsion – weight coefficient of the energy of the torsion interaction of the ligand with the protein;
PLP part. Buried – weight coefficient of Van der Waals energy interactions of polar ligand groups with the hydrophobic part of the protein;
PLP part. Hbond - weight coefficient of the hydrogen bond energy calculated by the Piecewise leaner potential function;
PLP part nonpolar – weight coefficient of the energy of nonpolar interactions between a ligand and a protein;
PLP part repulsive – weight coefficient of the energy of repulsive interactions between a ligand and a protein.

Table S4. Hydrogen bonds and nonpolar interactions of the studied ligands with amino acid residues of EF-G (4v5f) receptor

Ligands	PLP fitness	Hydrogen bonding interaction	Nonbonded interaction				
			Alkyl	Pi-alkyl	Pi-Pi- -T-stacked	Pi-S	Halogen
	EF-G						
**1**	44.30	-	LYS315<br>ILE316<br>ILE65<br>ALA67<br>ARG96	PHE90	-	-	-
**2**	46.67	-	ARG96<br>ILE461<br>GLU434	-	-	-	-
**3**	66.57	THR84<br>ARG96	GLU93<br>LYS315<br>THR384<br>THR385	PHE90	-	-	-
**4**	65.05	THR84<br>ARG96	GLU93<br>THR26<br>ILE65<br>GLU93<br>LYS315<br>THR384	PHE90	-	-	-
**5**	67.12	GLU434	THR26<br>THR84<br>LYS315<br>GLU434<br>LYS316	PHE90	-	-	-
**6**	63.21	ARG96	LEU457<br>ILE461<br>GLU93<br>ARG96<br>ASP435<br>PRO436<br>THR385<br>ILE316	PHE90	-	-	-
**7**	60.47	THR84	VAL88<br>THR84<br>ILE65<br>GLU93<br>LYS315	PHE90	-	-	-
**8**	58.30	ARG96	ILE316<br>ILE461<br>GLU93	PHE90	-	-	-

			ARG96<br>ARG465<br>ASP435<br>LYS315<br>PRO436<br>THR385				
**9**	72.56	GLU434	ILE65<br>ILE316<br>GLU434<br>THR84<br>VAL88	PHE90	-	-	-
**10**	69.54	ARG96	LEU457<br>ILE461<br>GLU93<br>ARG96<br>ASP435<br>PRO436<br>ILE316	PHE90	-	-	-
**11**	57.88	ARG96	ILE461<br>ILE462<br>LEU431<br>ARG465<br>GLU93<br>ARG96<br>THR385<br>LYS315	-	-	-	-
**12**	54.73	ARG96	THR84<br>LEU457<br>ILE461<br>GLU93<br>ARG96<br>LYS315<br>PHE314	PHE90	-	-	-
**13**	61.79	ARG96	ARG457<br>ILE461<br>GLU93<br>LYS315<br>ASP435<br>ARG96<br>ILE316	PHE90	-	-	-
**14**	60.91	ASP435<br>ARG96	LEU457<br>ASP435<br>ILE316<br>LYS315<br>ARG96	PHE90	-	-	-
**15**	59.31	ARG96	LEU457<br>LEU461	PHE90	-	-	-

			GLU93<br>ARG96<br>ASP435<br>THR385				
**16**	58.64	ARG96	LEU457<br>THR84<br>GLU93<br>ARG96<br>ASP435<br>LYS315<br>PRO436<br>THR385<br>ILE316	PHE90	-	-	-
**17**	74.74	ARG96	THR26<br>ILE65<br>THR84<br>ARG96<br>PRO436<br>THR385<br>ILE316	PHE90	-	-	-
**18**	65.97	ARG96	ILE461<br>GLU93<br>ILE316	-	-	-	-
**19**	73.38	GLU434<br>ARG96<br>THR84	ILE65<br>GLU93<br>LYS315<br>ILE316<br>GLU434<br>THR84	PHE90	-	-	-
**20**	67.19	THR84<br>GLU434<br>ILE316	GLU93<br>LYS315<br>ARG96<br>ILE316<br>THR84<br>GLU434	-	-	-	-
**21**	81.08	ARG96<br>GLU434<br>ARG465	THR26<br>ILE65<br>THR84<br>GLU93<br>GLU434<br>ASP435<br>ARG465	PHE90	-	-	-
**22**	80.79	ARG96<br>GLU434<br>ARG324	ILE461<br>GLU93<br>ARG96<br>ASP435	PHE90	-	-	-

			GLU434<br>PRO436<br>ILE316<br>MET317				
**FA**	52.56	THR84<br>GLU434	LYS315<br>ASP435<br>LEU457<br>ILE461<br>GLU434<br>THR84	PHE90	-	-	-
**FAMe**	51.13	GLU434	THR84<br>GLU93<br>LYS315<br>ASP435<br>GLU434	PHE90	-	-	-

a)

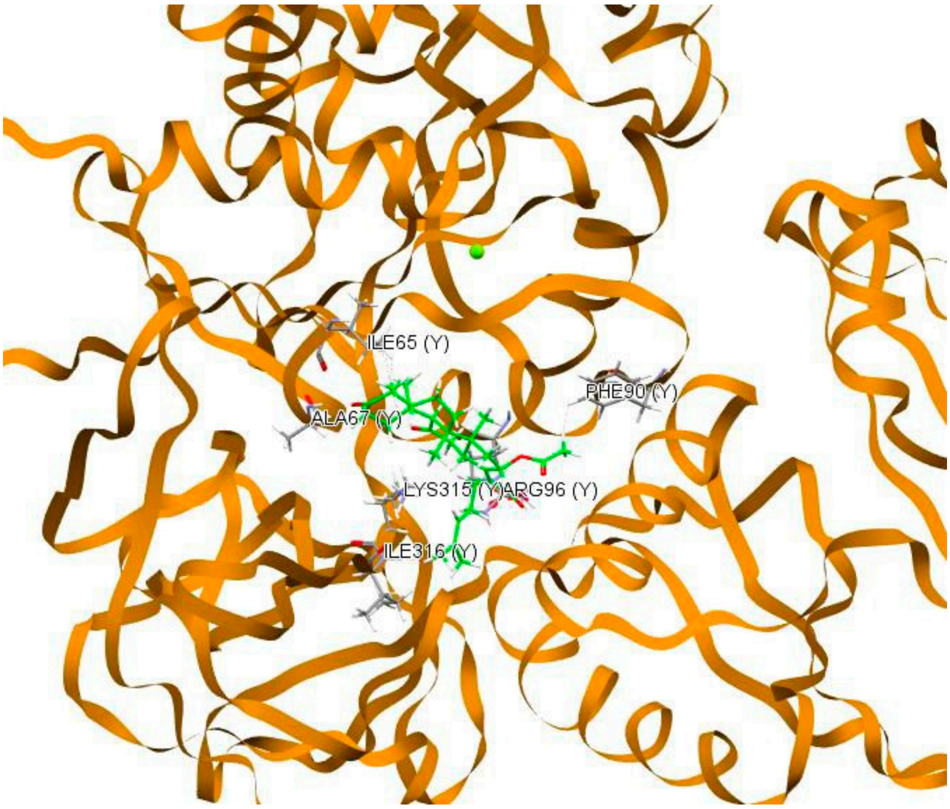

b)

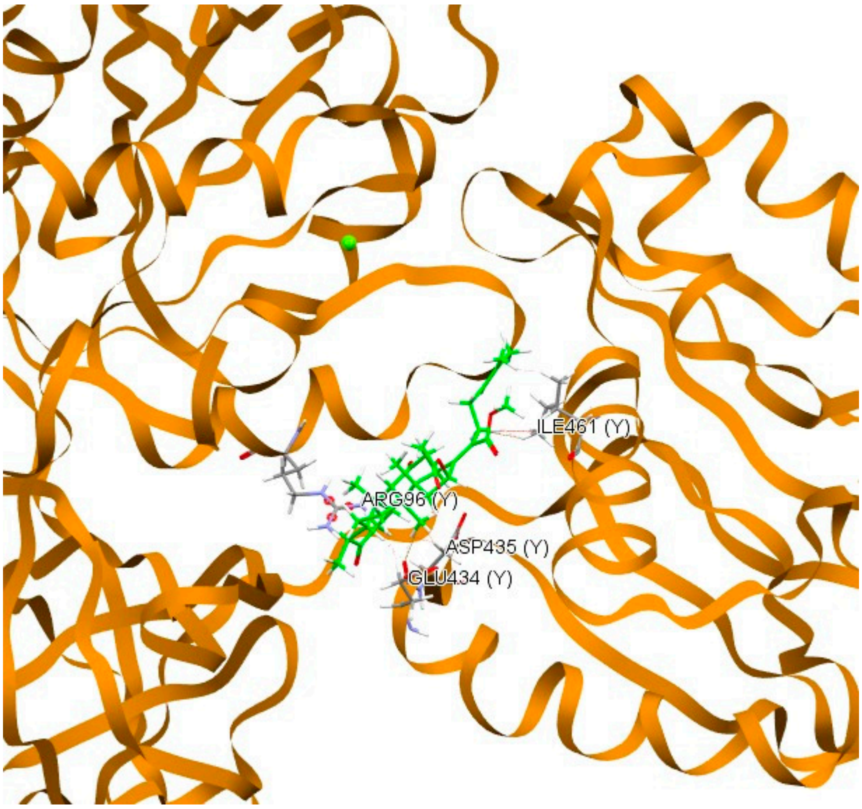

Figure S1. Location of ligands **1** (a) and **2** (b) in the ligand-binding cavity of the elongation factor (EF-G) according to the results of molecular docking

Compound **3:** ^{1}H NMR, 500.17 MHz, $CDCl_3$

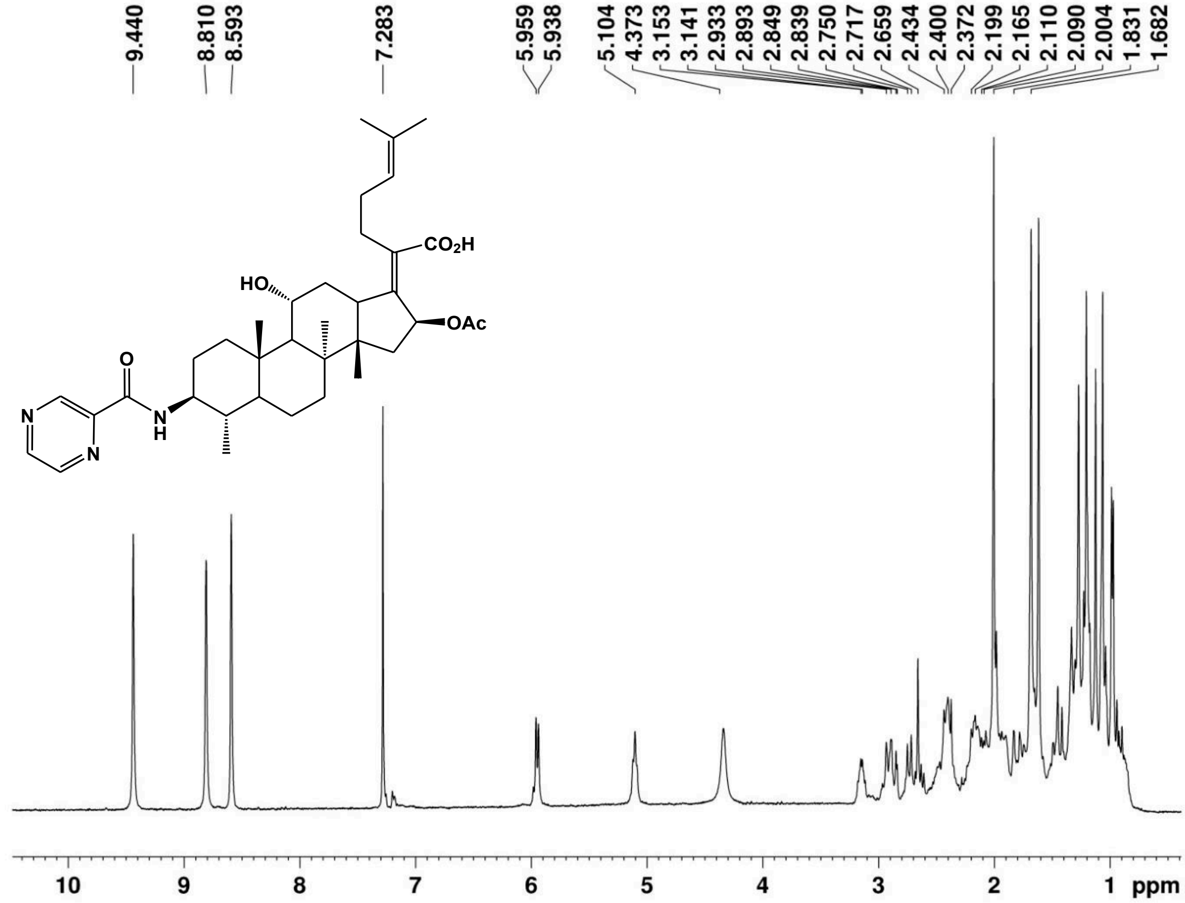

^{13}C NMR, 125.78 MHz, $CDCl_3$

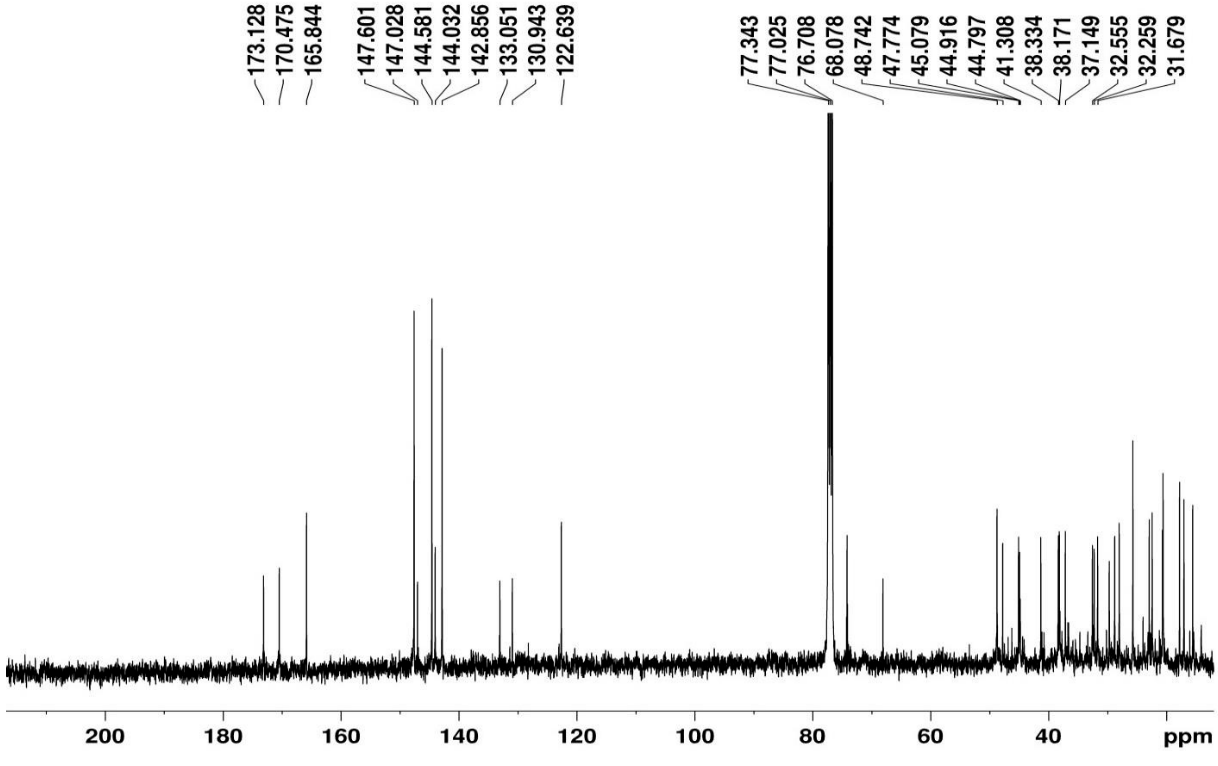

Compound **4:** ^{1}H NMR, 500.17 MHz, $CDCl_3$

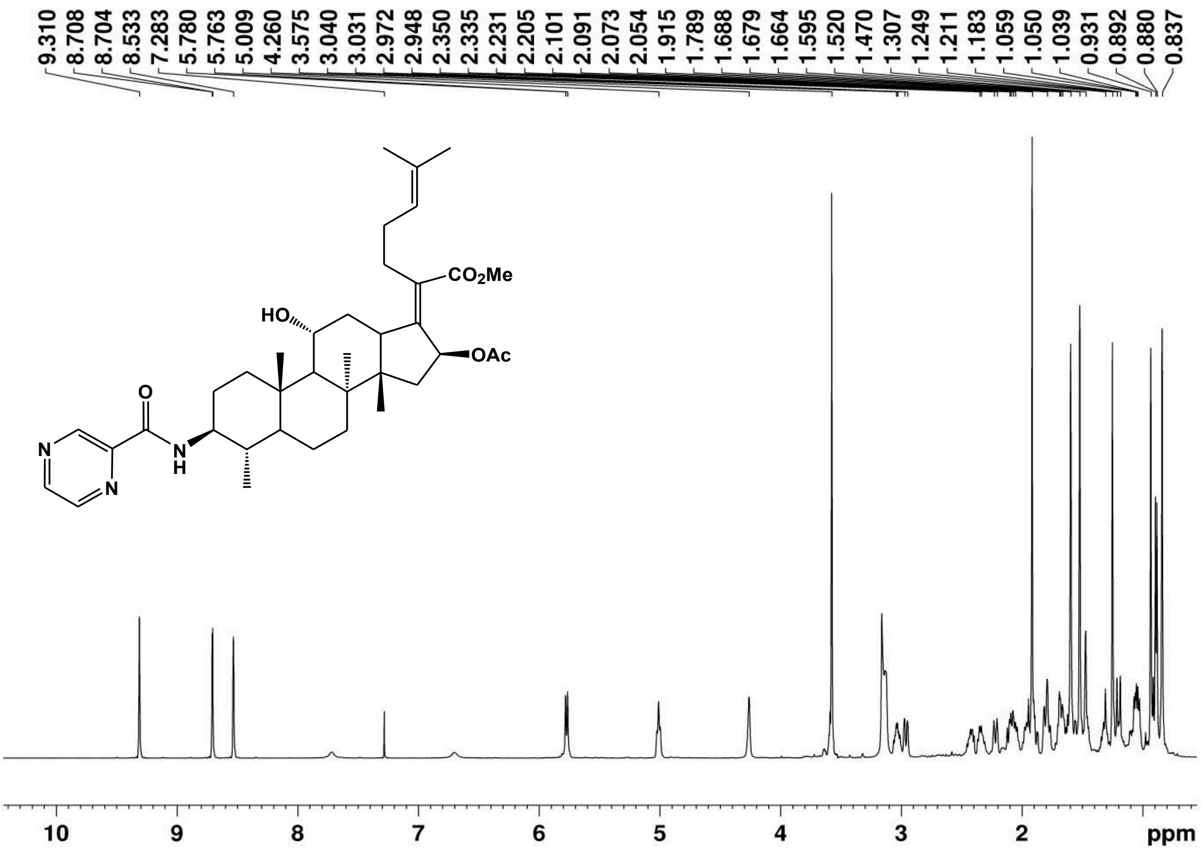

^{13}C NMR, 125.78 MHz, $CDCl_3$

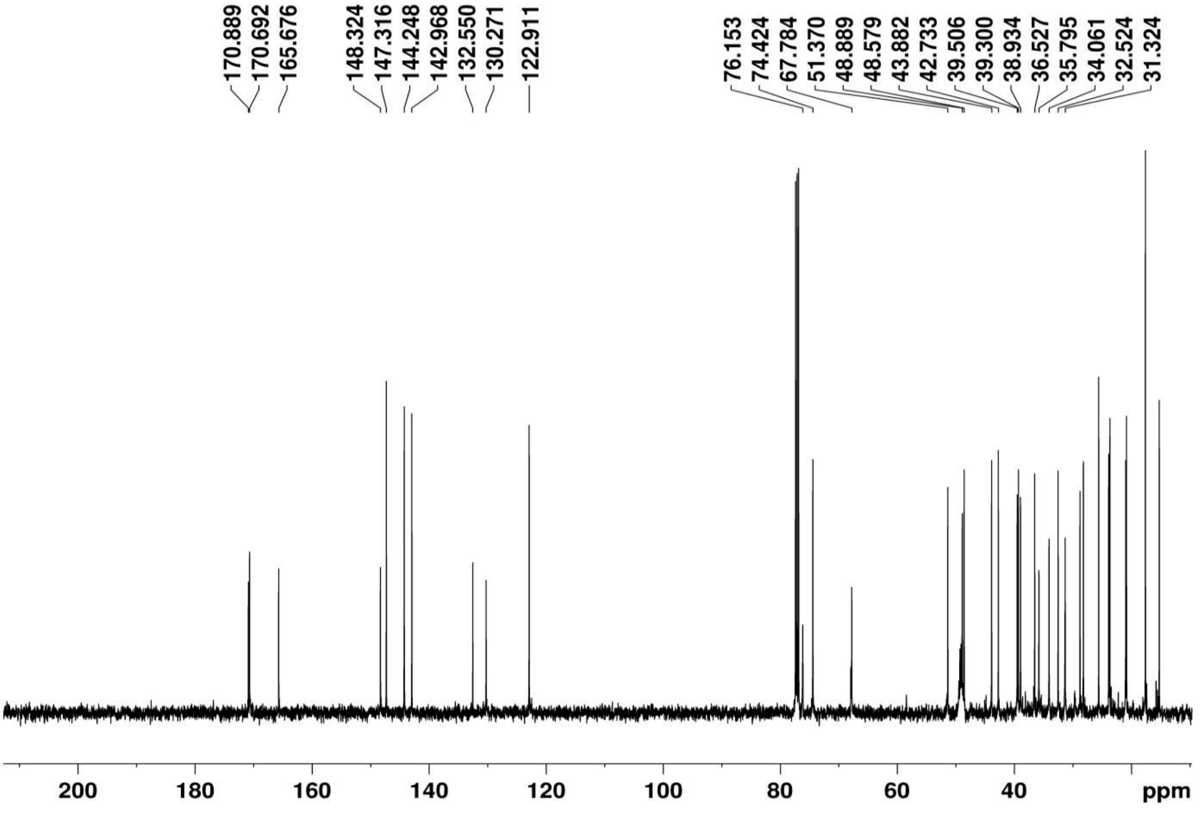

Compound **5:** ^{1}H NMR, 500.17 MHz, $CDCl_3$

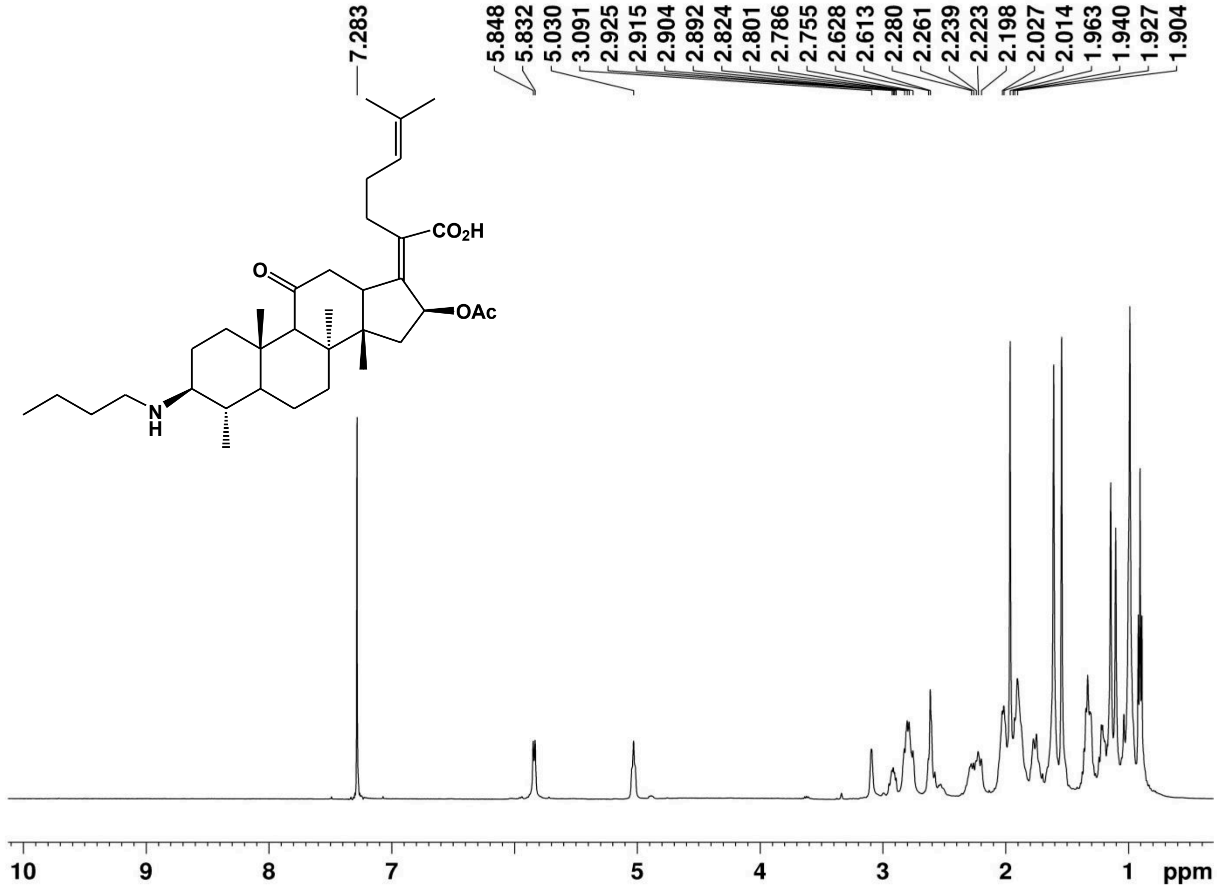

^{13}C NMR, 125.78 MHz, $CDCl_3$

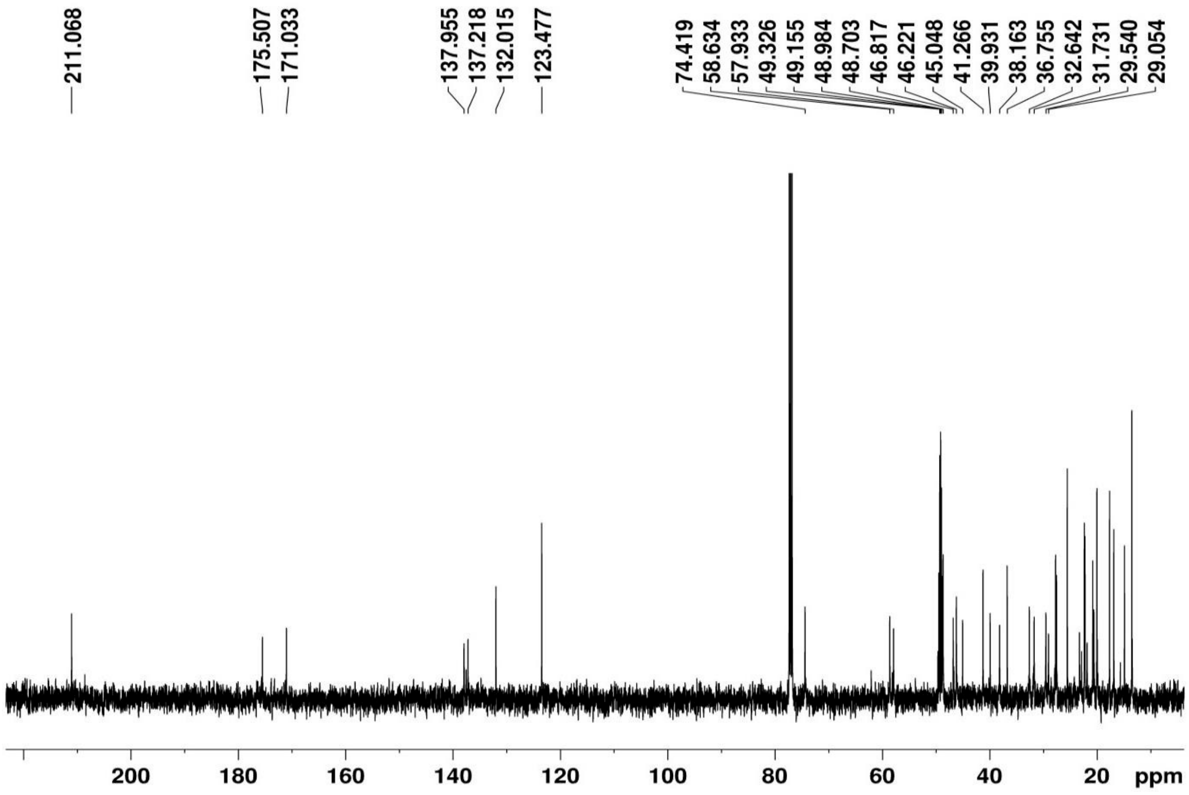

Compound **6:** ^{1}H NMR, 500.17 MHz, $CDCl_3$

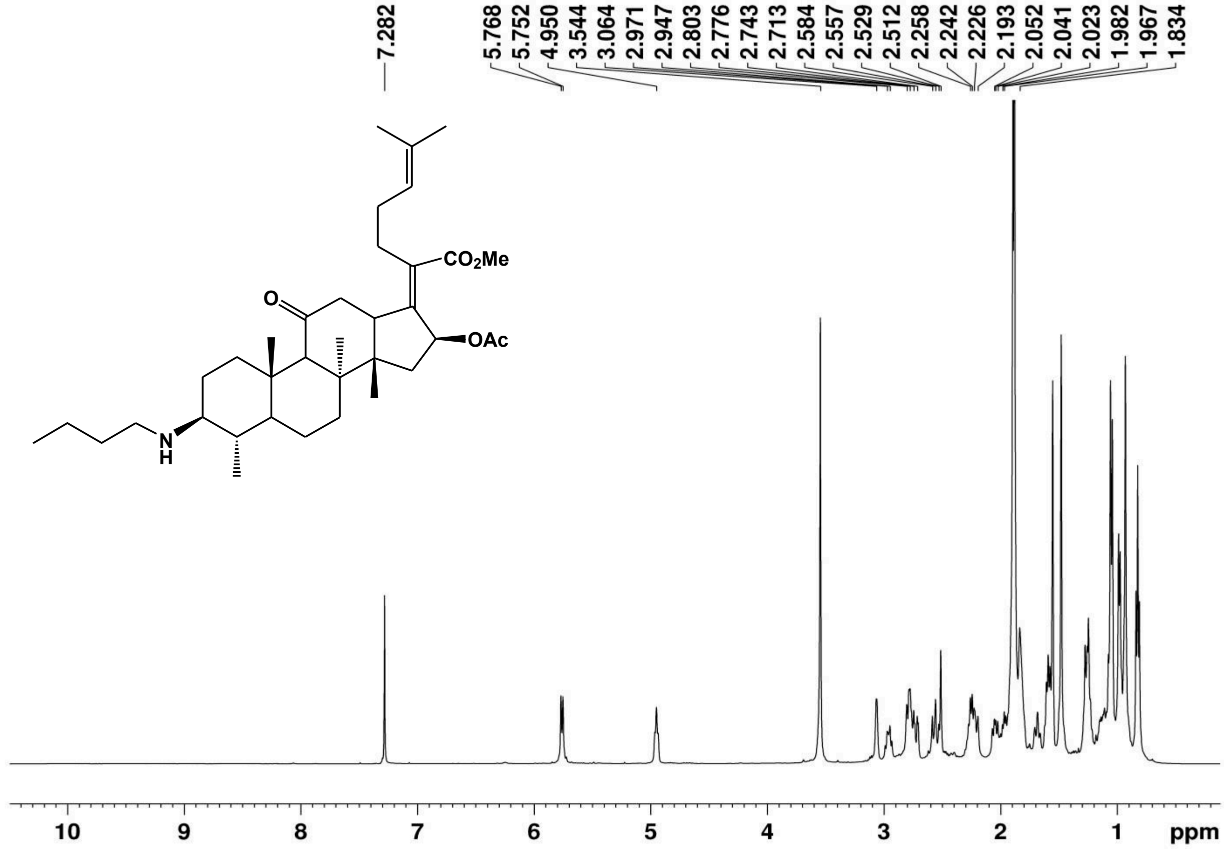

^{13}C NMR, 125.78 MHz, $CDCl_3$

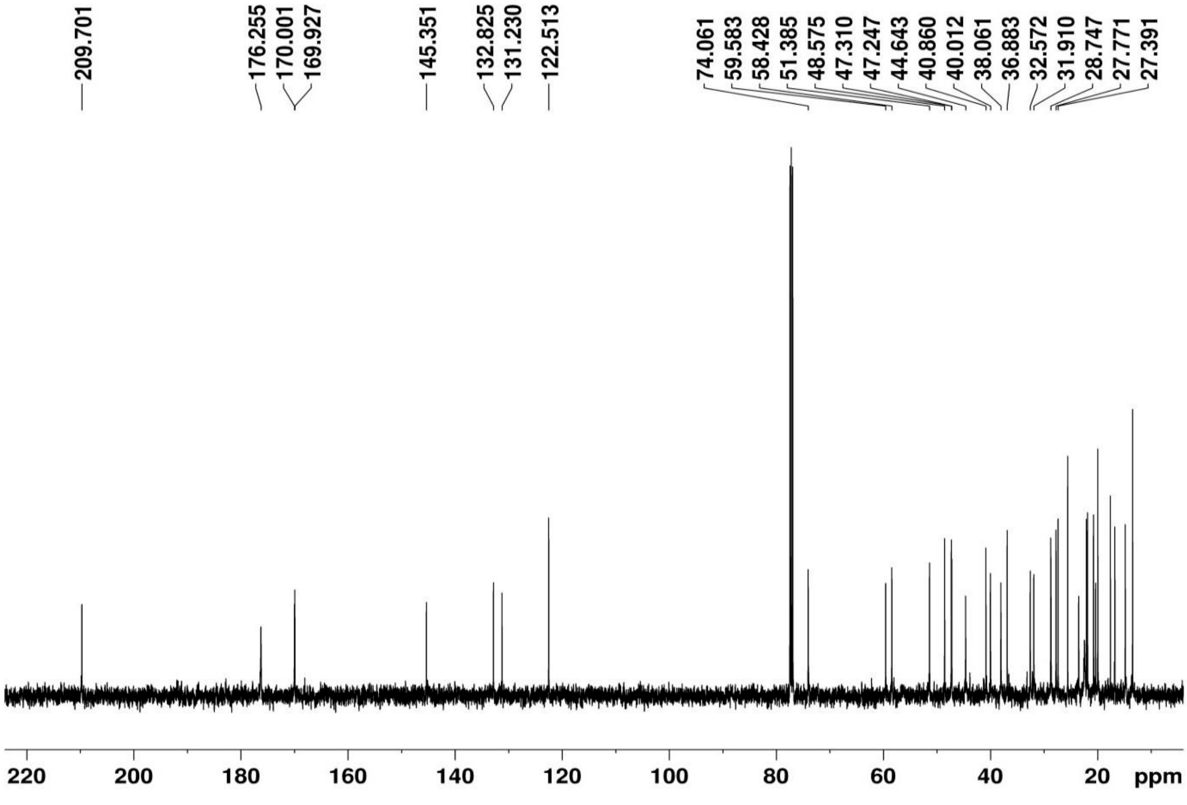

Compound **7:** ^{1}H NMR, 500.17 MHz, $CDCl_3$

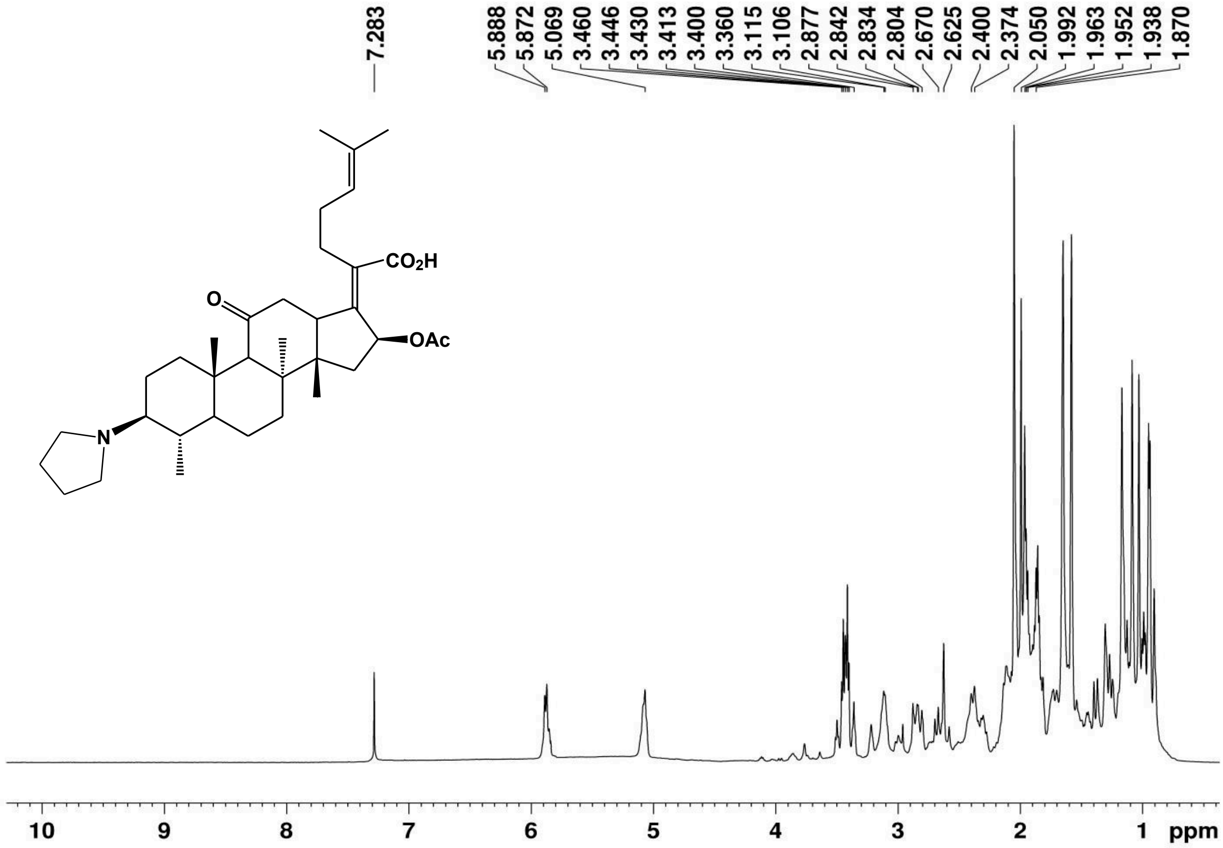

^{13}C NMR, 125.78 MHz, $CDCl_3$

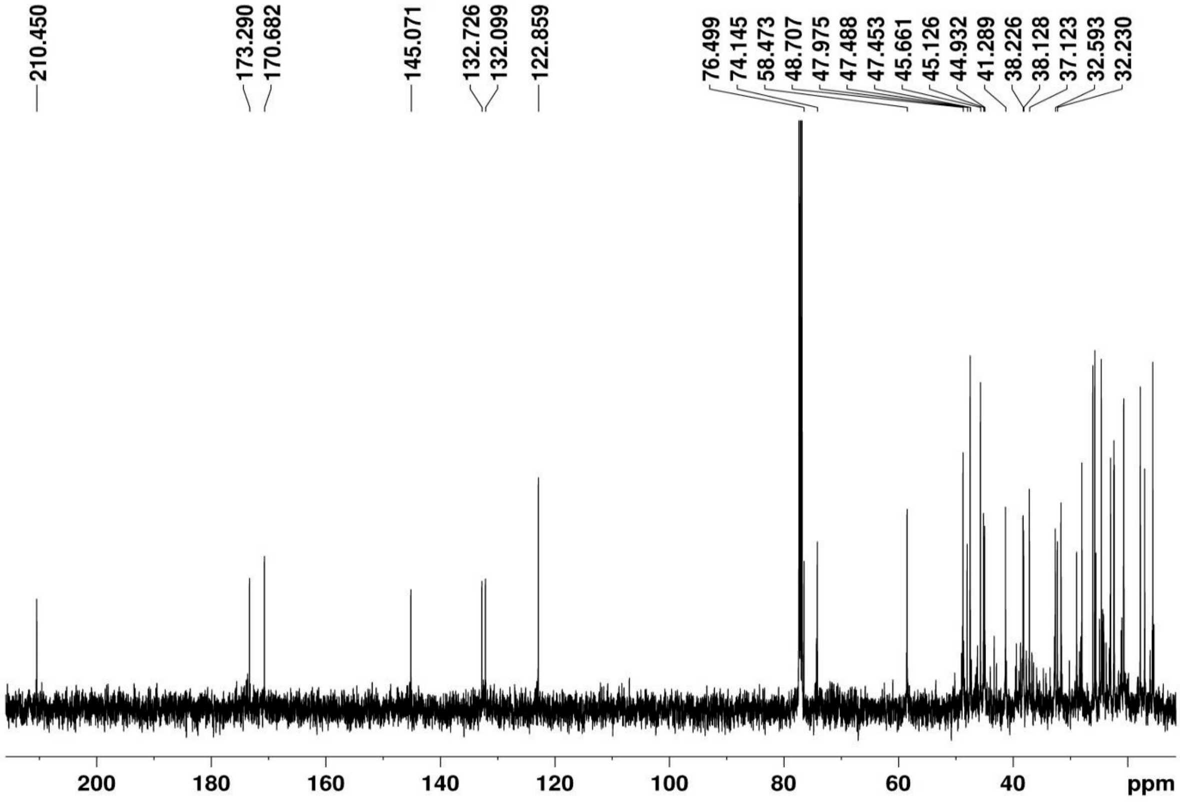

Compound **8:** ^{1}H NMR, 500.17 MHz, $CDCl_3$

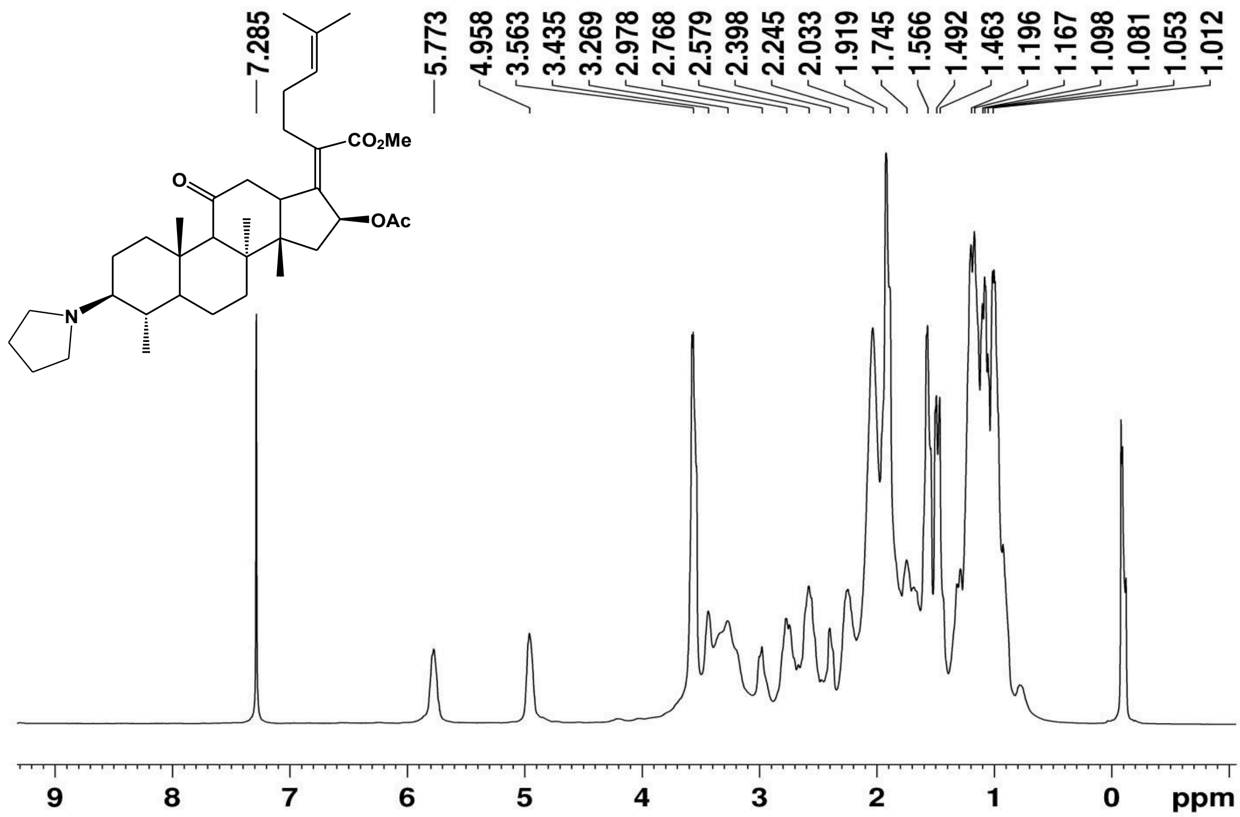

^{13}C NMR, 125.78 MHz, $CDCl_3$

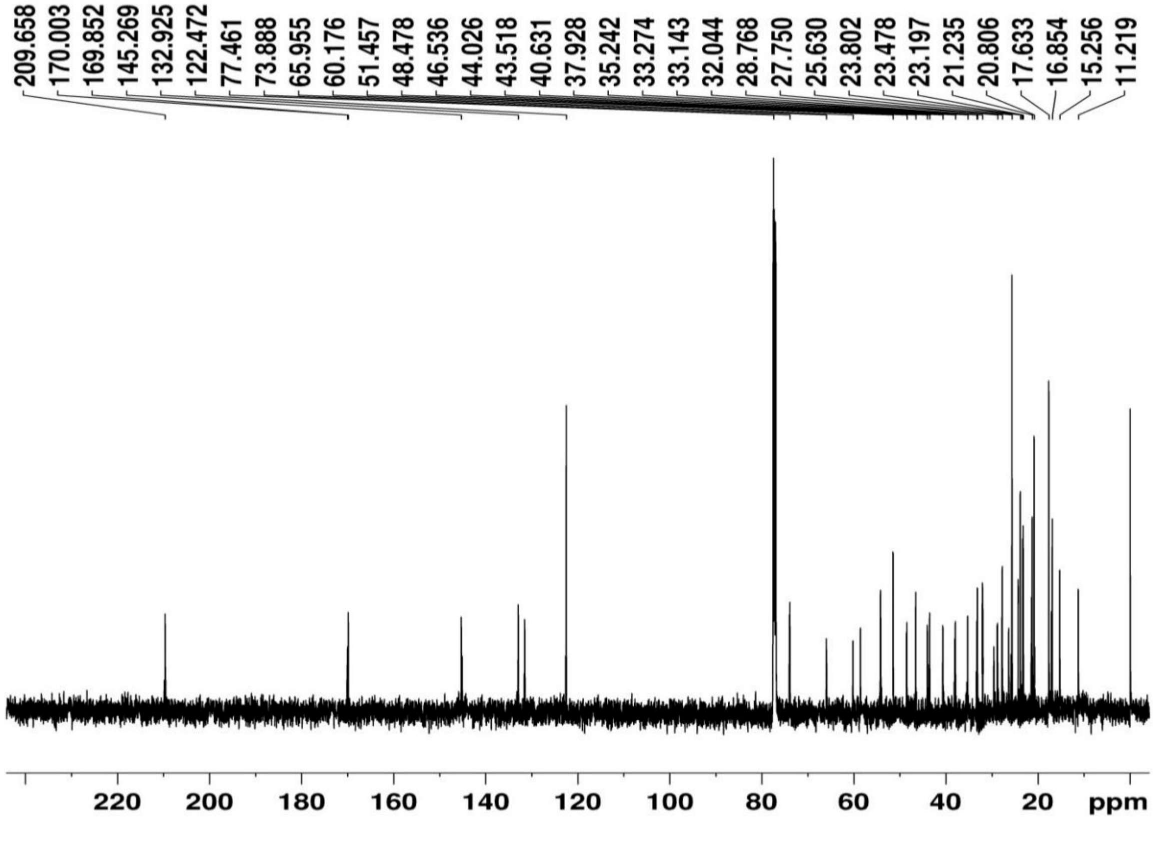

Compound **9:** ^{1}H NMR, 500.17 MHz, CD_3OD

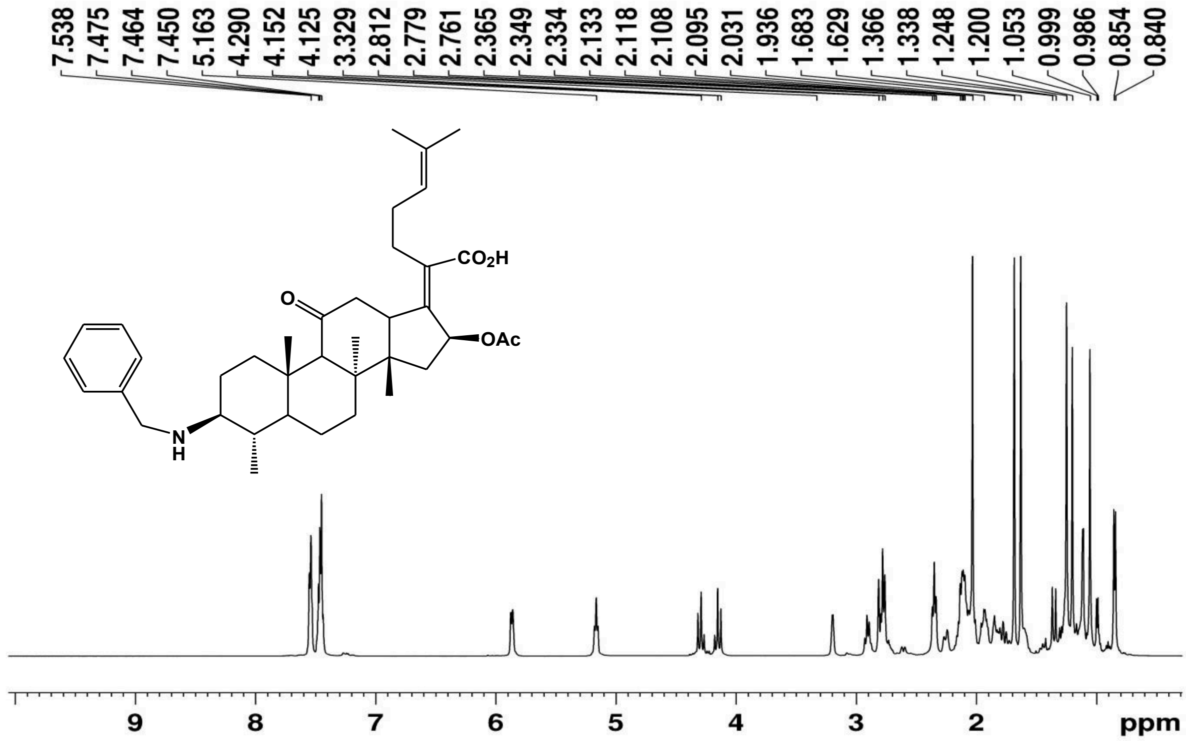

^{13}C NMR, 125.78 MHz, CD_3OD

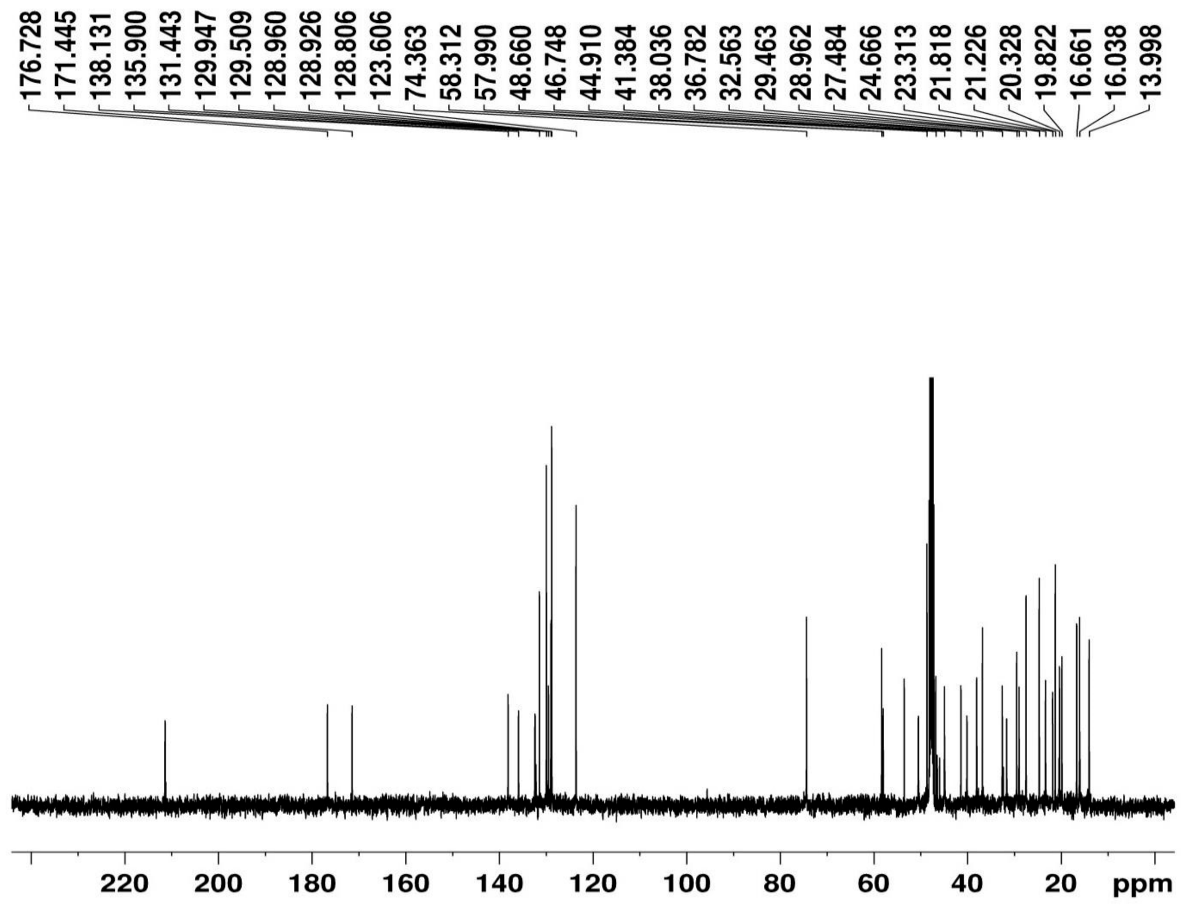

Compound **10:** ^{1}H NMR, 500.17 MHz, $CDCl_3$

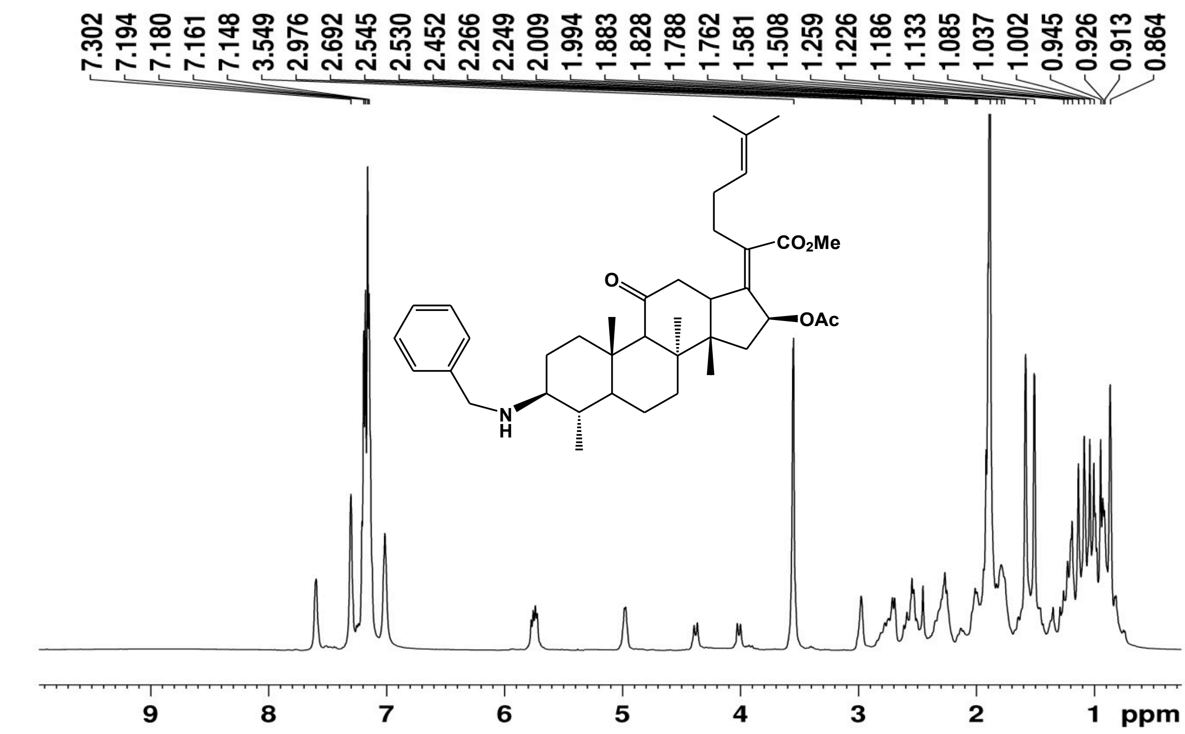

^{13}C NMR, 125.78 MHz, $CDCl_3$

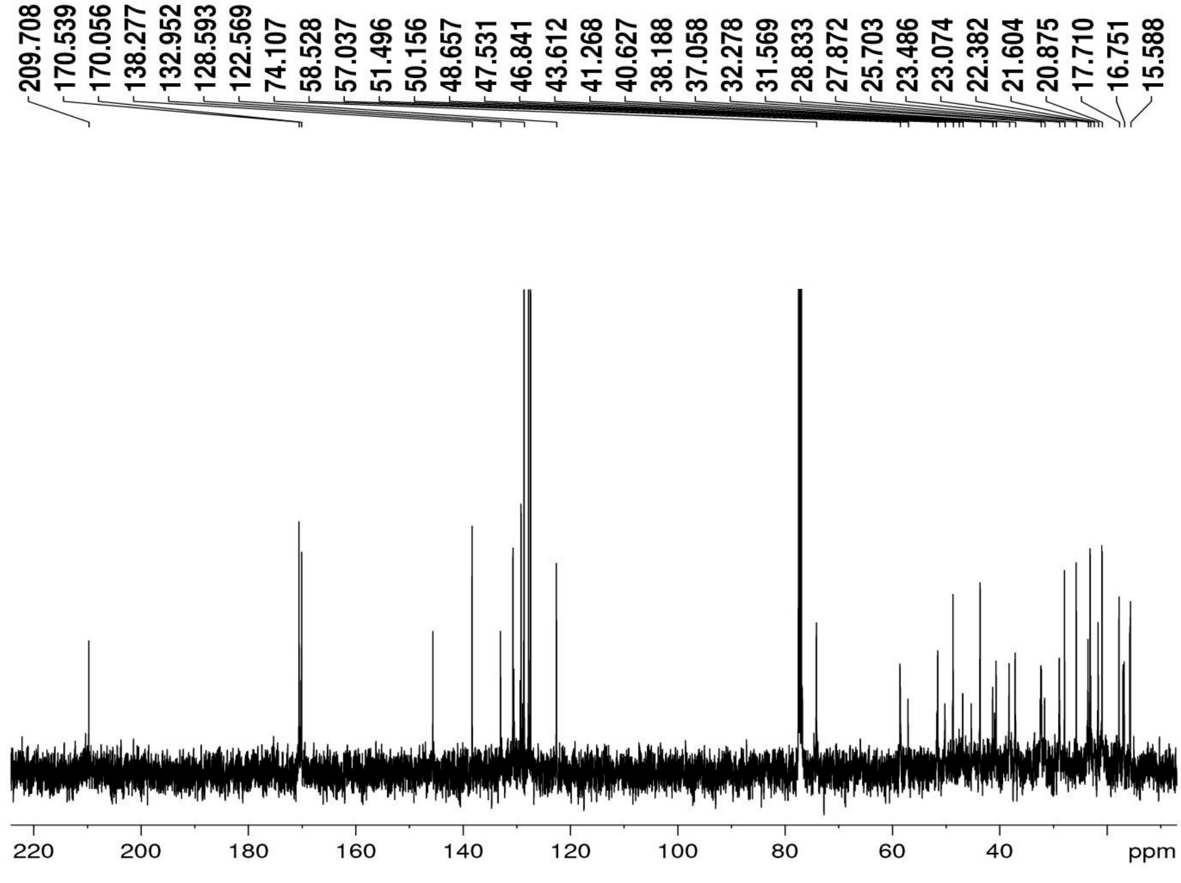

Compound **11:** ^{1}H NMR, 500.17 MHz, CD_3OD

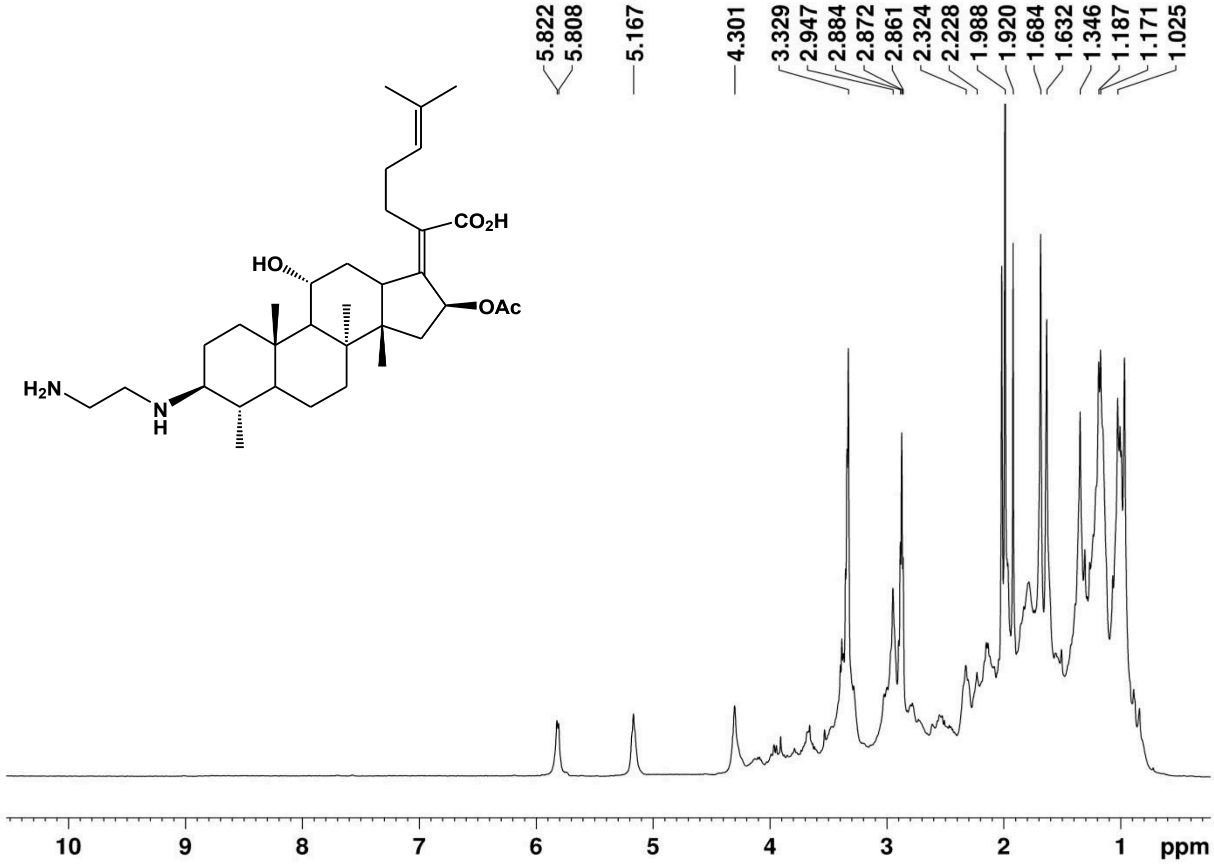

^{13}C NMR, 125.78 MHz, CD_3OD

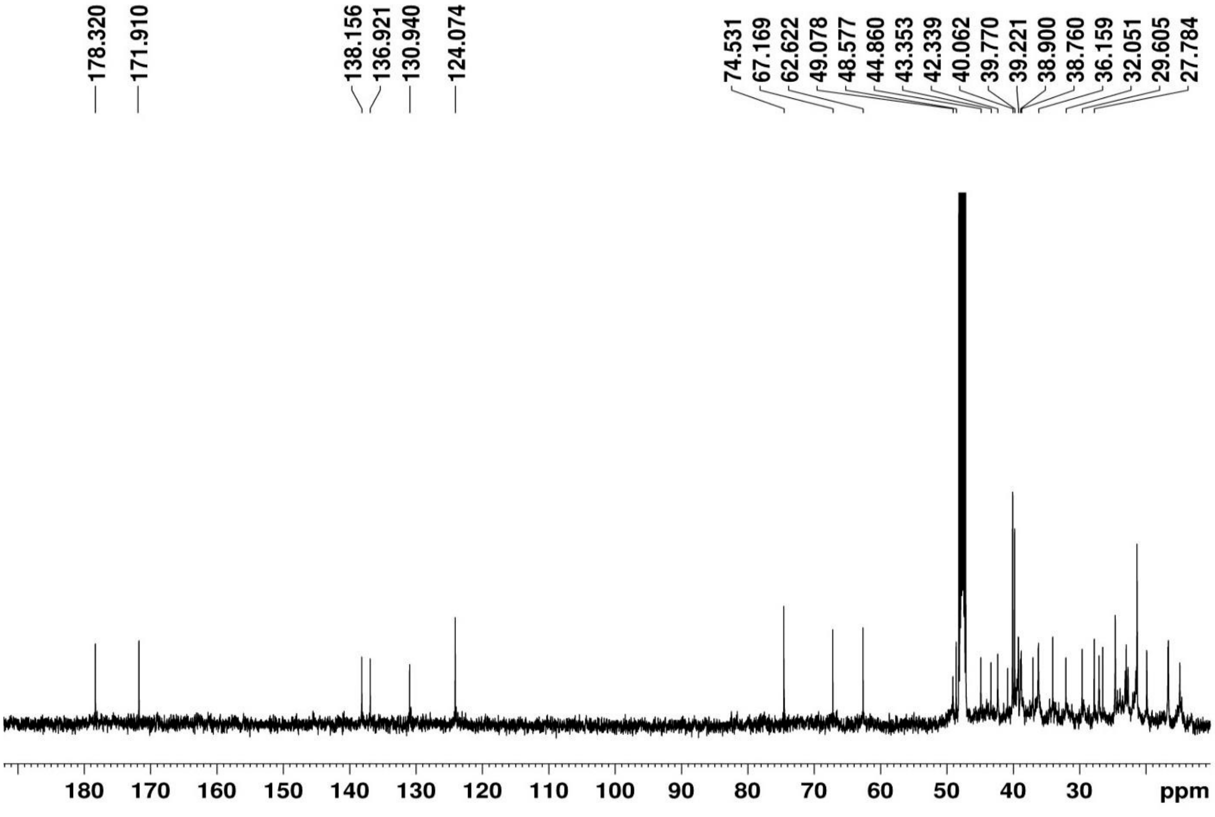

Compound **12:** ^{1}H NMR, 500.17 MHz, $CDCl_3$

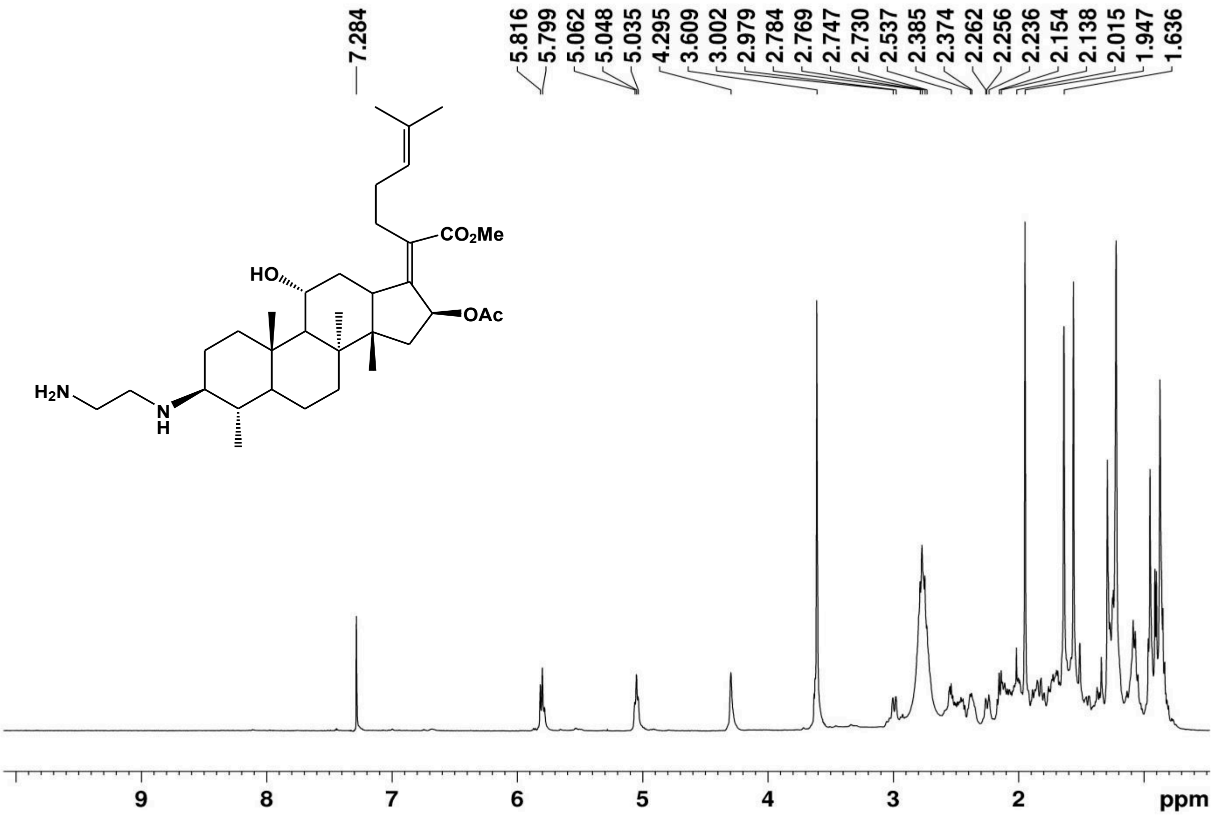

^{13}C NMR, 125.78 MHz, $CDCl_3$

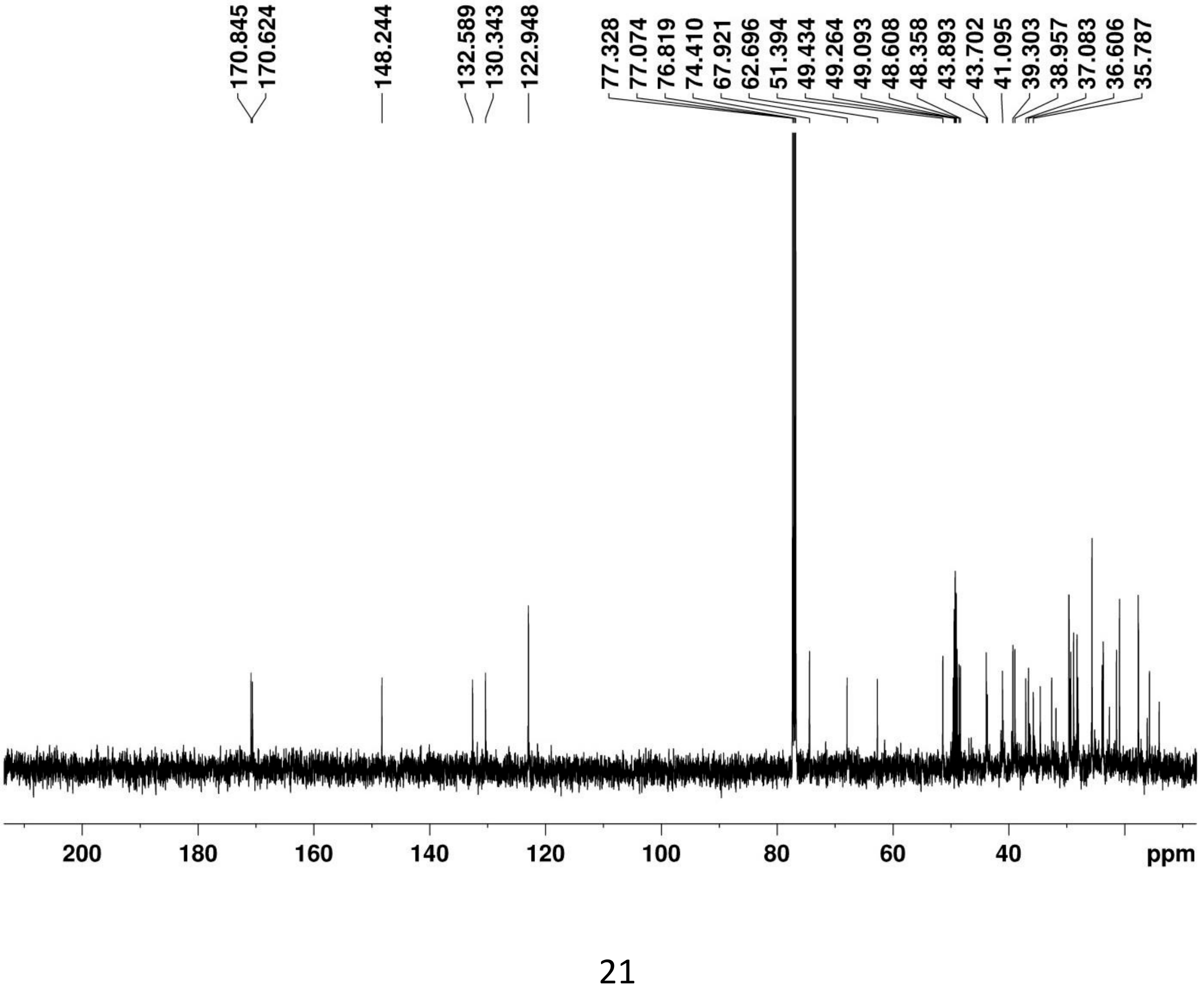

Compound **13:** ^{1}H NMR, 500.17 MHz, $CDCl_3$

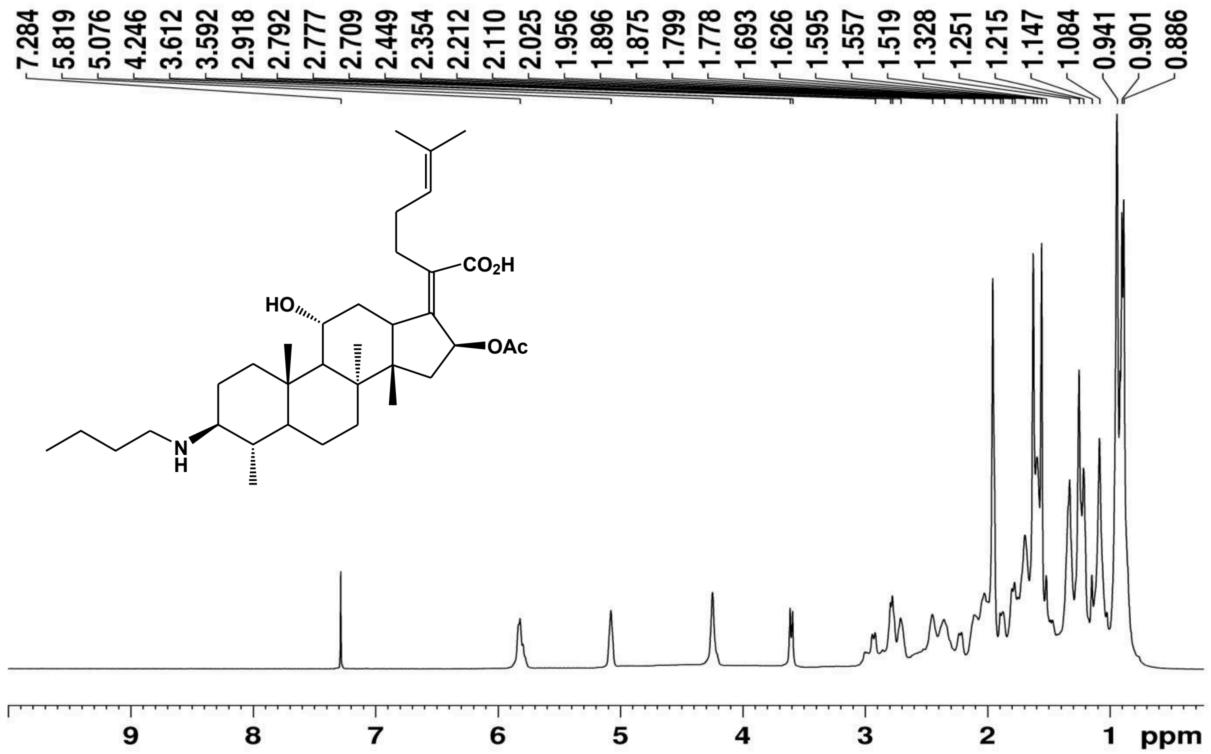

^{13}C NMR, 125.78 MHz, $CDCl_3$

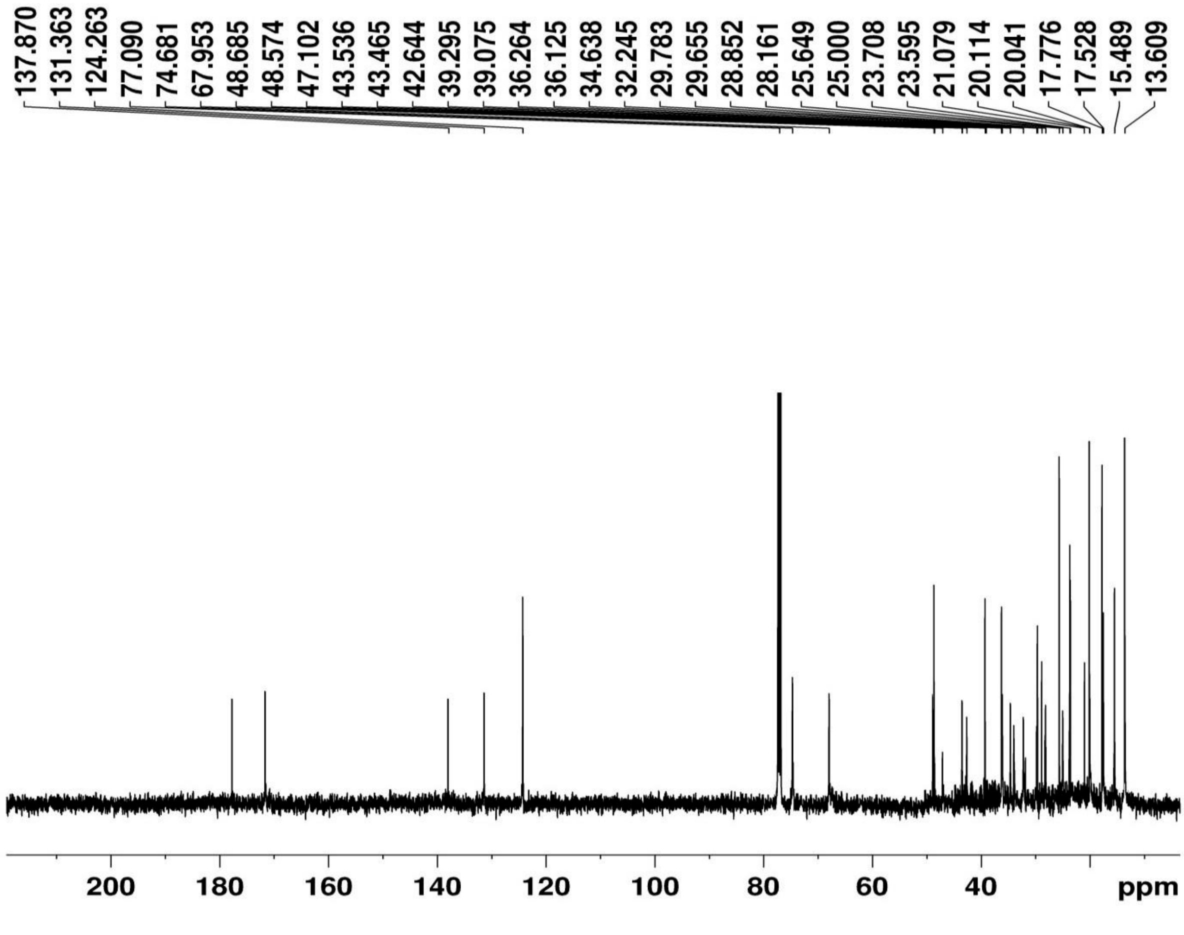

Compound **14:** ^{1}H NMR, 500.17 MHz, $CDCl_3$

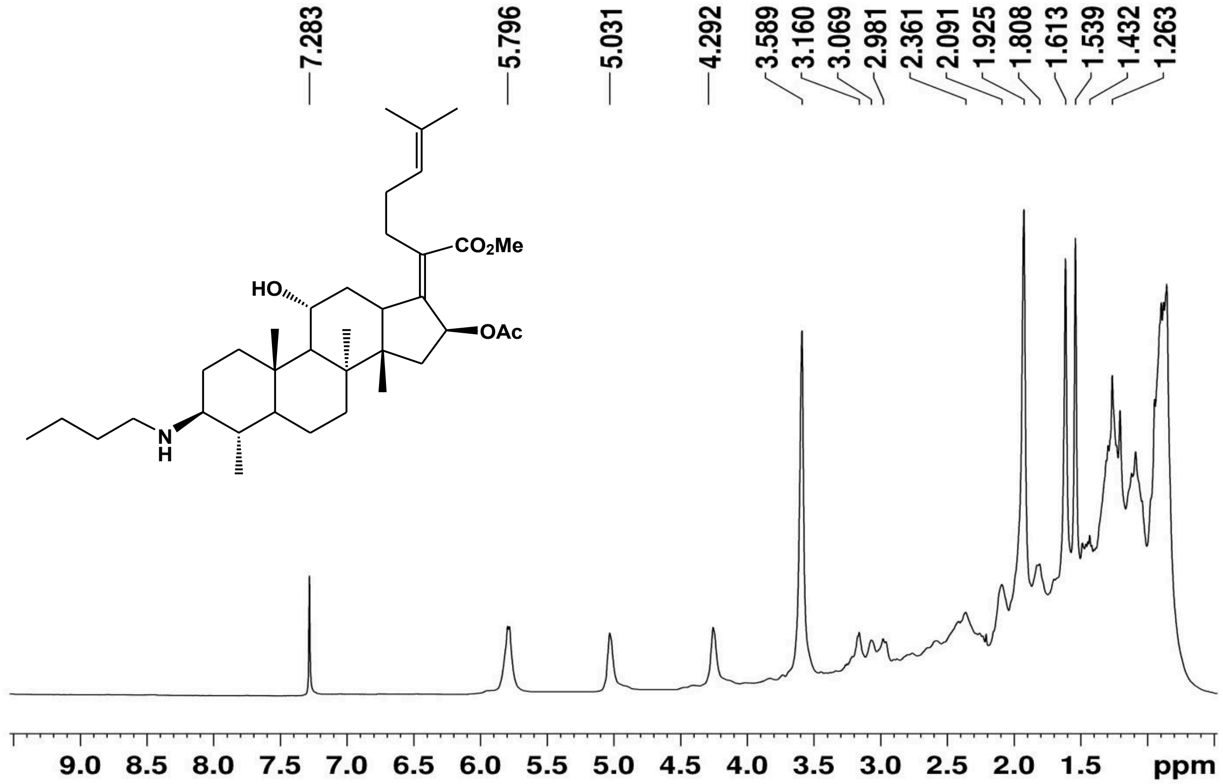

^{13}C NMR, 125.78 MHz, $CDCl_3$

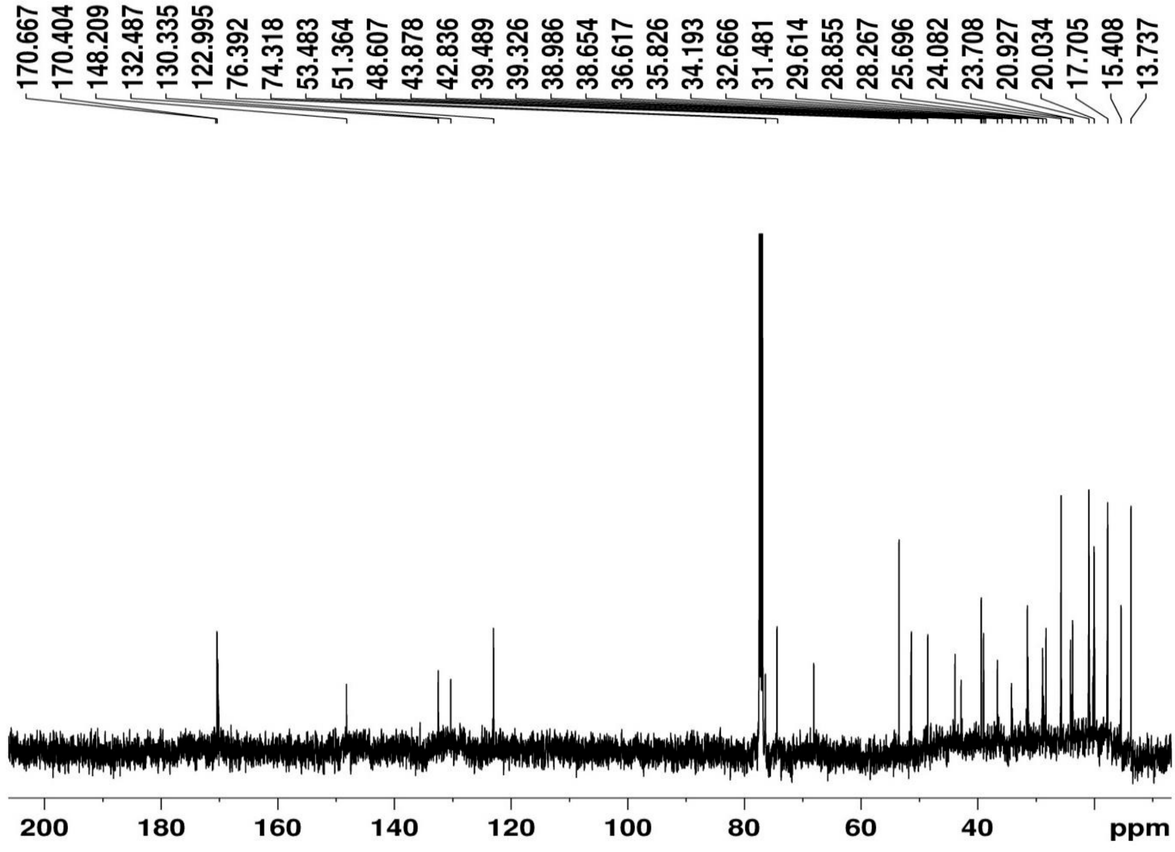

Compound **15:** ^{1}H NMR, 500.17 MHz, $CDCl_3$

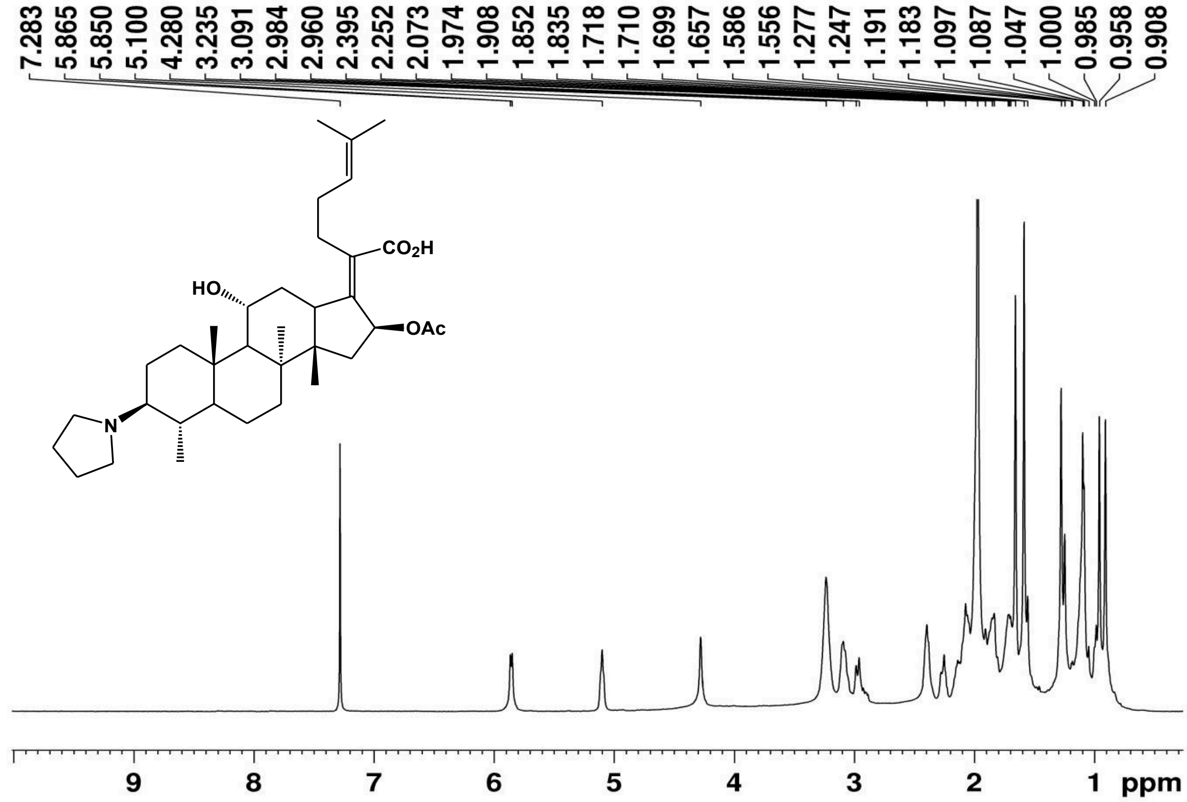

^{13}C NMR, 125.78 MHz, $CDCl_3$

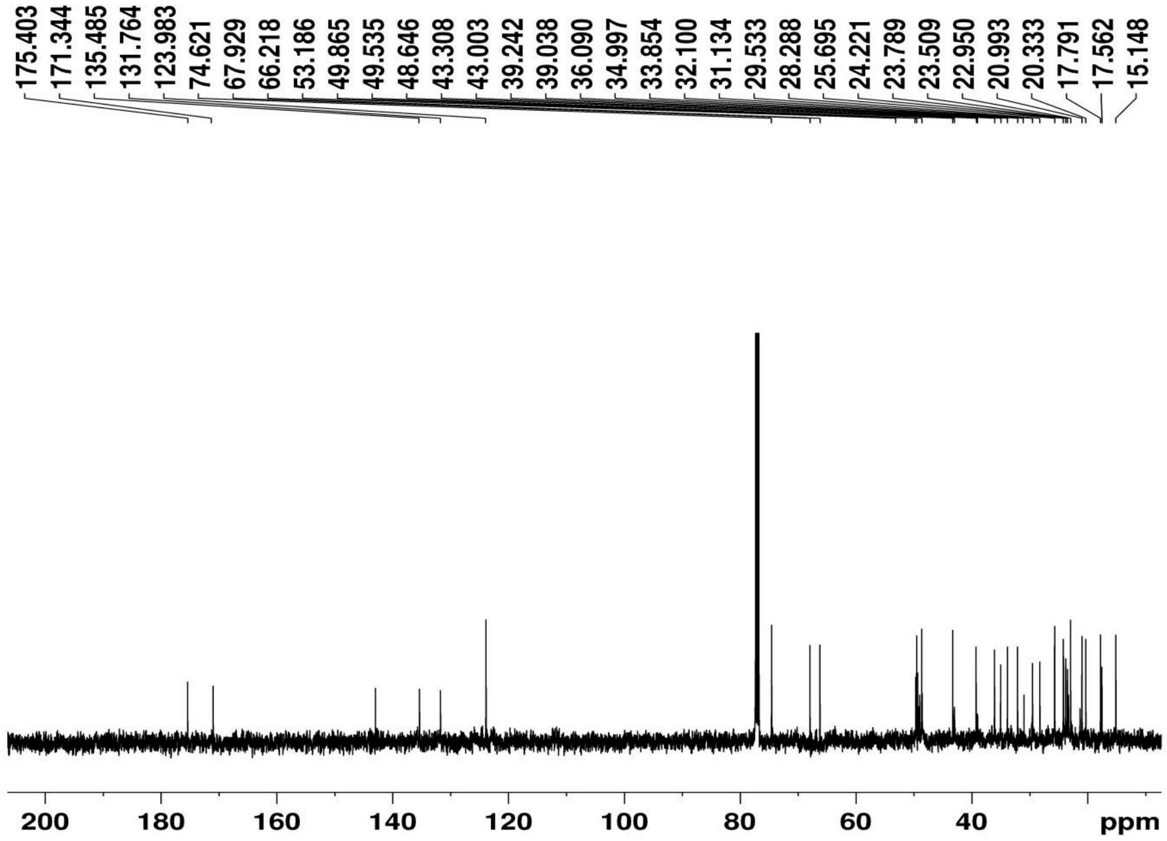

Compound **16:** ^{1}H NMR, 500.17 MHz, $CDCl_3$

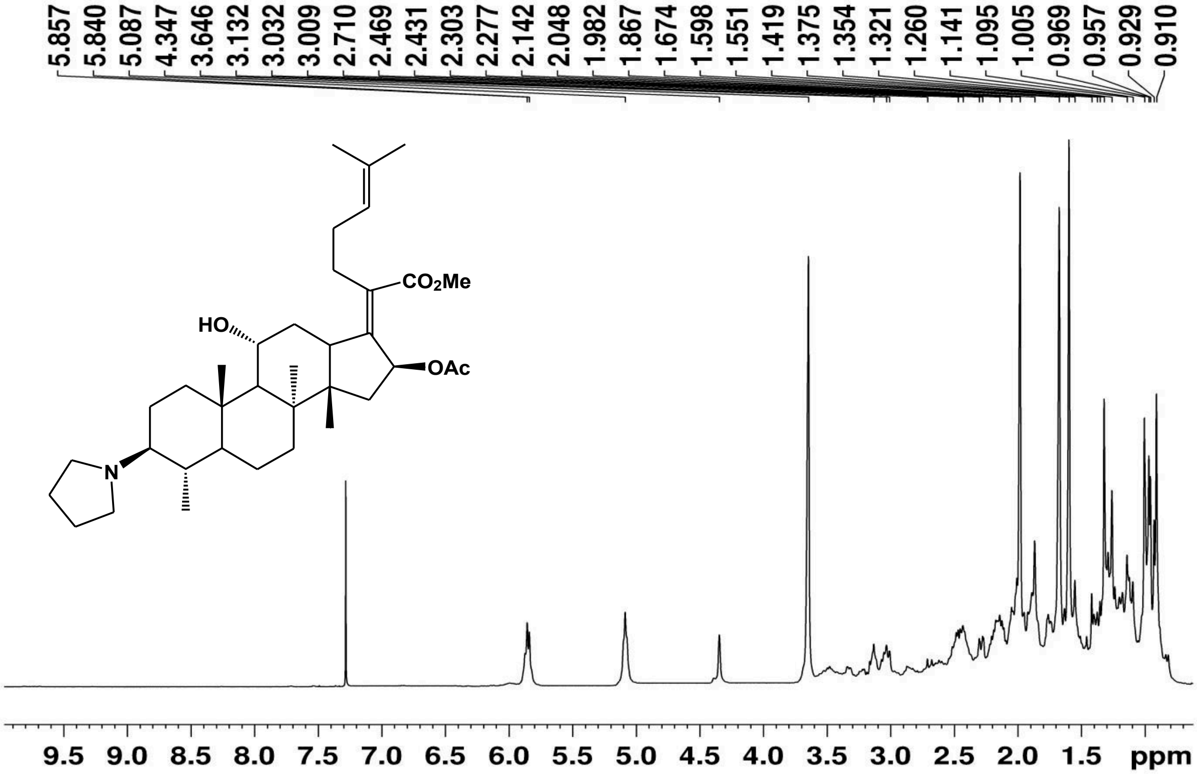

^{13}C NMR, 125.78 MHz, $CDCl_3$

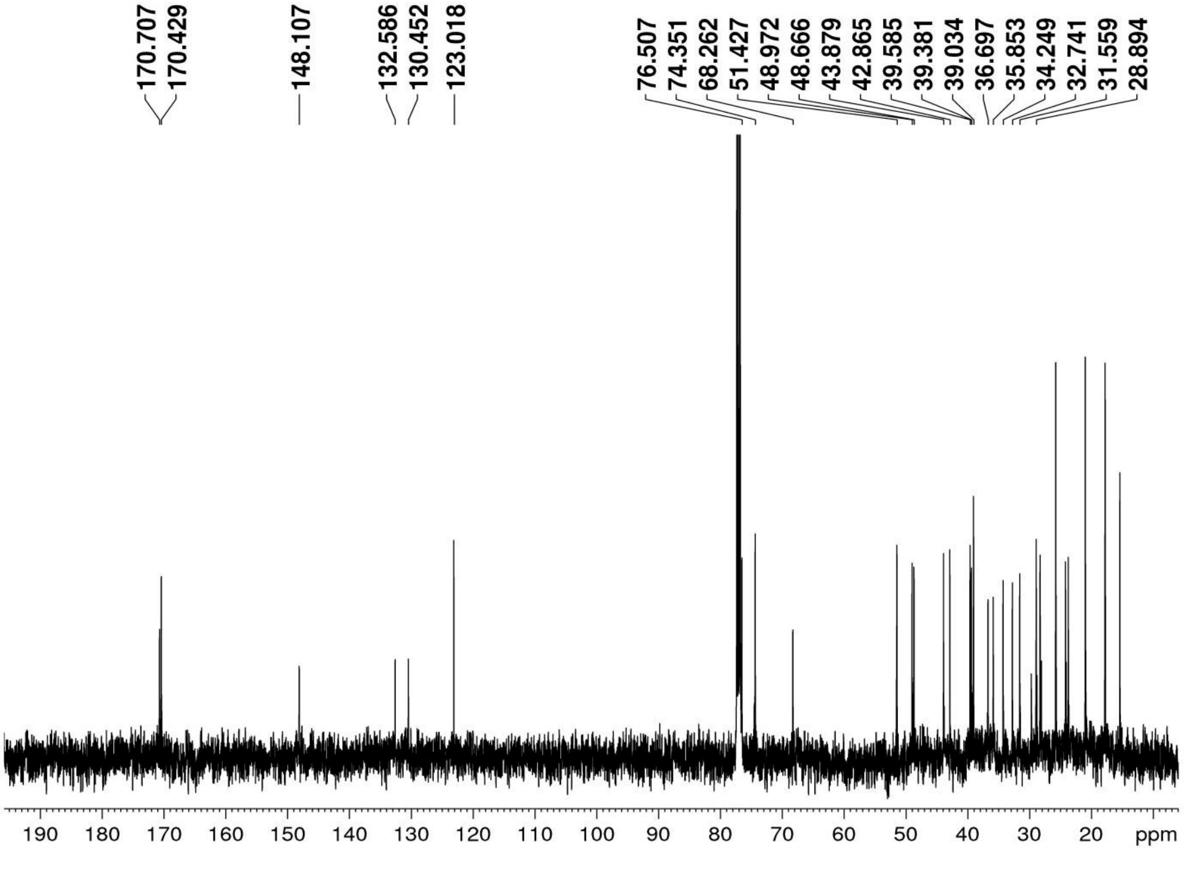

Compound **17:** ^{1}H NMR, 500.17 MHz, $CDCl_3$

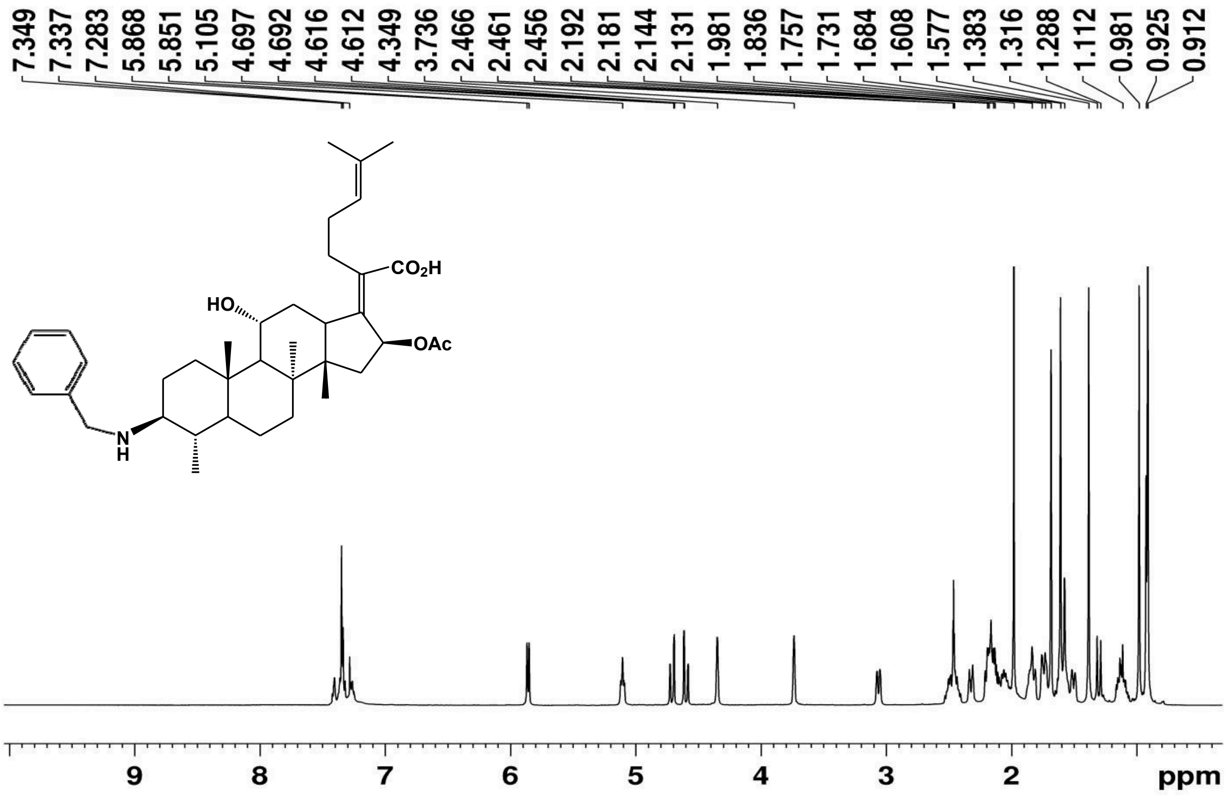

^{13}C NMR, 125.78 MHz, $CDCl_3$

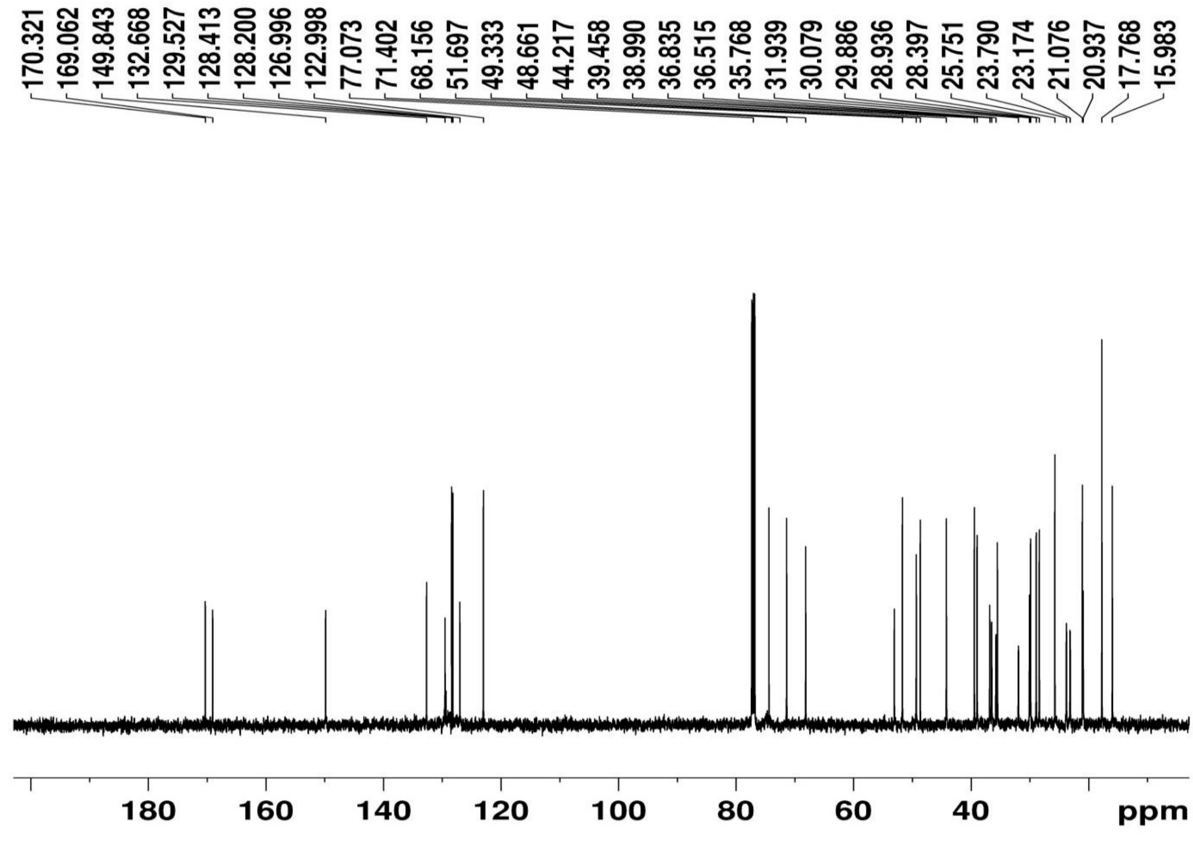

Compound **18:** ^{1}H NMR, 500.17 MHz, $CDCl_3$

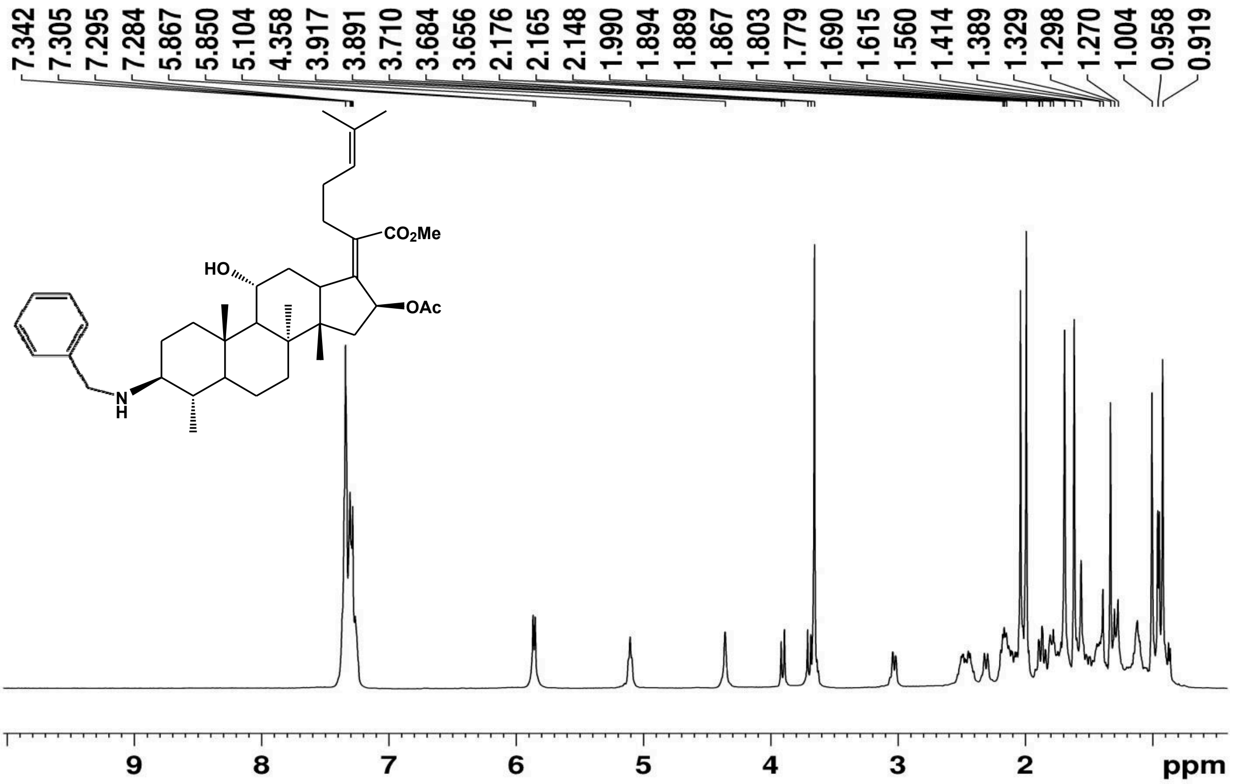

^{13}C NMR, 125.78 MHz, $CDCl_3$

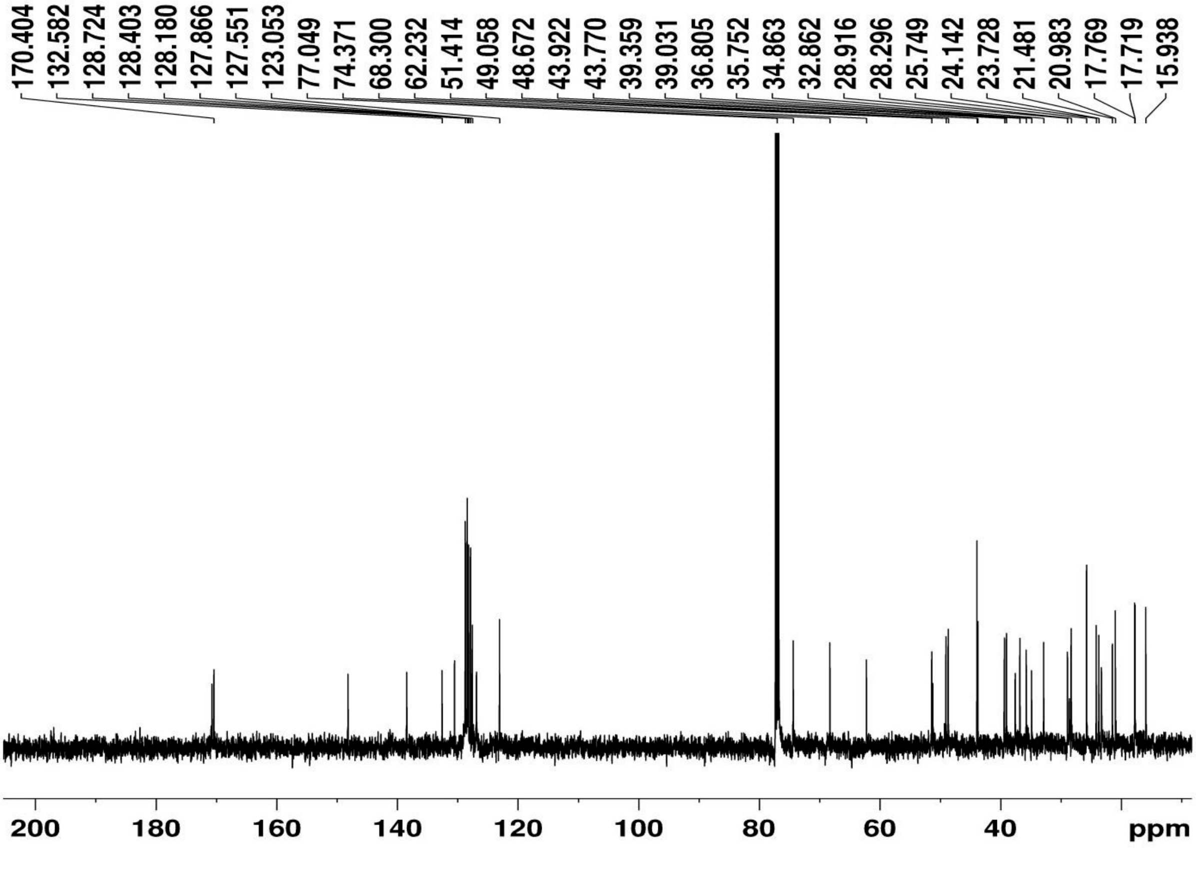

Compound **19:** ^{1}H NMR, 500.17 MHz, CD_3OD

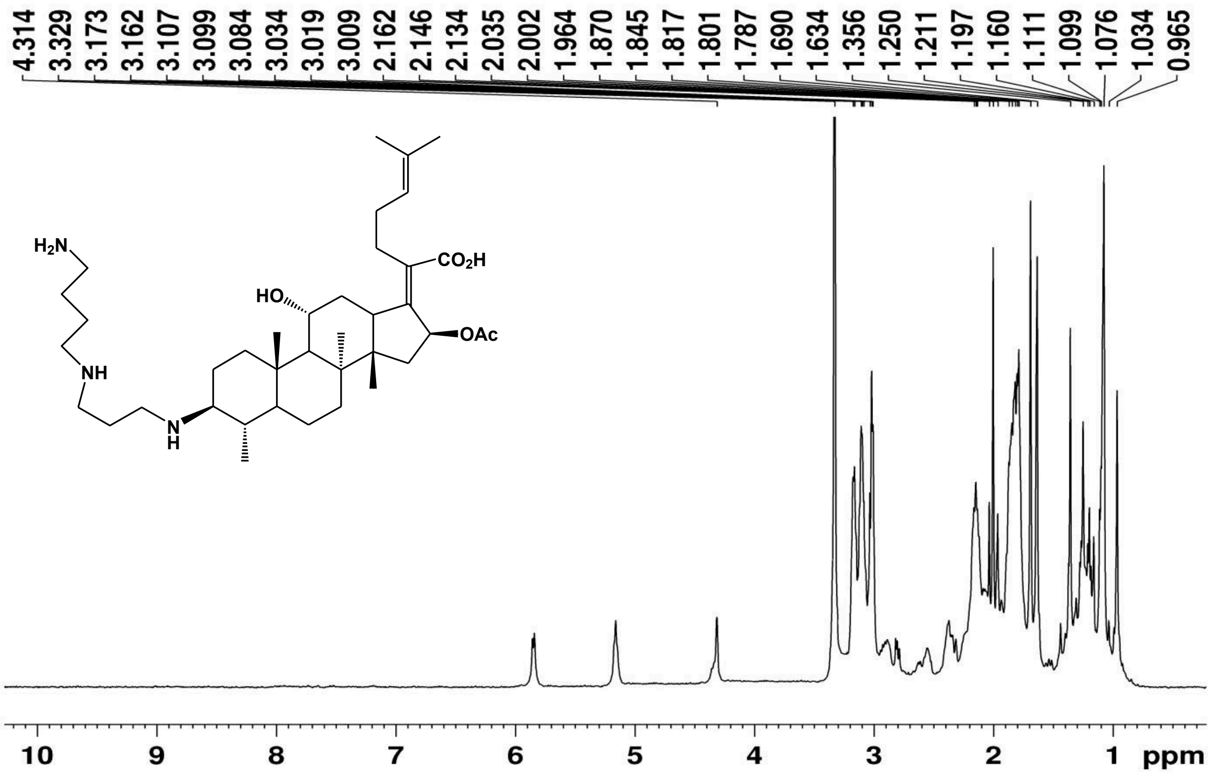

^{13}C NMR, 125.78 MHz, CD_3OD

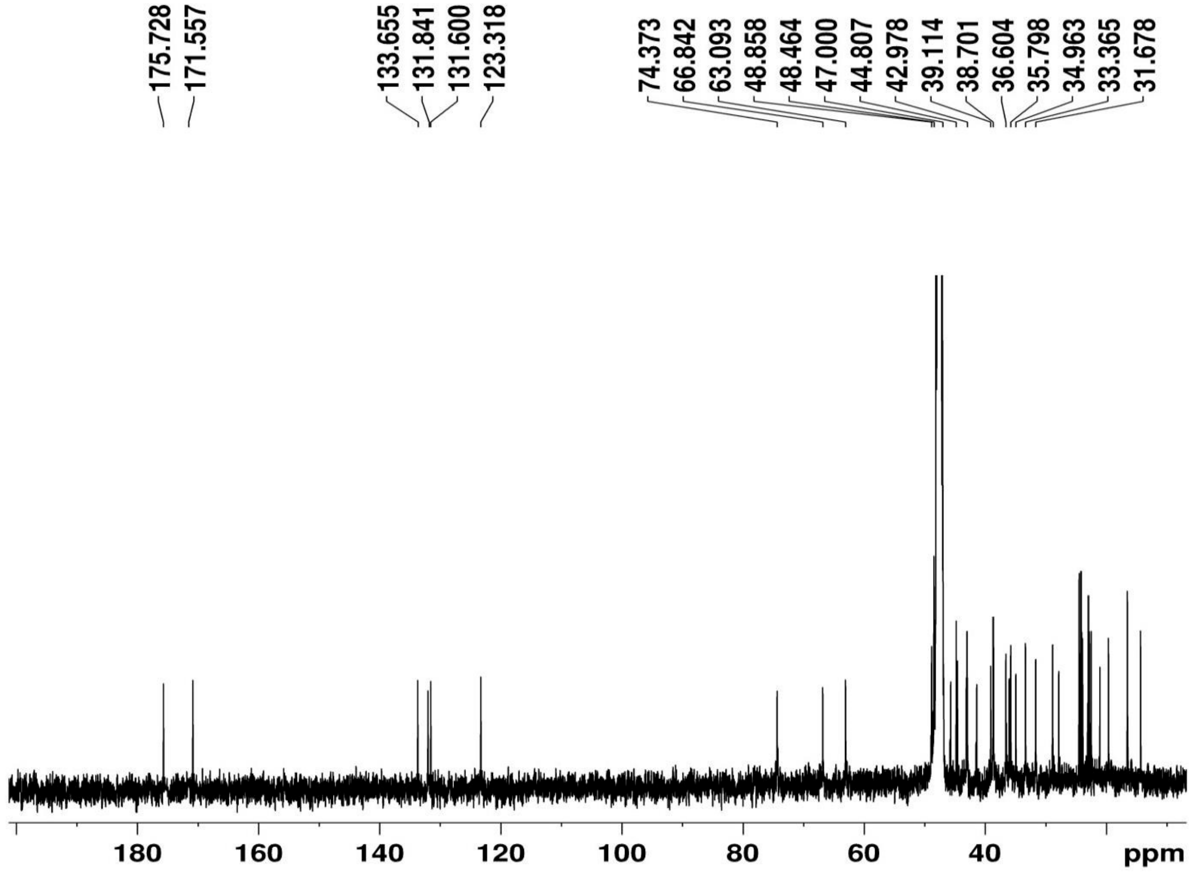

Compound **20:** ^{1}H NMR, 500.17 MHz, CD_3OD

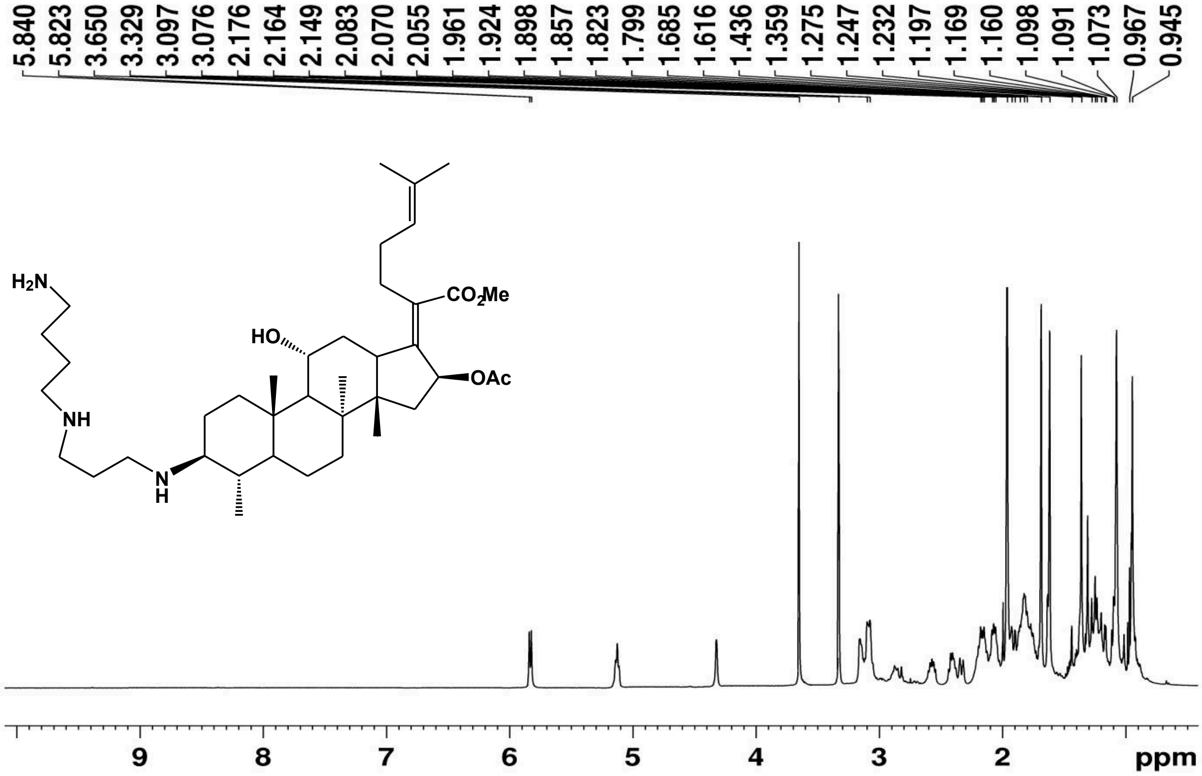

^{13}C NMR, 125.78 MHz, CD_3OD

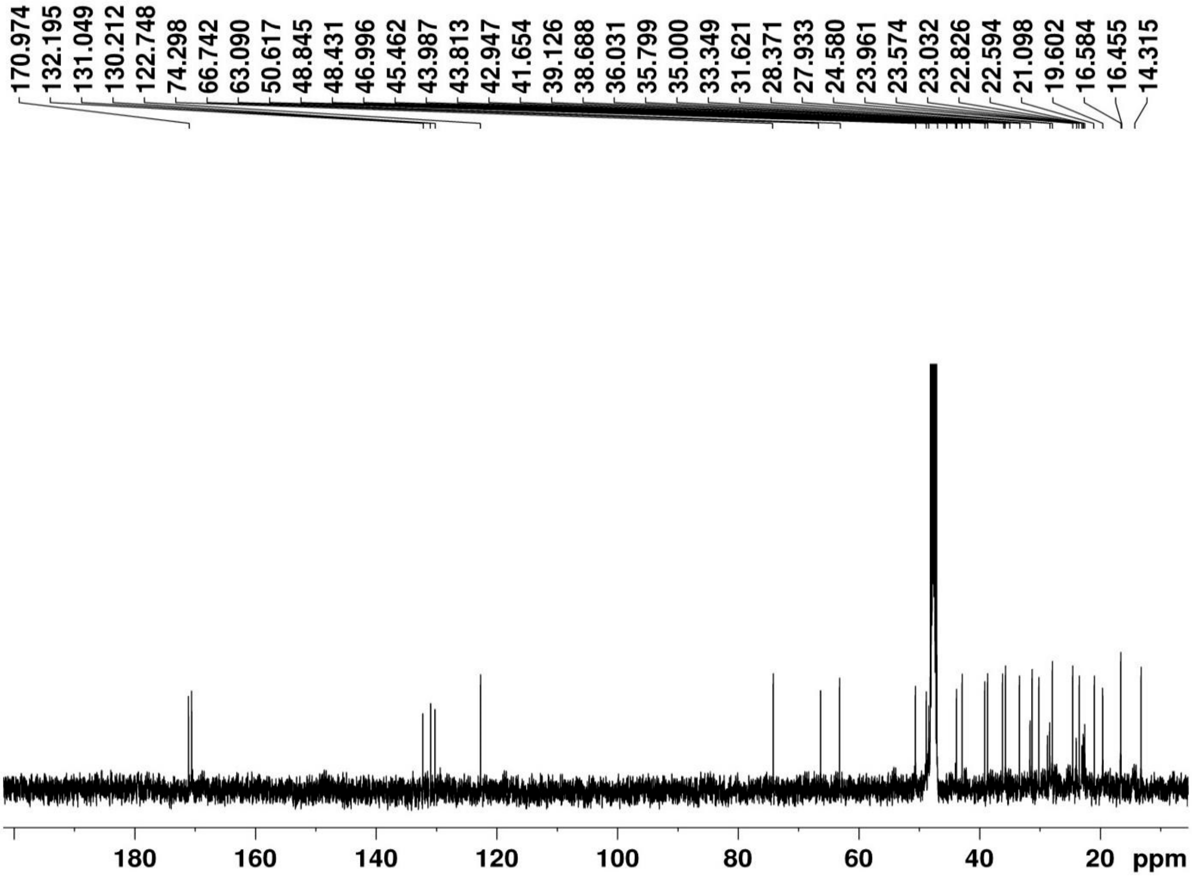

Compound **21:** ^{1}H NMR, 500.17 MHz, CD_3OD

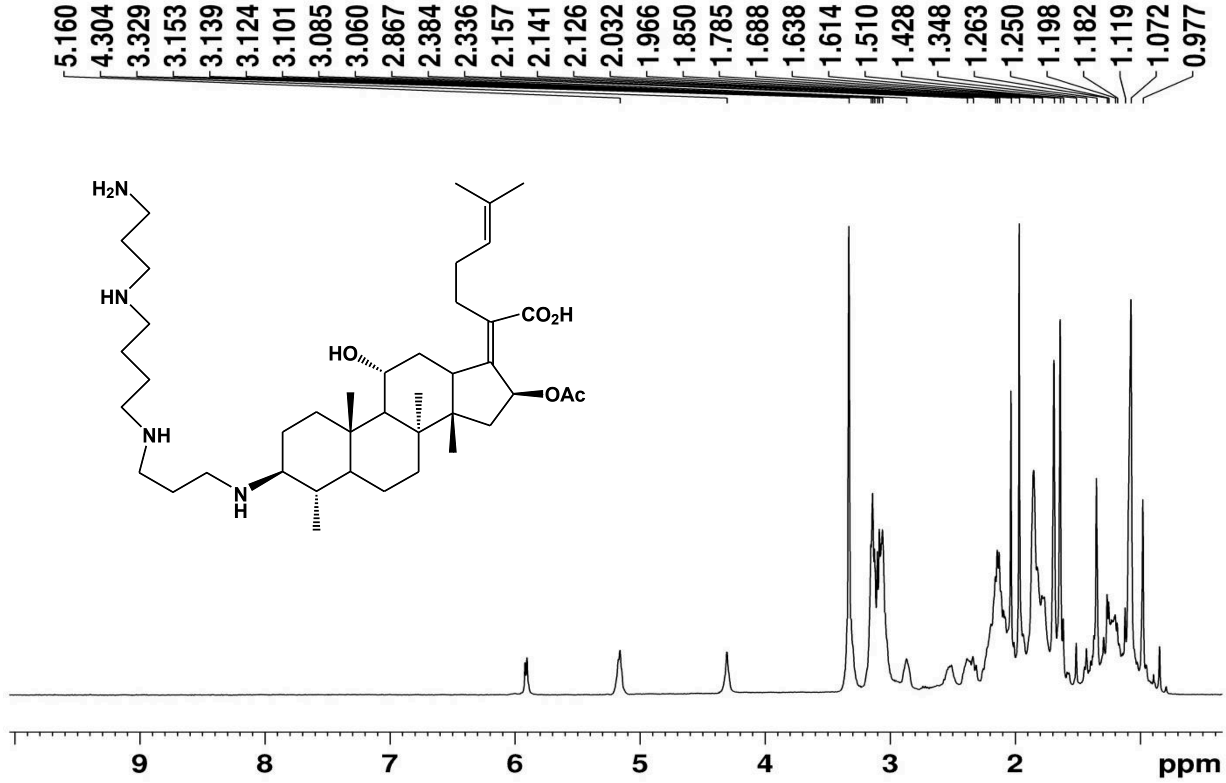

^{13}C NMR, 125.78 MHz, CD_3OD

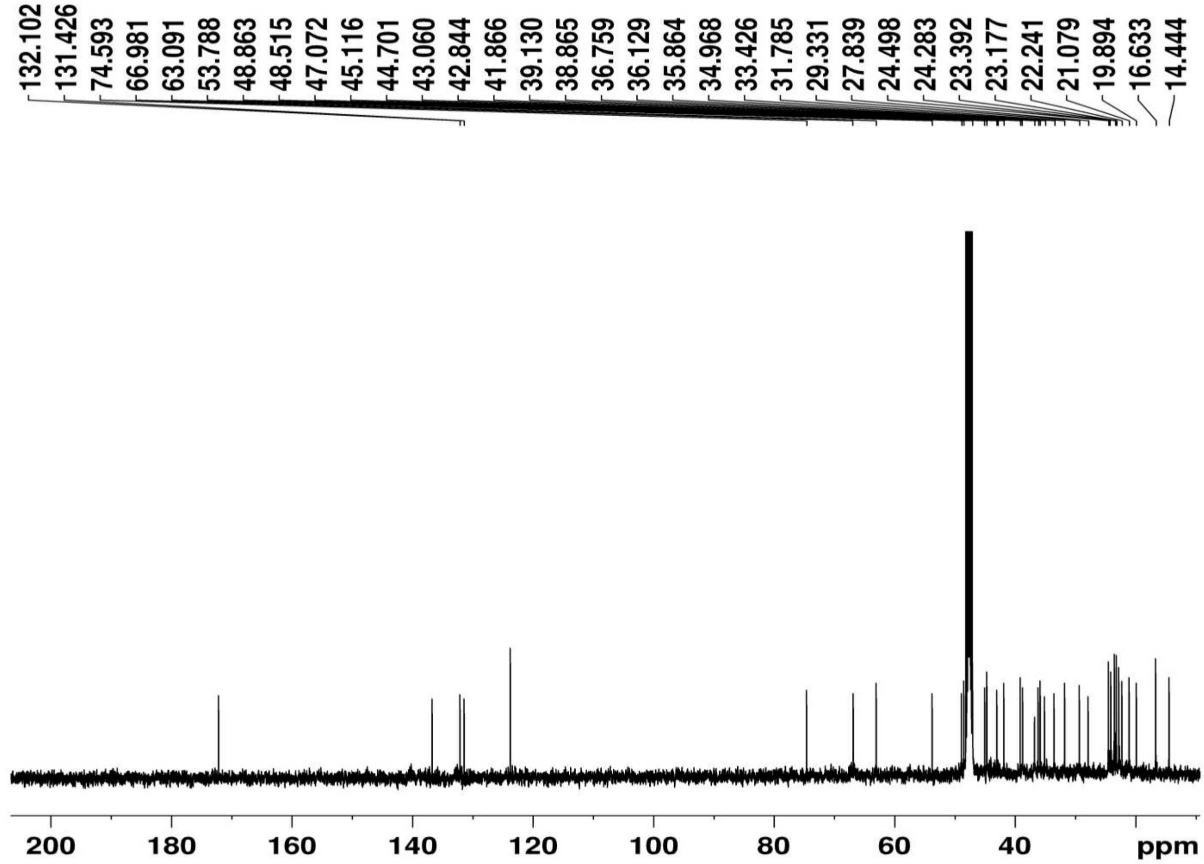

Compound **22:** ^{1}H NMR, 500.17 MHz, CD_3OD

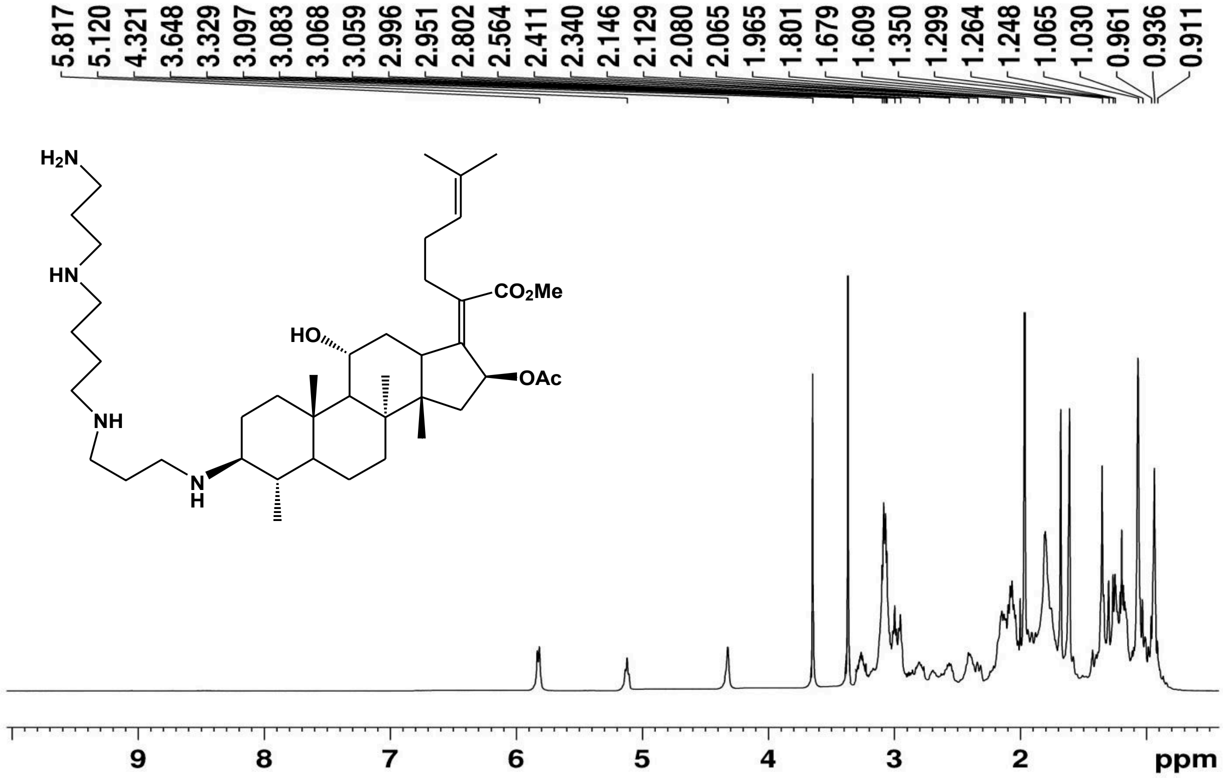

^{13}C NMR, 125.78 MHz, CD_3OD

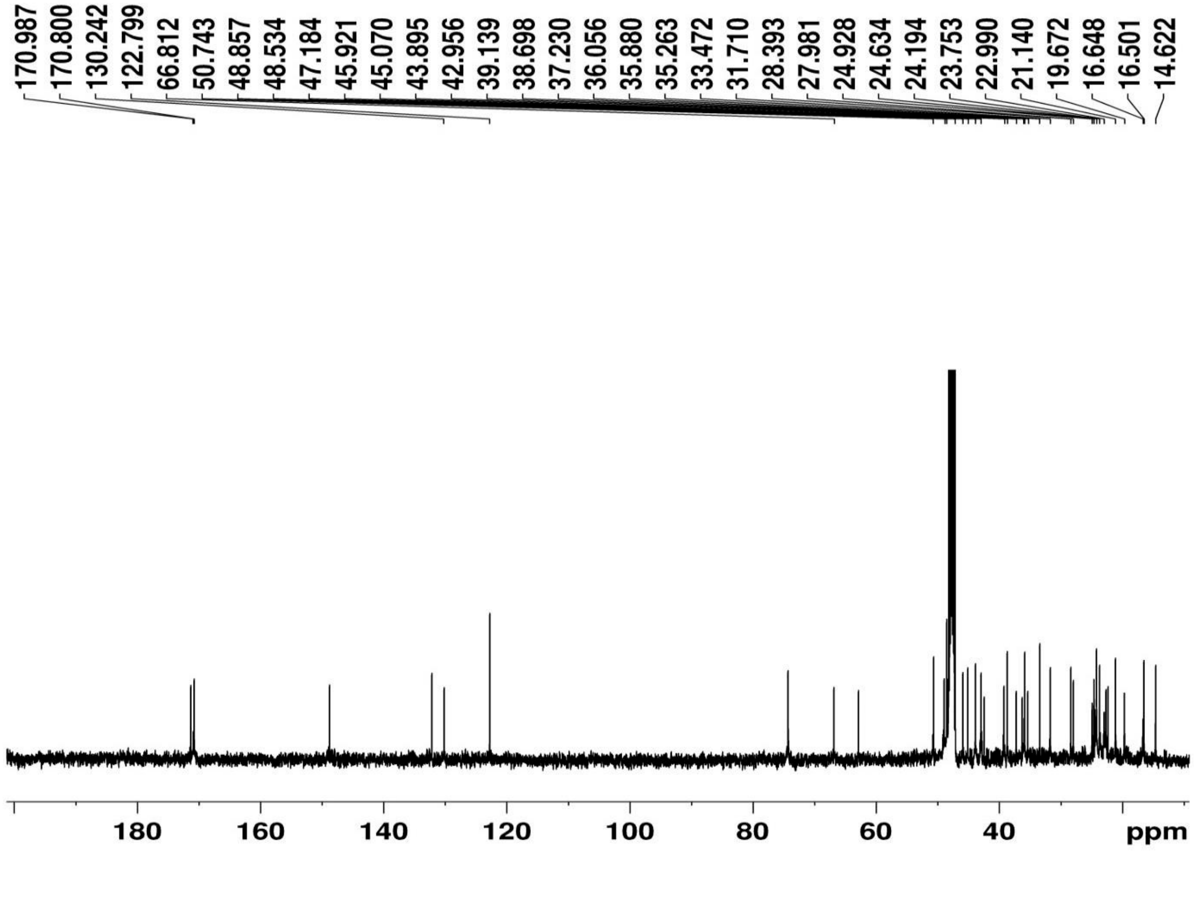

Supplement: Supplementary file 1 [file membranes-13-00309-s001.zip › membranes-2147104-supplementary.pdf]
